# A novel coordination mode of κ^1^-N-Br-pyridylbenz-(imida, oxa or othia)-zole to Pt(ii): synthesis, characterization, electrochemical and structural analysis†

**DOI:** 10.1039/c9ra01856e

**Published:** 2019-05-07

**Authors:** Juan Nicasio-Collazo, Gonzalo Ramírez-García, Marcos Flores-Álamo, Silvia Gutiérrez-Granados, Juan M. Peralta-Hernández, José Luis Maldonado, J. Oscar C. Jimenez-Halla, Oracio Serrano

**Affiliations:** Departamento de Química, Universidad de Guanajuato Cerro de La Venada S/N CP 36040 Guanajuato Gto Mexico oraciosinh@ugto.mx; Research Group of Optical Properties of Materials (GPOM), Centro de Investigaciones en Óptica AP 37000 León Guanajuato Mexico nicasio.collazo@cio.mx; Facultad de Química, Universidad Nacional Autónoma de México (UNAM) Circuito Exterior S/N, Coyoacán, Cd. Universitaria CP 04510 Ciudad de México Mexico

## Abstract

Herein, three novel Pt(ii) complexes with formula [*trans*-Pt(Br-PyBenz-X)(Cl)_2_(DMSO)] (1–3) having Br-pyridylbenz-(imida, oxa or othia)-zole (L_1–3_) derivatives as potential bidentate ligands, under an unusual κ^1^-N-coordination mode are reported. All compounds were obtained straightforwardly *via* reaction of corresponding L_PB1–3_ and [Pt(Cl)_2_(DMSO)_2_] (DMSO = dimethyl sulfoxide), at 100 °C in acetonitrile, respectively. 1–3 complexes were characterized by analytical and spectroscopic data: melting point, FT-IR, Raman, UV/Vis and NMR experiments. Cyclic voltammetry studies show an irreversible two-electron process at −0.50 and −0.51 V, which was ascribed to the Pt(ii)/Pt(iv) couple, for complexes 2 and 3. The crystal structure of complex 2 was elucidated by single-crystal X-ray diffraction, where the platinum atom exhibits a square plane geometry, where L_PB2_ adopts an unusual mono-coordinated mode *via* an N-κ^1^-benzoxazole ring. According to DFT calculations the first N-coordination exchanging one DMSO molecule is favourable, while the second N-coordination is highly impeded.

## Abstract 2

Three novel platinum(ii) complexes with the general formula [*trans*-Pt(Br-PyBenz-X)(Cl)_2_(DMSO)] containing Br-pyridylbenz(imida, oxa or othia)zole derivatives, under an unusual N-κ^1^-coordination mode were synthesized.

## Graphical abstract

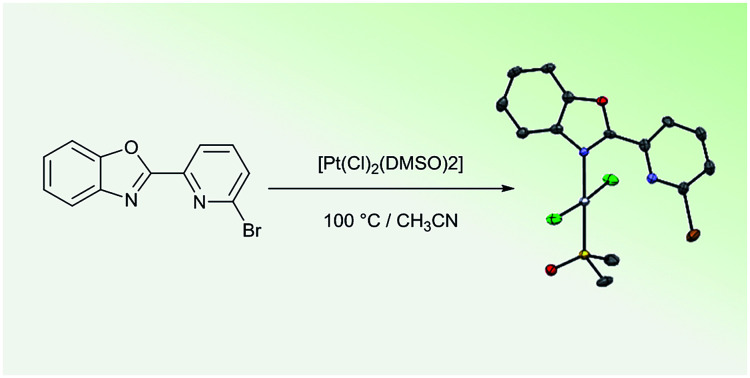

## Introduction

Benzimidazole and its derivatives are important heterocyclic compounds. These ligands have been found to exhibit biological activities and clinical applications.^1^ In the last decades, transition metal complexes with benzimidazole derivatives have shown attractive chemical properties^2,3^ and numerous applications in catalysis,^4–6^ biological activity,^7^ organic light-emitting diodes^8,9^ and photovoltaics.^10^ Furthermore, *N*,*N*′-donor chelating ligand pyridyl-benzimidazole has been used as a bipyridine analogue in a wide variety of complexes with different metal ions such as Ir(iii),^11,12^ Ru(ii),^13^ Pt(ii),^14^ respectively. Barnham and Ruiz *et al.*^11^ reported that a series of Ir(iii), Ru(ii) and Pt(ii) inhibited *in vitro* aggregation of the peptide fragment amyloid Aβ1–42, representing a potential use as therapeutic agents for Alzheimer's disease. Recently, Espino, Bolink and Orti *et al.*^12^ reported the synthesis of iridacycle complexes with pyridyl-imidazole ligands, which were used as electroluminescent materials in light-emitting electrochemical cells devices. Later, Wang *et al.*^14^ reported an elegant work on the synthesis of pyridyl-benzimidazole ligands, which display fluorescent emission in the purple/blue region at room temperature and phosphorescent emission in the blue/green region at 77 K. Fan *et al.*^15^ showed that W(0) species exhibit enhanced phosphorescence, at room temperature, from MLCT excited stated with quantum yields on the order of 10^−3^. Additionally, Housecroft and Bolink *et al.*^16^ reported an elegant synthesis of cyclometalated Ir(iii) compounds having pyridyl-benzoxa and thiazole derivatives (L_BP_), giving an efficient and stable red-emission, with active layers lifetimes greater than 1000 h. Furthermore, Ward *et al.*^13^ described a series of Re-carbonyl, Pt-acetylide and Ru-bipyridine complexes with L_BP_ ligands. Particularly, Pt species showed luminescence in the range 553–605 nm arising from the ^3^MLCT state, with emissive lifetime up to 500 ns and quantum yields up to 6% in CH_2_Cl_2_ at room temperature. Studies reported by DeStefano *et al.*^17,18^ on Pt- and Zn-L_BP_ complexes showed that they exhibit a color blue luminescence in the solid state and in solution.

**Chart 1:**
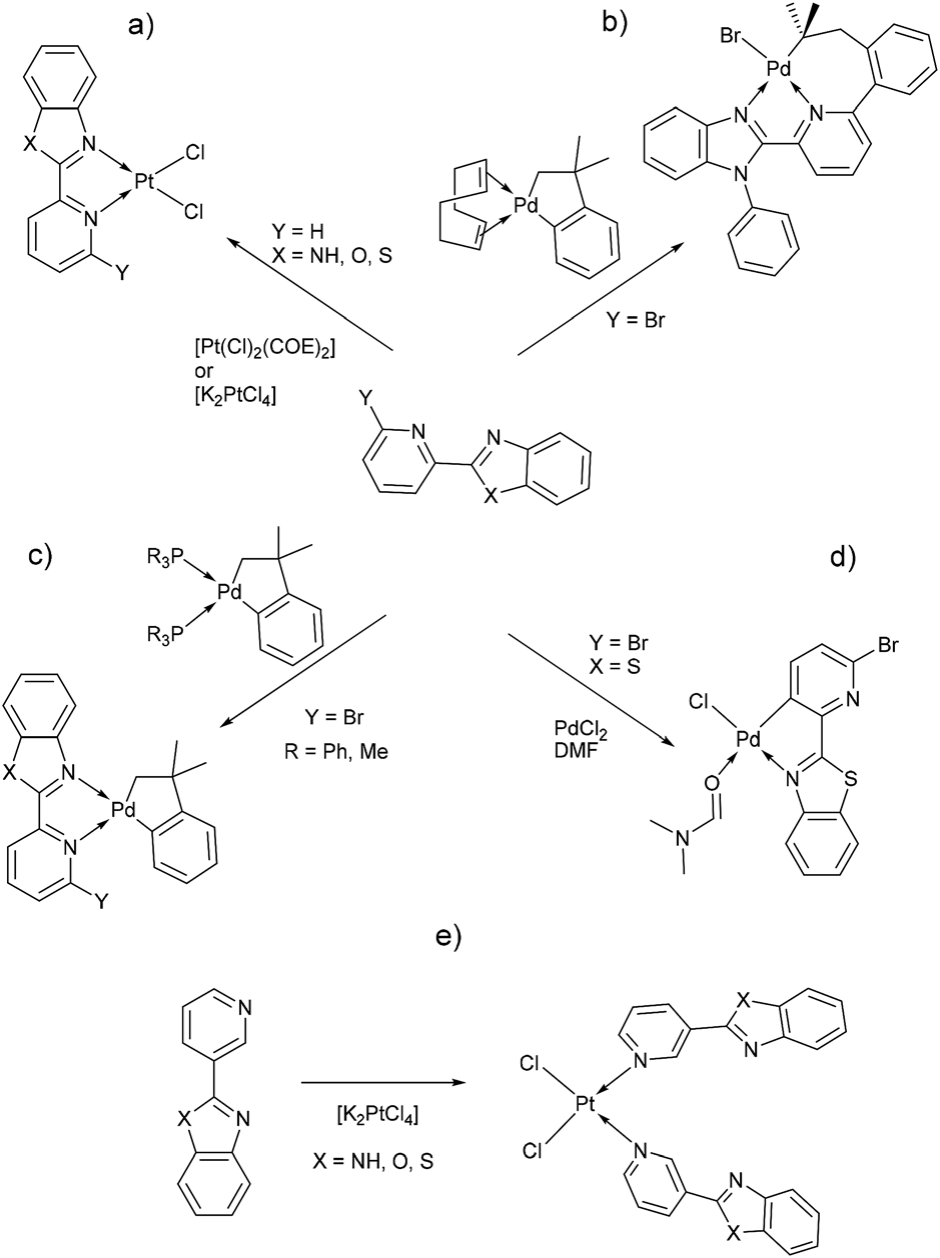


In early reports, Westcott *et al.*^7^ showed that the reaction of [Pt(Cl_2_)(COE)]_2_ (COE = *cis*-cyclooctene) or K_2_PtCl_4_ with L_PB_ ligands gives the expected [*cis*-Pt(Cl)_2_(L_PB_)] complexes (Chart 1a). Later, when benzimidazole fragment is in position three with respect to pyridine, the reaction lead to the monodentate [*cis*-Pt(Cl)_2_(L_PB_)_2_], where coordination to the metal occurs *via* the pyridine ring (Chart 1e). Part of our research has been focused on understanding the reactivity of 2-(6-bromopyridyl-2-yl)-1-phenylbenzimidazole, L_PB1_, ligand towards dialkyl–palladium complexes. A few years ago, we reported the synthesis of seven-membered palladacycles through selective C–Br bond activation of a Br-pyridine-benzimidazole derivative and concomitant C–C coupling bond, where no-innocent cyclooctadiene ligand (COD) plays an essential role (Chart 1b).^19^ Also, a five-membered palladacycles synthesis can be achieved when the COD is displaced by a phosphine ligand (Chart 1c). In 2018, we reported a direct synthesis on rollover palladacycles was achieved *via* C–H bond activation reaction in remote position on Br-pyridyl-benzothiazole (L_PB2_) by PdCl_2_, where the dimethylformamide plays an important role on the reaction pathway.^20^ However, to the best of our knowledge, well-characterized transition metal complexes containing L_PB_ ligands showing a N-κ^1^-coordinated mode still relatively scarce in the literature.^7^ Here in, we report our DFT calculations and experimental results on the reactivity of Br-Pyridylbenz-(imida, oxa or othia)-zole ligands toward platinum complexes.

## Results and discussion

Our first attempts were addressed to obtain a dialkyl–platinum complexes, through a stochiometric reaction of [Pt(COD)(Me)_2_] with L_PB1–3_, using CH_2_Cl_2_ as solvent at room temperature. However, under these conditions the reaction do not give the desired compound, instead, the starting materials are recovered. The replaced of dimethyl for an chloride–platinum complexes with general formula [PtCl_2_(COE)_2_] or [PtCl_2_(COD)] does not show a positive effect on the reactivity towards L_PB1–3_, according to the NMR analysis of the proton and platinum spectra. This suggest, that bromine atom into the L_PB1–3_ ligands plays an interesting role on the complex formation according to Westcott report's (Charts 1a and e). Later, when PPh_3_ was added to the reaction mixture, an spontaneous formation of complex [PtCl_2_(PPh_3_)_2_] takes place in quantitative yield, according to the ^31^P and ^195^Pt NMR experiments.^21^ Given the low reactivity of organoplatinum species, we carried out the reaction with Pt coordination species like [PtCl_2_(DMSO)_2_] or [PtCl_2_(THT)_2_] (DMSO = dimethyl sulfoxide; THT = tetrahydro-thiophene) with L_PB1–3_, retrieving the starting materials even under drastic reaction conditions (150 °C). Similar results have been described by Zucca *et al.*^22^

On the other hand, the use of K_2_PtCl_4_ with one equivalent of L_PB2_ in DMSO, did not show any reactivity under temperatures below 100 °C. When the temperature was increased to 150 °C, the ^1^H NMR spectrum showed only signals assigned to the free ligand. Nevertheless, the ^195^Pt NMR spectrum showed a new signal at −3526 ppm, differing from K[PtCl_3_(DMSO)] or [PtCl_2_(DMSO)_2_] signals which appeared at −2959 and −3450 ppm, respectively.^23^

Later, a stochiometric reaction of [PtCl_2_(COD)] with L_PB2_, in THT solution at 150 °C during a week, gave a yellow solid in a yellow solution. ^195^Pt NMR spectrum showed a signal at −3360 ppm, suggesting the formation of an analogous specie. Similar results were obtained when [PtCl_2_(COD)] complex was used as platinum source in solution of THT at 150 °C of temperature. Given the mentioned scarce reactivity, we decided to study the solvent effect of CH_2_Cl_2_, CHCl_3_, isopropyl alcohol or 2-ethoxyethanol at temperatures below to 100 °C, using [PtCl_2_(DMSO)_2_] as platinum source and one equivalent of L_PB2_; however, under these conditions the starting materials were recovered in quantitative amounts.

**Scheme 1:**
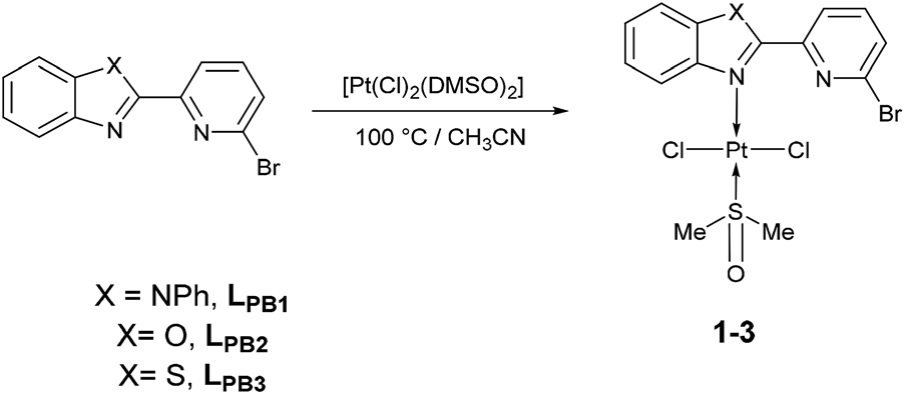
Synthetic route of [*trans*-PtCl_2_(DMSO)(L_PB1–3_)], 1–3, species.

To our delight, when the reaction was carried out, in acetonitrile at 100 °C, it leads to a pale yellow solid (Scheme 1). Temperature plays an important role, since a complex was not produced when the mixture was at temperatures below to 60 °C. Also, the solvent effect is not trivial since formation of metallic platinum was observed when isopropyl alcohol or 2-ethoxyethanol were used instead of acetonitrile.

Unfortunately, all compounds show very low solubility in common organic solvents, the NMR experiments were carried out in DMSO-*d*_6_. All experimental measurements were recorded immediately, after DMSO-*d*_6_ addition, in order to avoid ligand exchange between Pt(ii) complexes and DMSO-*d*_6_.^24,25^ Thus, under our conditions, only complexes 2–3 were soluble enough. The ^1^H NMR spectrum of 2 revealed a signal at 2.54 ppm with integrates to 6 protons, the signal was assigned to methyl protons, giving a singlet corresponding to DMSO fragment. In the region of 7.47 to 8.36 ppm seven signals appeared, assigned to aromatic protons corresponding to the L_PB2_ free fragment. ^13^C{^1^H} NMR spectrum showed a characteristic signal for the methyl group in 40.4 ppm assigned to the coordinate DMSO. In the aromatic region, four signals appeared in 120.5, 122.9, 130.6 and 140.9 ppm assigned to the carbons of phenyl fragment, and three signals in 111.5, 125.3 and 126.6 ppm assigned to the pyridyl fragment, according to 2D NMR experiments. Finally, the ^195^Pt NMR gave an intense signal at −3455 ppm, without any other significative signals, assuming the formation of only one platinum complex. The ^1^H NMR spectrum of complex 3 shows a singlet at 2.54 ppm, which is assigned to the methyl groups in the DMSO fragment. In the aromatic region between 7.53 to 8.35 ppm, seven well defined signals are observed and allocated into two sets: (i) three signals corresponding to the pyridine fragment (7.85, 7.99 and 8.35 ppm) and four signals for benzothiazole fragment (7.53, 7.59, 8.12 and 8.19 ppm). ^13^C{^1^H} NMR spectrum shows a signal at *δ* 40.4 ppm for methyl group of DMSO. Additionally, the signals at 119.8, 130.3 and 141.1 ppm are assigned to C–H for pyridine fragment and the signals at 122.7, 123.5, 126.3 and 126.9 are assigned to C–H in benzothiazole fragment, according to the 2D NMR ^1^H–^13^C. In good agreement with the latter, the ^195^Pt NMR spectrum shows one signal at −3455 ppm.

UV-Vis spectra of the L_PB1–3_ ligands showed a maximum absorption peak between 313–321 nm (Fig. S1†), which could be assigned to π → π* absorption (spin allowed S_0_ → ^1^IL transition).^26^ A hypochromic effect was observed in all UV-Vis spectra of 1–3, attributed to the Pt coordination towards the L_PB1–3_ ligands, respectively. Additional experimental evidence onto Pt-complexes synthesis was confirmed *via* melting point (decomposition to black solid), IR and Raman spectroscopies. Thus, the ligand bands of the complexes are slightly shifted with respect to free ligands. The main IR and Raman bands of complexes 1–3 are listed in ESI (see Table S1†). Identification of the *ν*(Pt–N), *ν*(Pt–Cl) and *ν*(Pt–S) at *ca.* 319, 330 and 283 cm^−1^ bands were compared among them and in relation to the [PtCl_2_(DMSO)_2_] complex.^27–29^

**Fig. 1:**
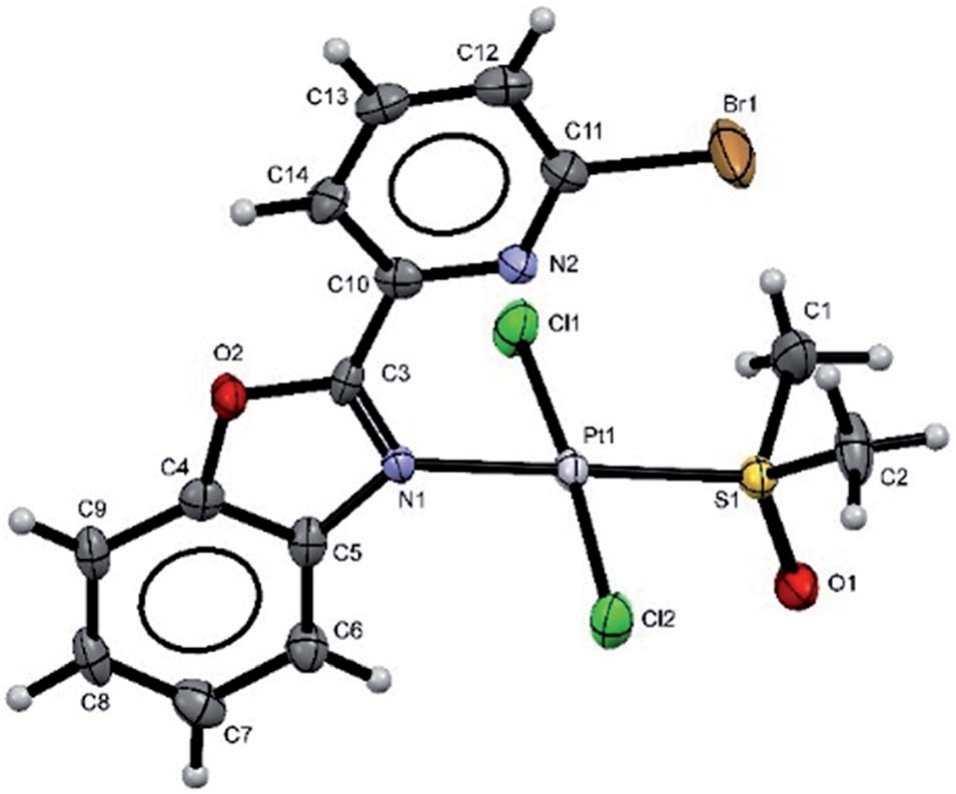
The molecular structure of complex 2 (the thermal ellipsoids are drawn at 60% probability). Selected bond lengths [Å] and angles (°); Cl(1)–Pt(1) 2.2990(12); Cl(2)–Pt(1) 2.3022(12); N(1)–Pt(1) 2.051(3); Pt(1)–S(1) 2.2108(11); N(1)–Pt(1)–S(1) 179.52(10); N(1)–Pt(1)–Cl(1) 87.00(10); S(1)–Pt(1)–Cl(1) 93.47(4); N(1)–Pt(1)–Cl(2) 89.68(10); S(1)–Pt(1)–Cl(2) 89.84(4); Cl(1)–Pt(1)–Cl(2) 174.85(5).

Additionally, suitable crystals of complex 2 were grown from slow evaporation of acetonitrile solution. Fig. 1 shows the molecular structure of compound 2. The Pt(ii) ion in [*trans*-PtCl_2_(L_2_)(DMSO)] exhibited distorted square plane geometry around Pt center where the ligand L_PB2_ was coordinated to the metal through a N atom in benzoxazole unit and the N atom of Br-pyridine remained free. The chlorine atoms adopted *trans* configuration, and the remaining position was occupied by the sulfur atom of the DMSO molecule coordinated to the metal center. Westcott *et al.*^7^ have reported the synthesis and characterization of *cis*-dichloroplatinum complexes containing mono coordinated piridynbenzimidazole, oxazole and thiazole derivatives were the coordination to metal center occurs *via* pyridine ring. To the best of our knowledge, our results demonstrated the influence of Br atom avoiding pyridin's nitrogen coordination to Pt metal center.

**Fig. 2:**
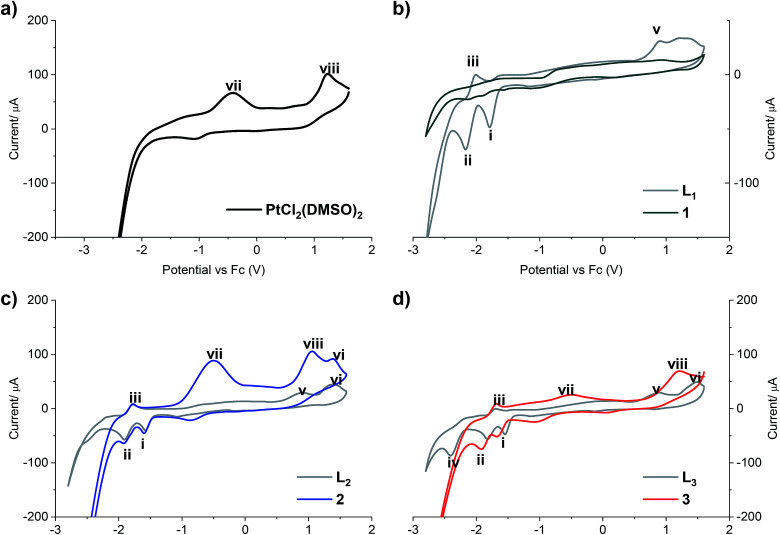
Cyclic voltammograms of (a) PtCl_2_(DMSO)_2_; (b) L_PB1_ free ligand and complex 1; (c) L_PB2_ free ligand and complex 2; (d) L_PB3_ free ligand and complex 3. Scan rate 300 mV s^−1^ cathodic direction.

The redox properties of all compounds were studied by cyclic voltammetry with a golden electrode in anhydrous DMF solution of 0.1 mol L^−1^ NBu_4_BF_4_ as the supporting electrolyte (Fig. 2).

Two reduction steps occur in all the analyzed free ligands and for complexes 2 and 3 (Fig. 2, peaks i and ii). First, the wave (i) is assigned to the irreversible reductive cleavage of the C–Br bond in the halopyridine moiety (see Table S2† for potential values).^30^ The wave (ii) is associated to wave (iii), and this couple is assigned to one-electron reversible redox transition response. This reduction/oxidation process is attributed to the N=C bond of the benz(imida, oxa or othia)zole rings, giving rise to stable radical anions in which nitrogen has the negative charge and the resulting carbon radical is stabilized by the adjacent heteroatom and the pyridine ring.^31^

An additional cathodic peak was observed at −2.4 V only when the free L_PB3_ ligand was analyzed (Fig. 2d). This irreversible reduction is expected to occur on the pyridine ring.^32^ However, we were unable to observe this wave for other ligands at the encompassed potentials in the electroactive window. It could be attributed to the strongest donating character of the benzothiazole, favoring the pyridine reduction with respect to the analogous benzoxazole or benzimidazole compounds, which include most electronegative heteroatoms. This shift towards more negative potentials was also observed by Kapturkiewicz *et al.*^26^ for unsubstituted 2-(2-pyridyl)benzazole ligands. In the case of complex 3, the mentioned peak at −2.4 V disappears due to the coordination of the N at the pyridine with the metal center of Pt(ii) (Fig. 2d). The peak (v), present in the cyclic voltammograms of all the free ligands at potentials between 887 and 916 mV is attributed to the irreversible oxidation of the nitrogen atom at the azole ring, which also coordinates with the platinum center, and in consequence it was not noticed when the complexes were analyzed. Furthermore, the wave (vi), observed when L_PB2_ and L_PB3_ ligands were analyzed, can be attributed to the irreversible oxidation of O and S, respectively (Fig. 2c and d). Such wave was not detected for L_PB1_ due to the presence of the phenyl substituents attached to the N. It should be noted that 1 shows very limited solubility in DMF. The absence of any peaks in voltammogram for complex 1 (Fig. 2b) is due to the immediate settling to the bottom of the cell when dispersed in solvent. Peak (vii) is only detected in the cyclic voltammograms of [PtCl_2_(DMSO)_2_], due an irreversible Pt(ii)/Pt(iv) oxidation. These experiments show that the coordination between the 2 or 3 and the platinum center does not have any effects on the oxidation state of the metal, remaining as Pt(ii). Finally, the peak (viii) is also observed only when the [PtCl_2_(DMSO)_2_] and its complexes where analyzed and it is assigned to the irreversible two-electron oxidation of the chloride anions.

**Fig. 3:**
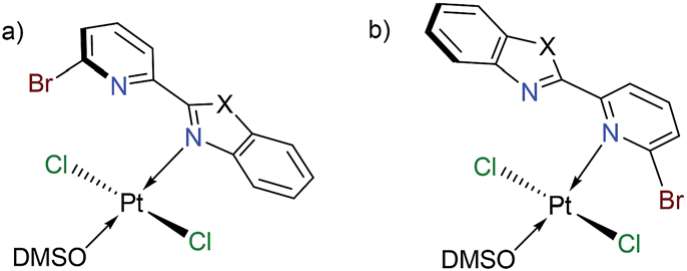
Platinum complexes having ligand L_BP1–3_ in a mono-coordination mode κ^1^-N.

Finally, we have performed theoretical calculations using the M06-L density-functional in combination with triple-ζ quality basis sets for carrying out geometry optimizations (see ESI† for further details). We have expressed energy values at the (SMD:DMSO)M06-L/def2-tzvpp level for discussing our results herein. Initially, we investigated the coordination mode for complexes [*trans*-PtCl_2_(DMSO)(L_BP1–3_)] (1–3). Since penta-coordinate moieties were reduced to well-known tetracoordinate complexes (Fig. 3a and b), we obtained that those of mode A were more stable than those of mode B for about 4 to 9 kcal mol^−1^ (see ESI†).

We conducted NBO calculations at the same level of theory, for complexes containing L_PB2_ ligand, aiming to understand this preference for coordination. We found that the N → Pt donation and back-donation are stronger for mode A stabilizing by 9.63 and 6.21 kcal mol^−1^, respectively, than for mode B according to our analysis (See ESI†). Although there is an increase in the Br → Pt donation in mode B stabilizing by 10.54 kcal mol^−1^ than for mode A, there is an increasing steric hindrance between Br and Cl vicinal atoms which deforms the square planar geometry of the metal centre. Furthermore, we calculated the reaction energies for the exchange of the *N*,*N*-donor ligand and solvent molecules in [PtCl_2_(DMSO)_2_] as shown in Table 1. The first N-coordination exchanging one DMSO molecule is favourable because of the negative values indicating exothermicity (and exergonicity) where the second N-coordination releasing the second DMSO is highly impeded. This is in line with our experimental evidence. The effect of substituents X = NPh, O or S; is also significant: heteroatoms with a more activating effect on the benz(imida, oxa or othia)zole ring make more available the lone-pair of the nitrogen that donates to the platinum.

**Table 1:** Calculated reaction energies (kcal mol^−1^) of the following reaction channels at the M06-L/mix-basis level^a^

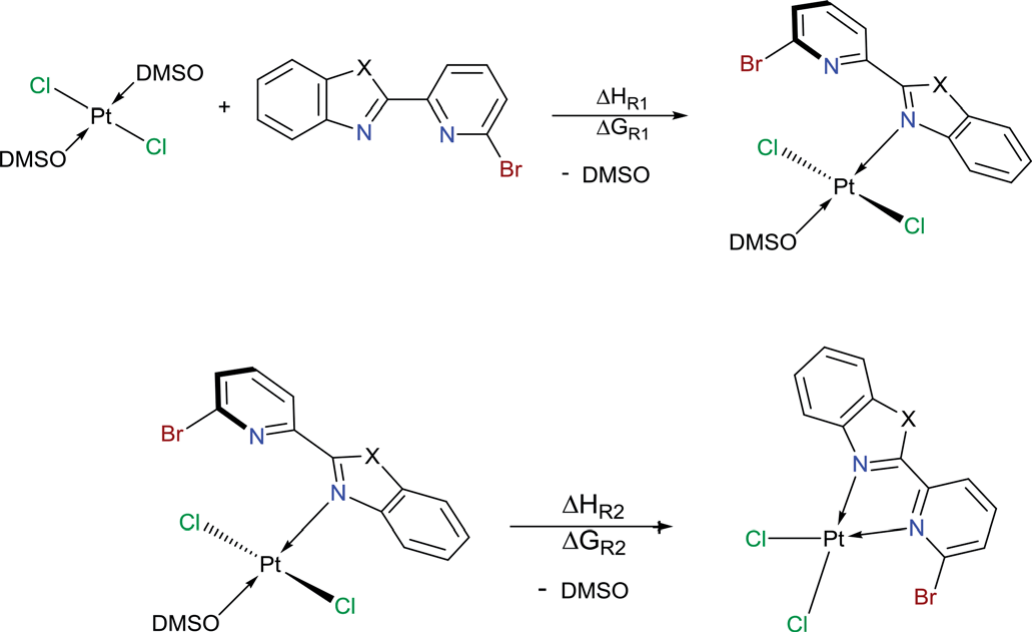				
X=	NC_6_H_5_	O	S	NH
Δ*H*_R1_	−6.2	−2.0	−1.9	−5.7
Δ*G*_R1_	−4.0	−0.4	0.4	−3.5
Δ*H*_R2_	17.6	18.4	16.6	16.1
Δ*G*_R2_	5.6	6.7	4.9	4.2

a Δ*H* and Δ*G* are in kcal mol^−1^.

Moreover, according to our calculations, the use of a more labile ligand like cyclooctene in order to promote its exchange with the second N-coordination (see eqn (S1)†) will not favour the reaction (Δ*H*_R_ = 18.0 kcal mol^−1^; Δ*G*_R_ = 6.0 kcal mol^−1^). We also corroborate that reported reactions using [PtCl_4_]^2−^ for obtaining the desired bidentate complexes (see eqn (S2)†) avoiding DMSO as solvent give exothermic (exergonic) reactions (Δ*H*_R_ = −14.8 kcal mol^−1^; Δ*G*_R_ = −17.4 kcal mol^−1^).

## Conclusions

In summary, we have demonstrated that reactivity of potential bidentate 2-(6-Br-2-pyridyl)-benz-(imida, oxa or othia)-zole ligands (L_PB1–3_) with an equivalent of [PtCl_2_(DMSO)_2_] complex, in acetonitrile at 100 °C, yields the complexes with general formula [*trans*-PtCl_2_(L_PB1–3_)(DMSO)], 1–3, respectively. L_PB1–3_ fragment ligands are bonded to the platinum center under an unusual N-κ^1^-coordination mode. Furthermore, cyclic voltammetry studies show an irreversible two-electron process at −0.50 and −0.51 V, which was ascribed to the Pt(ii)/Pt(iv) couple, for complexes 2 and 3. According to performance theoretical calculation the first N-coordination exchanging one DMSO molecule is favorable, under our experimental conditions, and the second N-coordination releasing the second DMSO is highly impeded. Finally, we demonstrated that effect of solvent, temperature, the bromine atom at the auxiliary ligands and platinum complexes source kind, plays an important role in the formation on this unusual mononuclear species.

## Experimental

### General considerations

Manipulations of air and moisture compounds were performed under nitrogen atmosphere using standard Schlenk techniques. Acetonitrile was dried over 3 Å molecular sieves before use. K_2_PtCl_4_ was purchased from Pressure Chemical Co (USA). All materials were used as received from commercial suppliers without further purification. Compounds L_PB1_,^19^L_PB2_,^26^L_PB3_,^33–35^ and [Pt(Cl)_2_(DMSO)_2_]^36^ were prepared according to the literature. NMR spectra were recorded on a Bruker Ultra Shield 500 and Bruker AV-400 MHz spectrometer at room temperature. ^1^H and ^13^C{^1^H} chemical shift (*δ*) are given in part per million (ppm) relative to tetramethylsilane (TMS) and the solvent resonance were used as internal standards and ^195^Pt relative to K_2_PtCl_4_ (Solution in D_2_O). Elemental analysis experiments were carried out on 2400 CHNS/O Series II System PerkinElmer. IR spectra were recorded on a PerkinElmer Spectrum 100 FT-IR infrared spectrometer using KBr pellets. Raman spectra were recorded on a Renishaw InVia microscope Raman spectrometer. Raman measurements were acquired with an excitation line provided by a diode laser at 457 nm, by means of a 20× microscope's objective with a 0.4 numerical aperture; 2 s of integration time, and 20 accumulations were selected. UV-Vis absorption spectrum was collected using a spectrophotometer Perking Elmer, lambda 900, Waltham, MA, USA. Cyclic voltammetry studies were conducted at room temperature in a BASi Epsilon-EC potentiostat/galvanostat with included software system. A gold working electrode (2 mm), a platinum wire auxiliary electrode, and Ag/AgCl reference electrode were used in a standard three-electrode configuration. The measurements were carried out in 5 mL of DMF solutions of the compound (3.0 × 10^−3^ mol L^−1^) containing 0.05 mol L^−1^ tetrabuthylammonium tetrafluoroborate (NBu_4_BF_4_) as the supporting electrolyte. X-ray crystallographic studies were collected at Oxford Diffraction Gemini “A” diffractometer.

### Synthesis of ligands

#### 2-(6-Bromopyridin-2-yl)-1-phenyl-1*H*-benzimidazole (L_PB1_)

*N*-Phenyl-*o*-phenylenediamine (533 mg, 3 mmol) and 6-bromopyridincarboxaldehyde (558 mg, 3 mmol) were dissolved in 20 mL of ethanol. Then, the reaction mixture was stirred at room temperature in an open flask and monitored by TLC. On the completion of the reaction, solvent was removed, and the crude product reaction was dissolved in DMF (10 mL) and NaCN (147 mg, 3 mmol) was added. Then, the reaction was stirred at room temperature in an open flask and monitored by TLC. On the completion on the reaction, the mixture was quenched with water and extracted with diethyl ether. The organic phase was collected, dried over MgSO_4_, and concentrated. The product was purified by flash column chromatography on silica gel with hexanes/ethyl acetate as (6 : 1) eluent, yielding a brown powder (753 mg, 2.15 mmol). Yield 80%. Mp = 112–114 °C. ^1^H NMR (CDCl_3_, 500 MHz): *δ* = 8.16 (d, 1H, *J* = 7.7 Hz), 7.90 (d, 1H, *J* = 8.0 Hz), 7.58 (t, 1H, *J* = 7.8 Hz), 7.55–7.46 (m, 4H), 7.41–7.26 (m, 7H), 7.21 (d, 1H, *J* = 8.0 Hz) ppm. ^13^C{^1^H} NMR (CDCl_3_, 126 MHz): *δ* = 149.96, 148.77, 142.69, 140.76, 138.76, 138.06, 137.58, 129.31, 128.50, 128.18, 127.71, 124.42, 123.45, 123.02, 120.38, 111.21 ppm. IR (KBr) *ν̃* = 3042, 1595, 1577, 1557, 1501, 1435, 1389, 1328, 1267, 1200, 1151, 1117, 1072, 988, 846, 797, 760, 738, 699, 656, 624, 573 cm^−1^.

#### 2-(6-Bromopyridin-2-yl)benzoxazole (L_PB2_)

The synthesis was similar to that described for L_1_, and the starting materials were 2-aminophenol (327 mg, 3 mmol) and 6-bromo-2-pyridinecarboxaldehyde (558 mg, 3 mmol), and NaCl (147 mg, 3 mmol), to afford a white solid (310 mg, 1.1 mmol). Yield 43%. ^1^H NMR (CDCl_3_, 500 MHz): *δ* = 8.26 (dd, 1H, *J* = 7.6, 1.0 Hz), 7.83–7.77 (m, 1H), 7.70 (t, 1H, *J* = 7.8 Hz), 7.63 (dd, 1H, *J* = 7.2, 1.9 Hz), 7.60 (dd, 1H, *J* = 7.9, 0.9 Hz), 7.37 (pd, 2H, *J* = 7.4, 1.5 Hz) ppm. ^13^C{^1^H} NMR (CDCl_3_, 126 MHz): *δ* = 160.05, 151.14, 146.82, 142.63, 141.69, 139.27, 130.24, 126.46, 125.16, 122.31, 120.85, 111.48 ppm. IR (KBr) *ν̃* = 3049, 1576, 1549, 1509, 1421, 1315, 1249, 1154, 1120, 996, 988, 864, 794, 757, 643, 531 cm^−1^. Mp = 182–184 °C.

#### 2-(6-Bromopyridin-2-yl)benzothiazole (L_PB3_)

2-Aminothiophenol (321 mg, 3 mmol) and 6-bromo-2-pyridinecarboxaldehyde (558 mg, 3 mmol) were dissolved in 20 mL of ethanol, the reaction mixture was stirred over night at room temperature, the product precipitates from ethanol without any further purification, to afford a pale yellow solid (856 mg, 295 mmol). Yield 98%. Mp = 192–194 °C. ^1^H NMR (CDCl_3_, 500 MHz): *δ* = 8.32 (dd, 1H, *J* = 7.8, 0.9 Hz), 8.09 (d, 1H, *J* = 8.1 Hz), 7.99–7.91 (m, 1H), 7.70 (t, 1H, *J* = 7.7 Hz), 7.56 (d, 1H, *J* = 7.9 Hz), 7.56–7.48 (m, 1H), 7.44 (t, 1H, *J* = 7.6 Hz) ppm. ^13^C{^1^H} NMR (CDCl_3_, 126 MHz): *δ* = 167.59, 154.29, 152.54, 141.86, 139.33, 136.48, 129.75, 126.60, 126.10, 123.88, 122.22, 119.59 ppm. IR (KBr) *ν̃* = 3092, 3062, 3045, 1578, 1541, 1475, 1452, 1427, 1408, 1349, 1281, 1247, 1238, 1159, 1131, 1111, 1085, 1075, 1005, 983, 929, 891, 818, 759, 738, 649, 623 cm^−1^.

### General procedure for the synthesis of [*trans*-Pt(Cl)_2_(L_*n*_)(DMSO)]

In a sealed tube was charged with L_PB*n*_ (0.120 mmol), [Pt(Cl)_2_(DMSO)_2_] (0.120 mmol) and acetonitrile (15 mL). The reaction mixture was heated at 100 °C for 24 h and cooled down slowly to room temperature. Thus, a solid precipitate was observed. The solid was filtered and washed with Et_2_O giving the platinum complexes in good yield. All complexes were obtained in gram scale with good yields (up to 70%) and were characterized by FTIR, Raman, NMR, UV-Vis spectroscopies and melting point.

#### [*trans*-Pt(Cl)_2_(L_1_)(DMSO)] (1)

The synthesis was following the general procedure, ligand L_PB1_ (500 mg, 1.4 mmol) was reacted with [PtCl_2_(DMSO)_2_] (603 mg, 1.4 mmol) giving a pale-yellow solid (649 mg, 1 mmol). Yield 70%. C_20_H_18_BrCl_2_N_3_OPtS (694.33 mol^−1^): calcd. C 34.60; H 2.61; N 6.05; found C 34.66; H 2.57, N 5.93. Mp = 206–208 °C. IR (*ν̃*) = (3091, 3064, C–H) 1595, 1582, 1557, 1500, 1456, 1445, 1407, 1389, 1333 (C–N), 1265, 1208, 1167, 1152, 1122 (S=O) cm^−1^.

#### [*trans*-Pt(Cl)_2_(L_2_)(DMSO)] (2)

The synthesis was following the general procedure, ligand L_PB2_ (500 mg, 1.8 mmol) was reacted with [Pt(Cl)_2_(DMSO)_2_] (767 mg, 1.8 mmol), to afford a brown solid (1070 mg, 1.7 mmol). Yield 95%. C_14_H_13_BrCl_2_N_2_O_2_PtS (619.22 mol^−1^): calcd. C 27.16; H 2.12; N 4.52; found C 27.45; H 1.98, N 4.49. Mp = 196–198 °C. ^1^H NMR (500 MHz, DMSO-*d*_6_) *δ* = 8.36 (dd, *J* = 7.6, 0.8, 1H), 8.02 (t, *J* = 7.8, 1H), 7.93–7.86 (m, 3H), 7.52 (td, *J* = 7.6, 1.2, 1H), 7.47 (td, *J* = 7.6, 1.2, 1H), 2.54 (s, 6H) ppm. ^13^C{^1^H} NMR (126 MHz, DMSO-*d*_6_) *δ* = 159.81, 150.49, 145.93, 141.65, 141.07, 140.95, 130.62, 126.68, 125.36, 122.96, 120.52, 111.52, 40.43 ppm. ^195^Pt NMR (107 MHz, DMSO-*d*_6_) *δ* = −3455 ppm. IR (*ν̃*) = (3037, 3009, C–H) 2991, 2926, 2917, 1534, 1401, 1354, 1305 (S–C), 1299 (C–N), 1156, 1133, 1020 (S=O) cm^−1^.

#### [*trans*-Pt(Cl)_2_(L_3_)(DMSO)] (3)

The synthesis was following the general procedure, ligand L_PB2_ (500 mg, 1.7 mmol) was reacted with [Pt(Cl)_2_(DMSO)_2_] (725 mg, 1.7 mmol), to afford a brown solid (1050 mg, 1.6 mmol). Yield 96%. Mp = 204–206 °C. C_14_H_13_BrCl_2_N_2_OPtS_2_ (635.28 mol^−1^): calcd. C 26.47; H 2.06; N 4.41; found C 26.71; H 1.91, N 4.39.^1^H NMR (500 MHz, DMSO-*d*_6_) *δ* = 8.35 (dd, *J* = 7.6, 0.8, 1H), 8.19 (dd, *J* = 7.9, 1.5, 1H), 8.12 (d, *J* = 7.7, 1H), 7.99 (t, *J* = 7.8, 1H), 7.85 (dd, *J* = 7.9, 0.8, 1H), 7.59 (ddd, *J* = 8.3, 7.2, 1.3, 1H), 7.53 (ddd, *J* = 8.3, 7.1, 1.2, 1H), 2.54 (s, 6H) ppm. ^13^C{^1^H} NMR (126 MHz, DMSO-*d*_6_) *δ* = 166.82, 153.61, 151.47, 141.10, 135.46, 130.34, 126.91, 126.37, 123.52, 122.74, 119.82, 40.43 ppm. ^195^Pt NMR (107 MHz, DMSO-*d*_6_) *δ* = −3455 ppm. IR (*ν̃*) = (3037, 3009, C–H), 2992, 2926, 2917, 1401, 1384, 1305 (S–C), 1299 (C–N), 1156, 1133, 1020 (S=O) cm^−1^.

### X-ray crystallography

X-ray powder diffraction experiments were acquired in Diffractometer Model D2 PHASER-Bruker. Single crystal of complex was mounted on a glass fiber and analyzed with an Oxford Diffraction Gemini “A” diffractometer with a CCD area detector, a sealed tube X-ray source (*λ*_MoKα_ = 0.71073 Å), and a graphite monochromator. The double pass method of scanning was used to exclude any noise. The collected frames were integrated with the CrysAlis Pro and CryAlis RED software package (Agilent Technologies); analysis of the integrated data did not reveal any decay. Final cell parameters were determined by a global refinement of reflections; data were corrected for absorbance by analytical numeric absorption correction,^37^ employing a multifaceted crystal model based on expression upon the Laue symmetry using equivalent reflection. Structure solution and refinement were carried out with SHELXS-2014 and SHELXL-2014;^38,39^ Mercury CSD 39 and the WinGX suite were used for molecular graphics and presentation of single-crystal diffraction data.^40,41^ Full-matrix least-squares refinement was carried out by minimizing (*F*_o_^2^ = *F*_c_^2^)^2^. All non-hydrogen atoms were refined anisotropically. Crystallographic data have been deposited at the Cambridge Crystallographic Data Center as ESI† number CCDC 1849220.

## Conflicts of interest

There are no conflicts to declare.

## Supplementary Material

RA-009-C9RA01856E-s001

RA-009-C9RA01856E-s002
