# Supplementary material for: A novel coordination mode of κ1-N-Br-pyridylbenz-(imida, oxa or othia)-zole to Pt(ii): synthesis, characterization, electrochemical and structural analysis
Source: RSC Adv. 2019 May 7;9(25):14033–9. doi: 10.1039/c9ra01856e (PMC9064021; doi:10.1039/c9ra01856e)
Supplement: RA-009-C9RA01856E-s001 [file RA-009-C9RA01856E-s001.pdf]

Electronic Supplementary Material (ESI) for RSC Advances.
This journal is © The Royal Society of Chemistry 2019

Supplementary Information

for:

A novel coordination mode κ^1-N-Br-Pyridylbenz-(imida, oxa or othia)-zole to Pt(II): Synthesis, Characterization, Electrochemical and Structural Analysis

Juan Nicasio-Collazo,[a,b] Gonzalo Ramírez-García,[a] Marcos Flores-Álamo,[c] Silvia Gutiérrez-Granados,[a] Juan M. Peralta-Hernández,[a] José Luis Maldonado,[b] J. Oscar C. Jimenez-Halla,[a] and Oracio Serrano*[a]*

a Departamento de Química, Universidad de Guanajuato, Cerro de la Venada S/N, CP 36040, Guanajuato, Gto. México. E-mail: nicasio.collazo@cio.mx, oraciosinh@ugto.mx

b Research Group of Optical Properties of Materials (GPOM), Centro de Investigaciones en Óptica, A.P. 37000, León, Guanajuato, México

c Facultad de Química, Universidad Nacional Autónoma de México (UNAM), Circuito Exterior S/N, Coyoacán, Cd. Universitaria, CP 04510, Ciudad de México, México.

UV-Vis Spectra

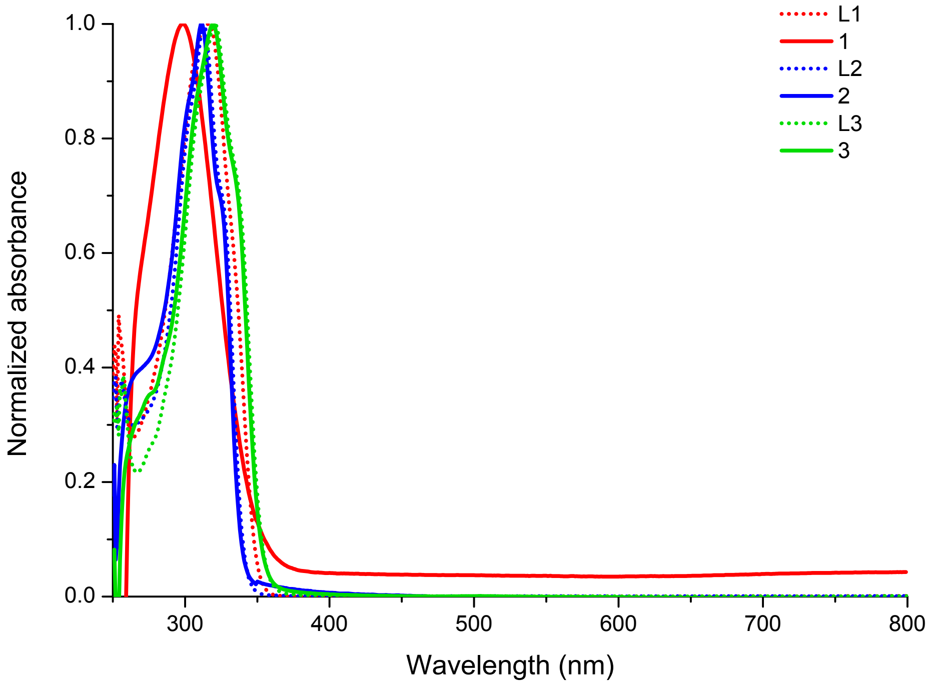

Figure S1. UV-Vis spectra of ligands $\mathbf{L_{1\text{-}3}}$ and complexes [*trans*-$PtCl_2$(DMSO)($L_{1\text{-}3}$)] in a solution of DMF.

Table S1. Assignment for the most characteristic IR and Raman band of complexes [trans-$Pt(Cl)_2(L_{1-3})(DMSO)$].

**1**		**L_1**	**2**		**L_2**	**3**		**L_3**	
IR	*Raman*	*IR*	*IR*	*Raman*	*IR*	*IR*	*Raman*	*IR*	*Assignment*
3064	-	3042	3037	-	3049	3037	-	3062	ν (CH_{aryl})
1609	1599	1597	-	-	-	-	-	-	ν (CH_{ring})
1595	1582	1577	-	-	-	-	-	-	ν (CH_{ring})
1445	-	1435	1412	-	1421	1412	-	1408	δ_a (CH_3)
1389	1402	1389	1384	1394	1315	1384	1394	1349	δ (CH_3)
1152	-	-	1156	1150	-	1156	1150	-	ν_a (SO)
1126	1135	-	1133	-	-	1133	1133	-	ν_s (SO)
697	657	699	690	697	643	690	697	649	ν (C-Br)
-	330	-	-	334	-	-	334	-	ν_s (Pt-Cl)
-	283	-	-	273	-	-	274	-	ν_s (Pt-S)

ν and δ means stretching and bending respectively, $_a$ means asymmetric, $_s$ means symmetric

Electrochemical data

Table S2. *Electrochemical data free ligand and Pt(II) complexes.*

**Compounds**	**E_p (V)**

	i	Ii	Iii	Iv	V	Vi	Vii	viii
**$PtCl_2(DMSO)_2$**	-	-	-	-	-	-	-0.4	1.3
**L_2**	-1.6	-1.9	-1.8	-	0.9	1.4	-	-
**2**	-1.6	-1.9	-1.8	-	-	1.4	-0.5	1.1
**L_3**	-1.5	-1.8	-1.7	-2.4	0.9	1.5	-	-
**3**	-1.7	-1.9	-1.7	-	-	-	-0.5	1.2
**L_1**	-1.8	-2.2	-2.0	-	0.9	-	-	-
**1**	-	-	-	-	-	-	-	-

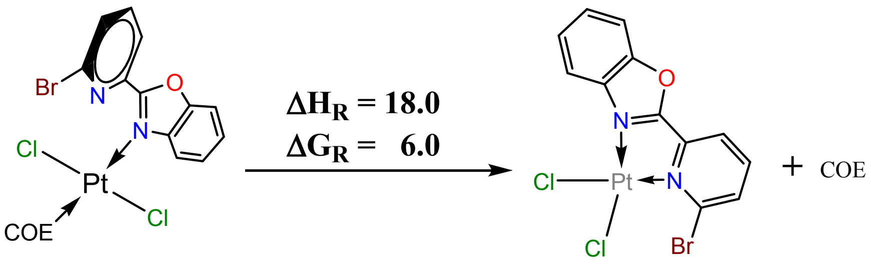

Eq. S1. COE = cyclooctene.

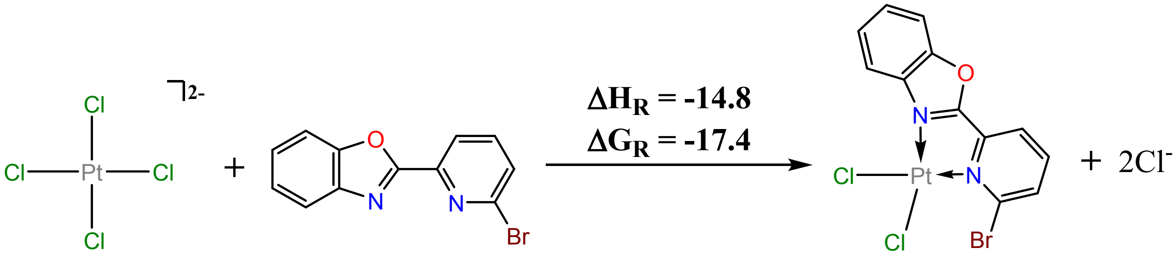

Eq. S2.

Calculated reaction energies are shown in kcal·mol^{-1}.

NMR Spectra

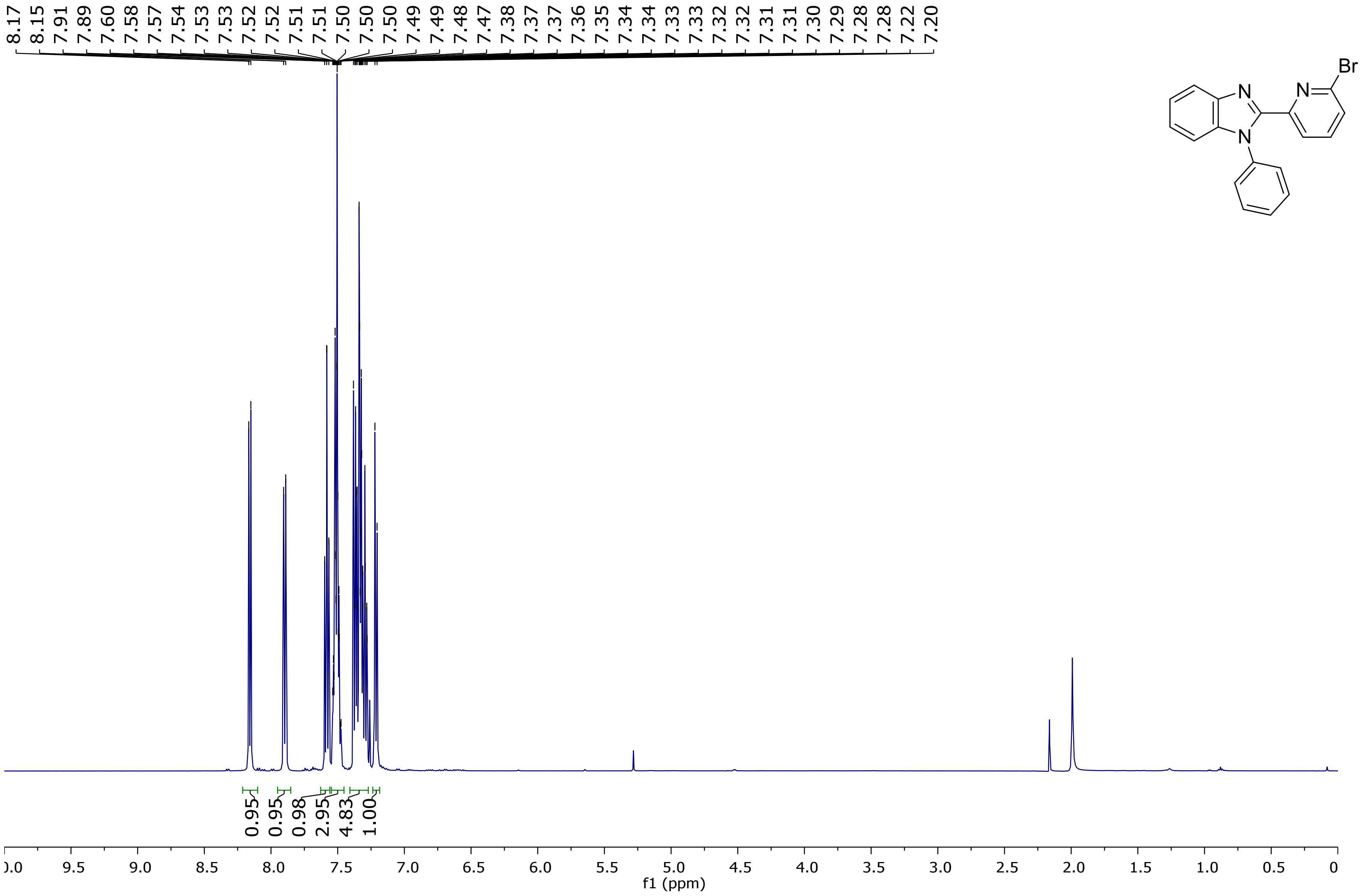

Fig. S2. ^{1}H NMR spectrum of **L_1** in $CDCl_3$ at RT

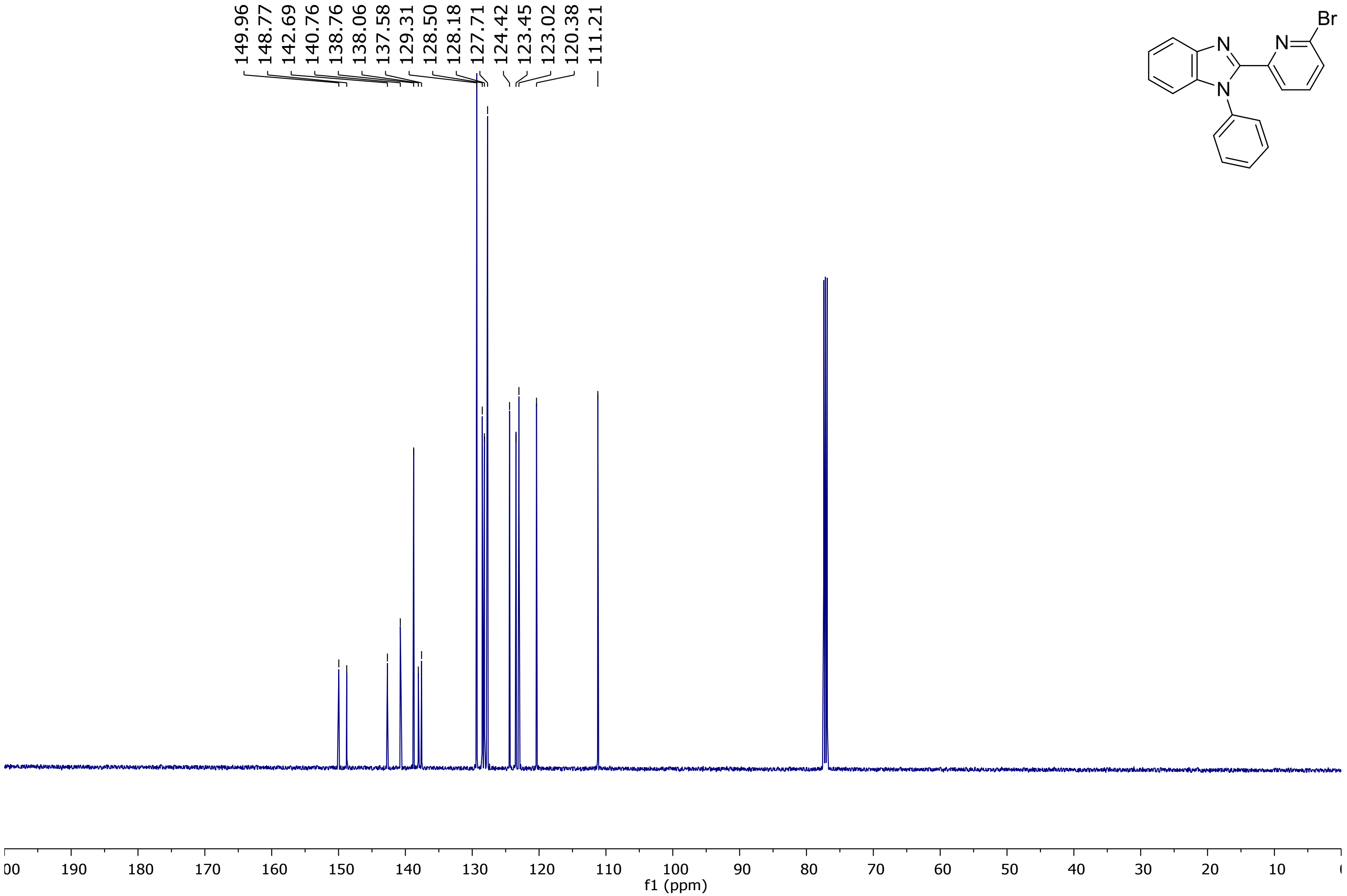

Fig. S3. ^{1}H NMR spectrum of $\mathbf{L_1}$ in $CDCl_3$ at RT

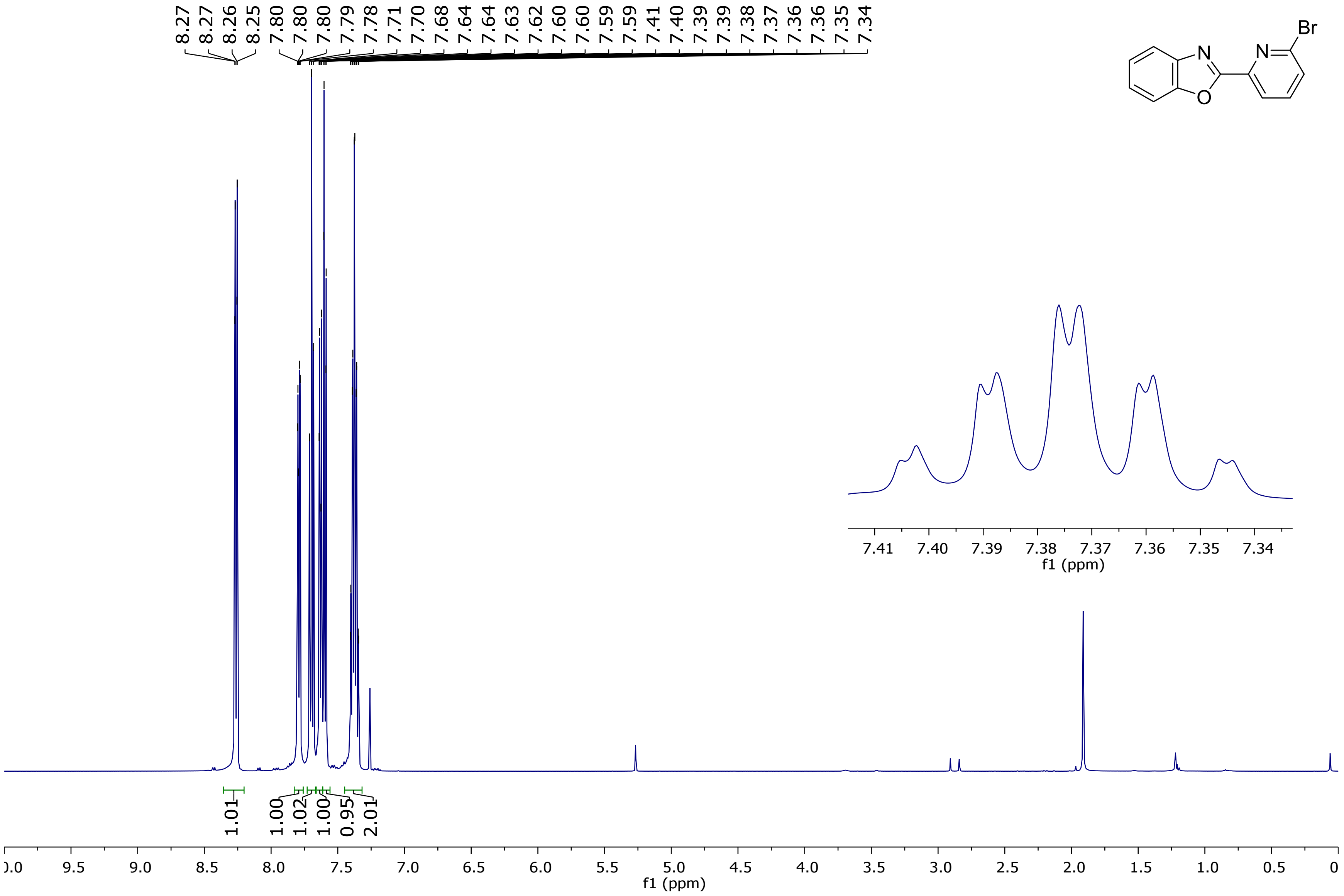

Fig. S4. ^{13}C NMR spectrum of $\mathbf{L_2}$ in $CDCl_3$ at RT

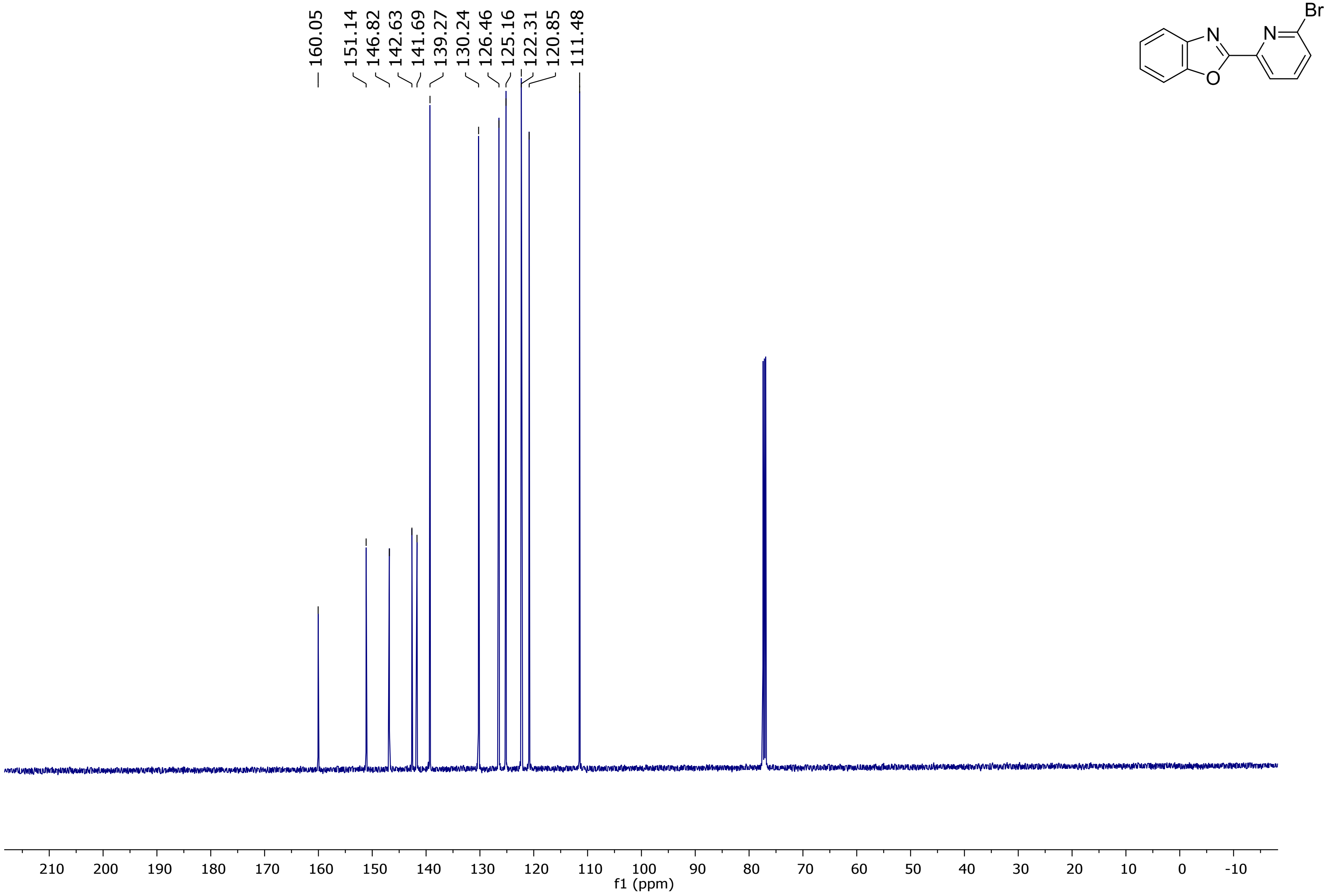

Fig. S5. ^{13}C NMR spectrum of $\mathbf{L_2}$ in $CDCl_3$ at RT

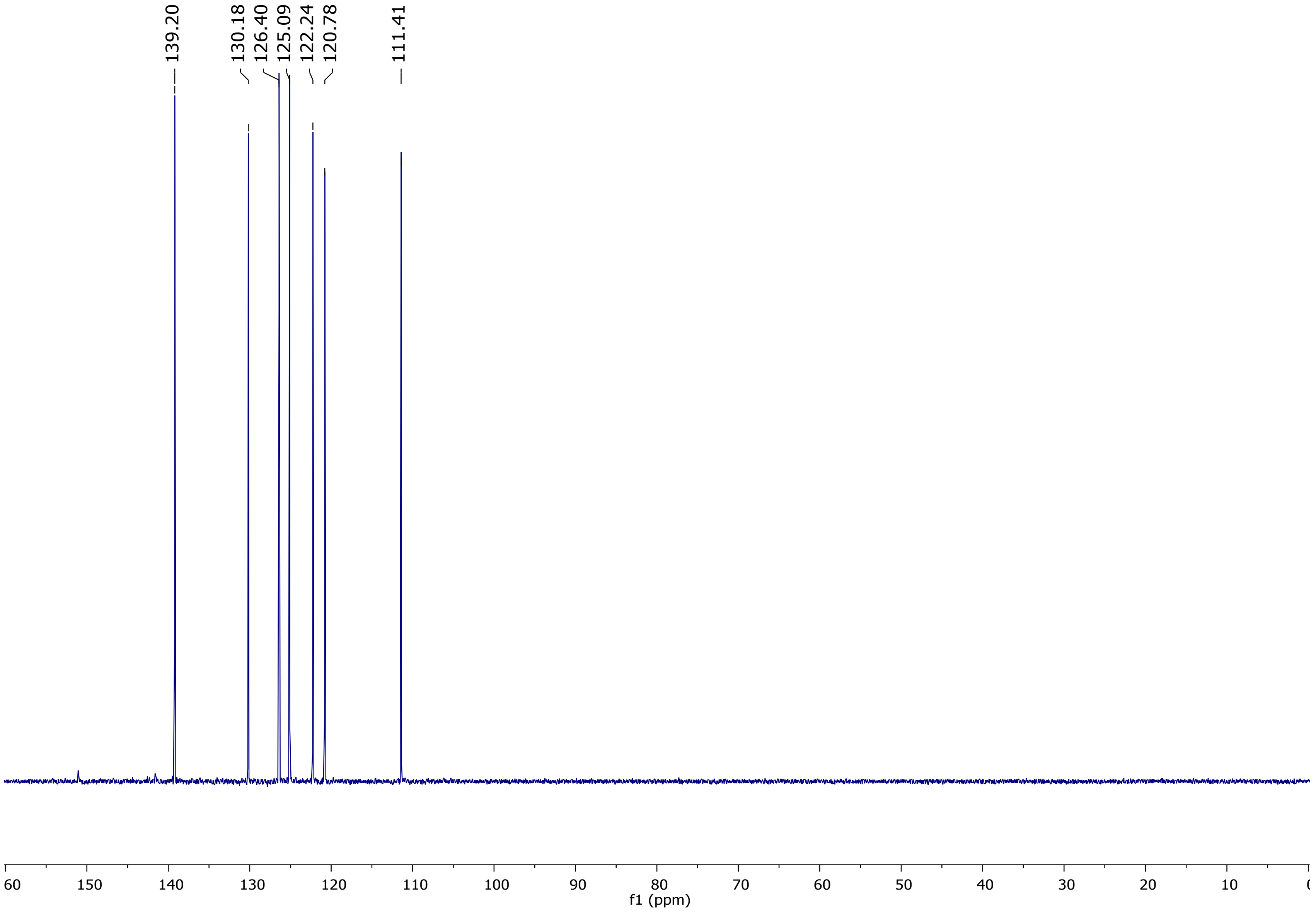

Fig. S6. ^{13}C DEPT NMR spectrum of $\mathbf{L_2}$ in $CDCl_3$ at RT

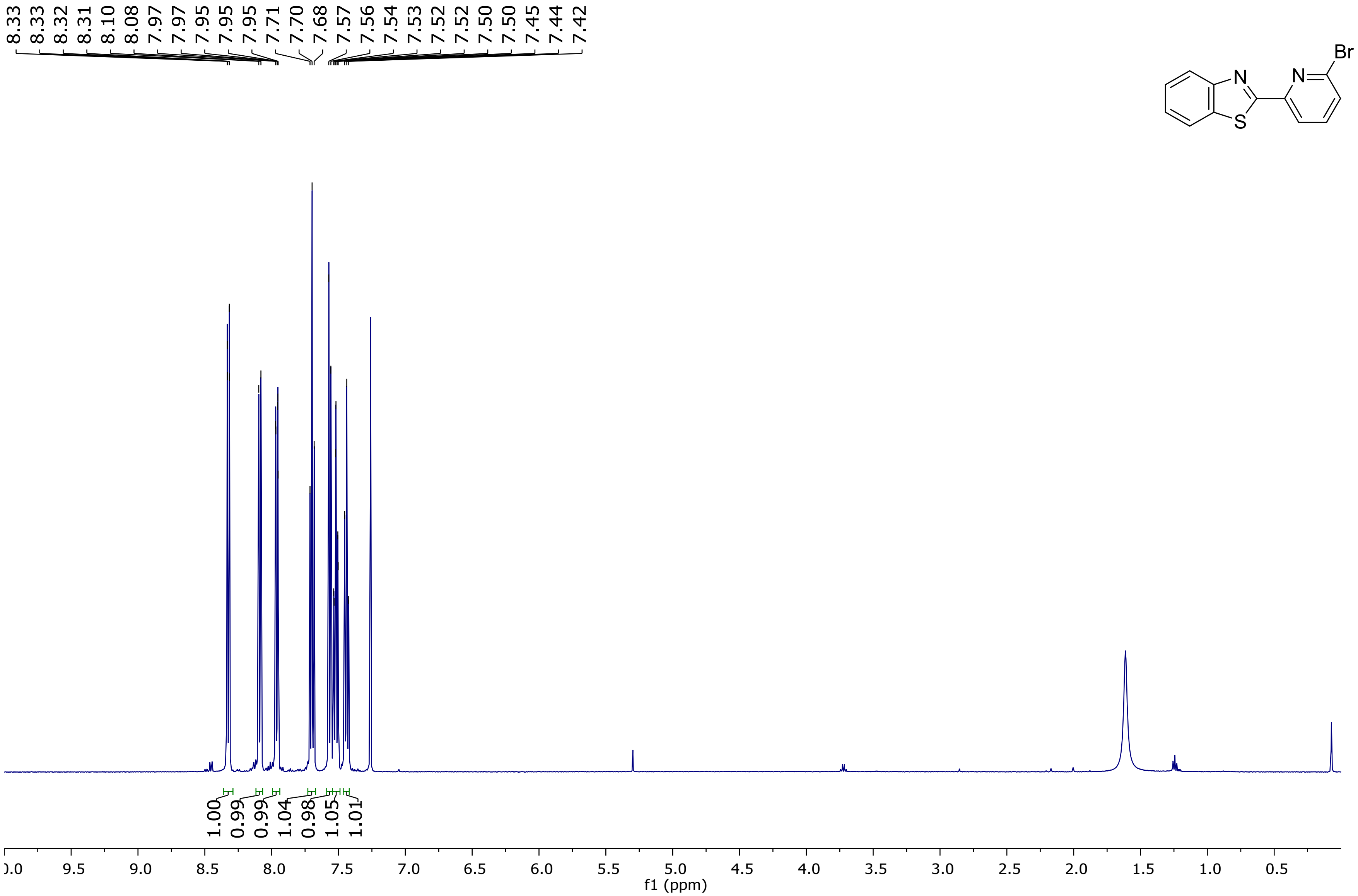

Fig. S7. ^{1}H NMR spectrum of $\mathbf{L_3}$ in $CDCl_3$ at RT

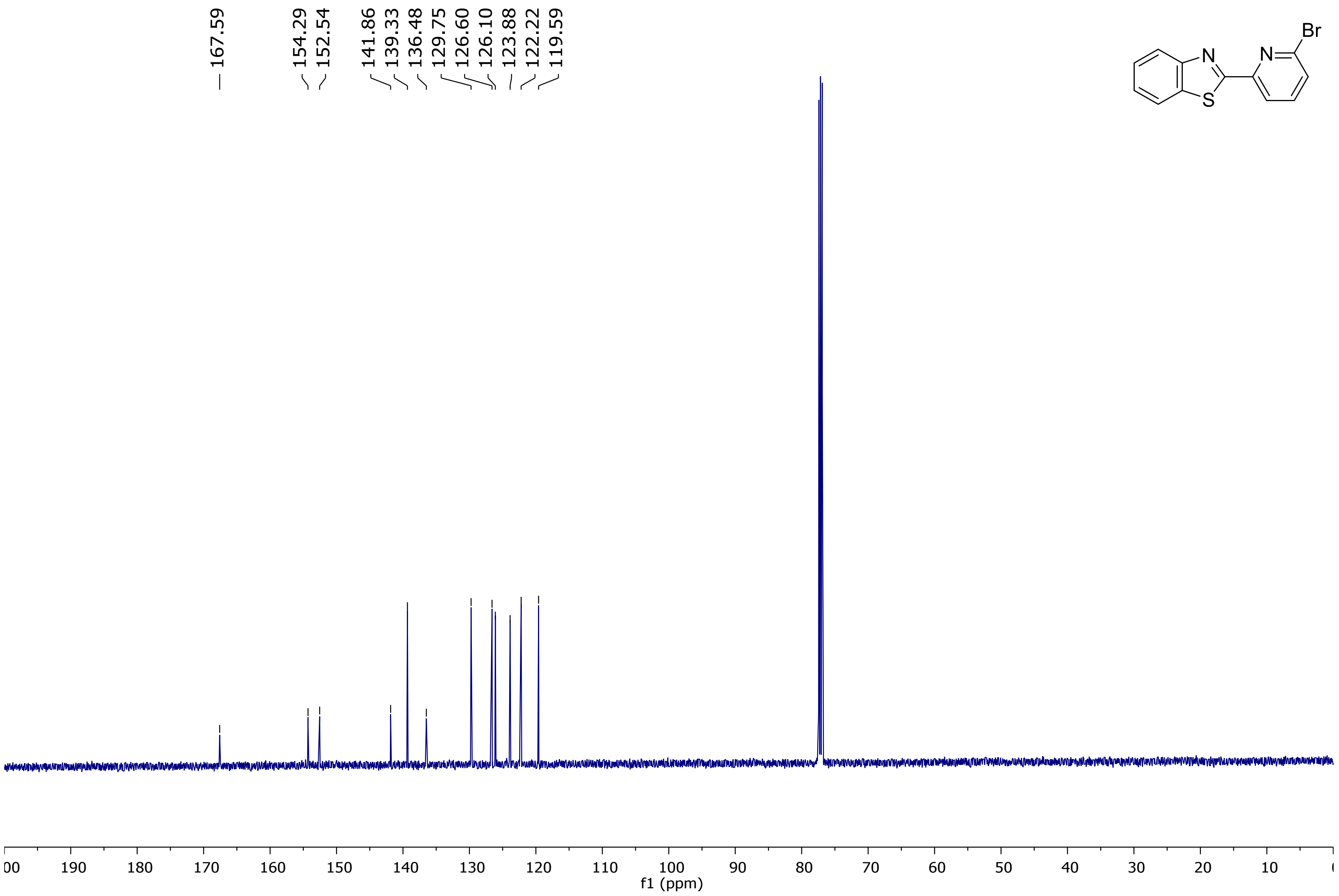

Fig. S8. ^{13}C NMR spectrum of $\mathbf{L_3}$ in $CDCl_3$ at RT

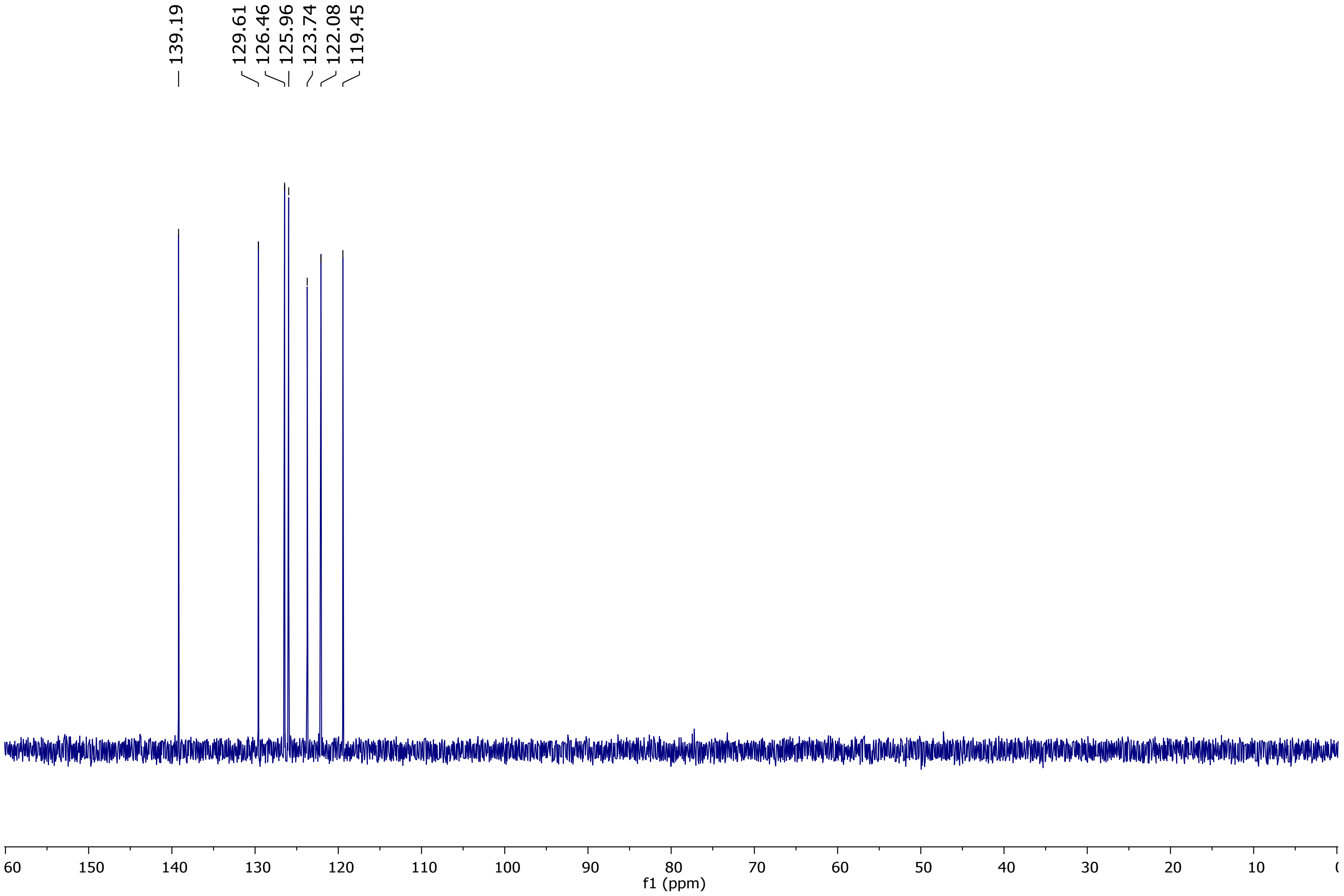

Figure S9. ^{13}C DEPT NMR spectrum of $\mathbf{L_3}$ in $CDCl_3$ at RT

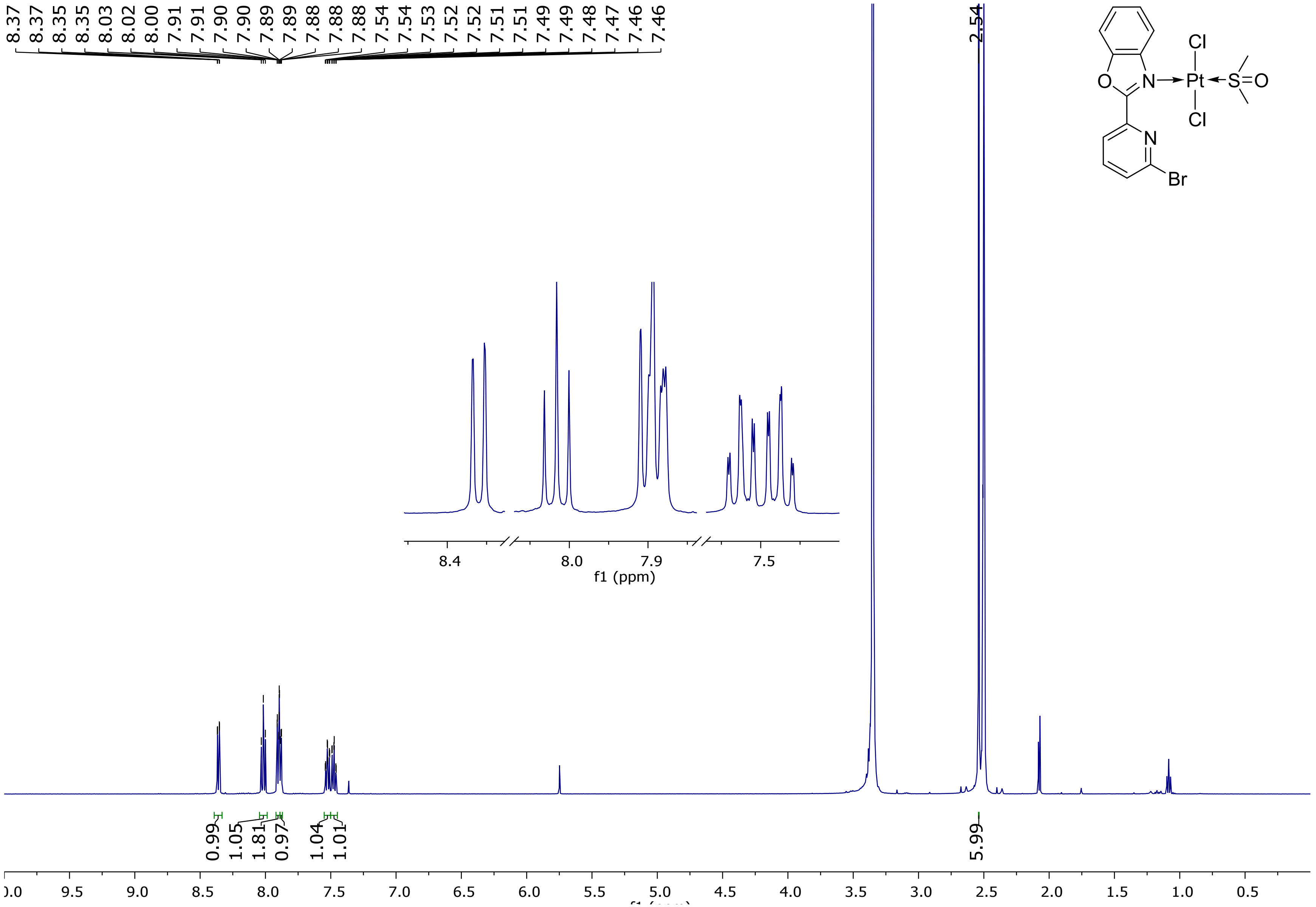

Figure S10. ^{13}C NMR spectrum of **2** in DMSO-d_6 at RT

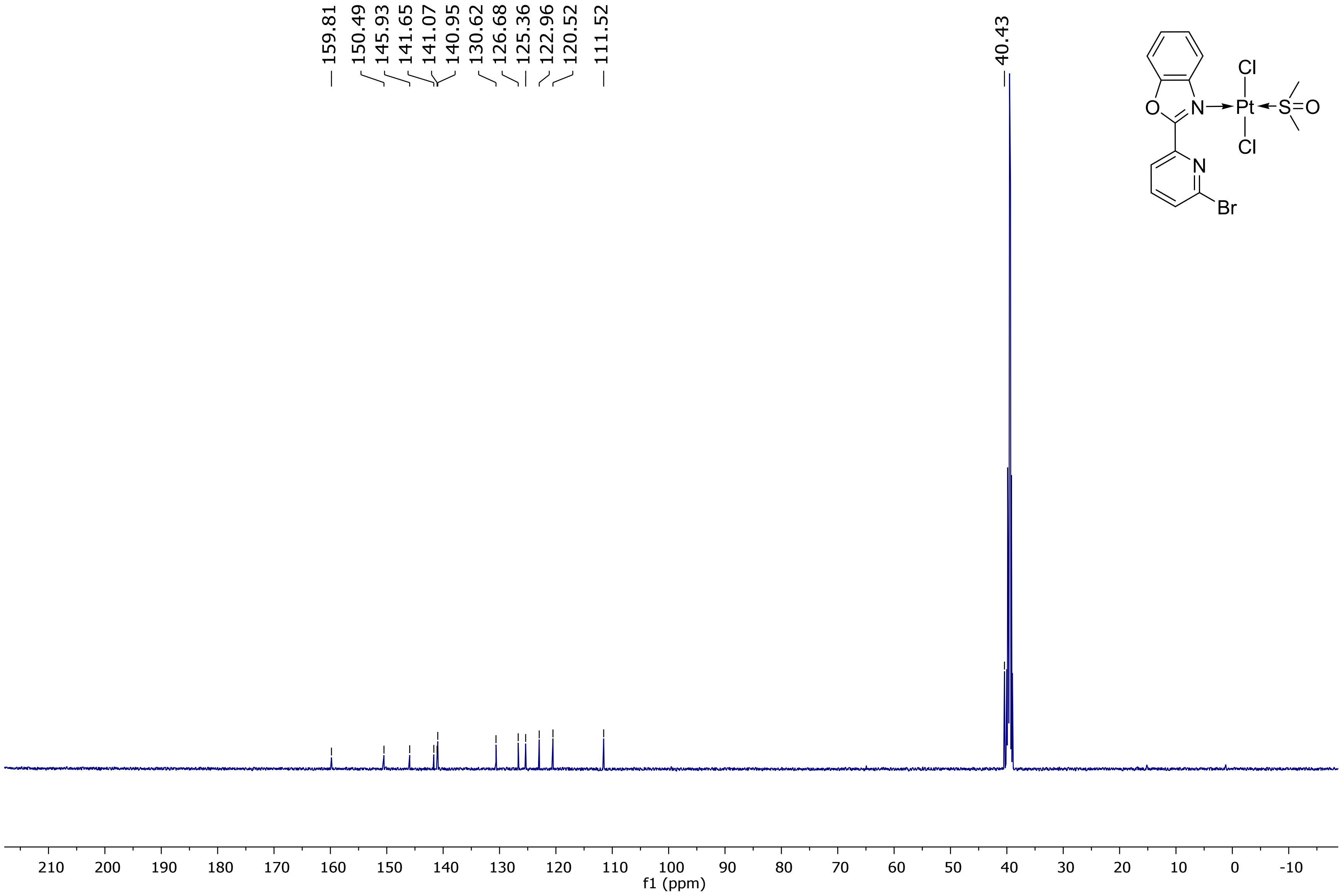

Fig. S11. ^{13}C NMR spectrum of **2** in DMSO-d_6 at RT

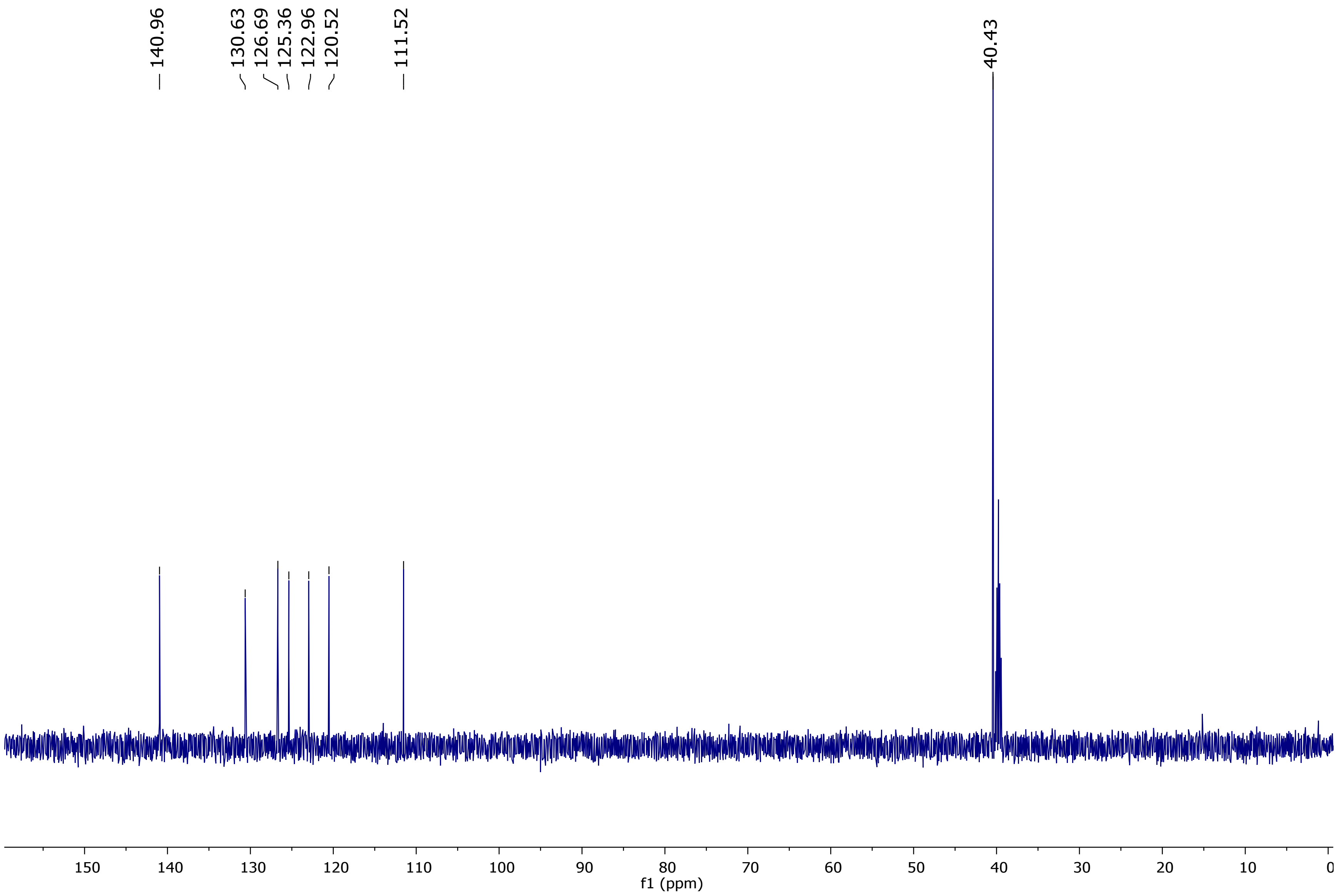

Fig. S12. ^{13}C DEPT NMR spectrum of **2** in DMSO-d_6 at RT

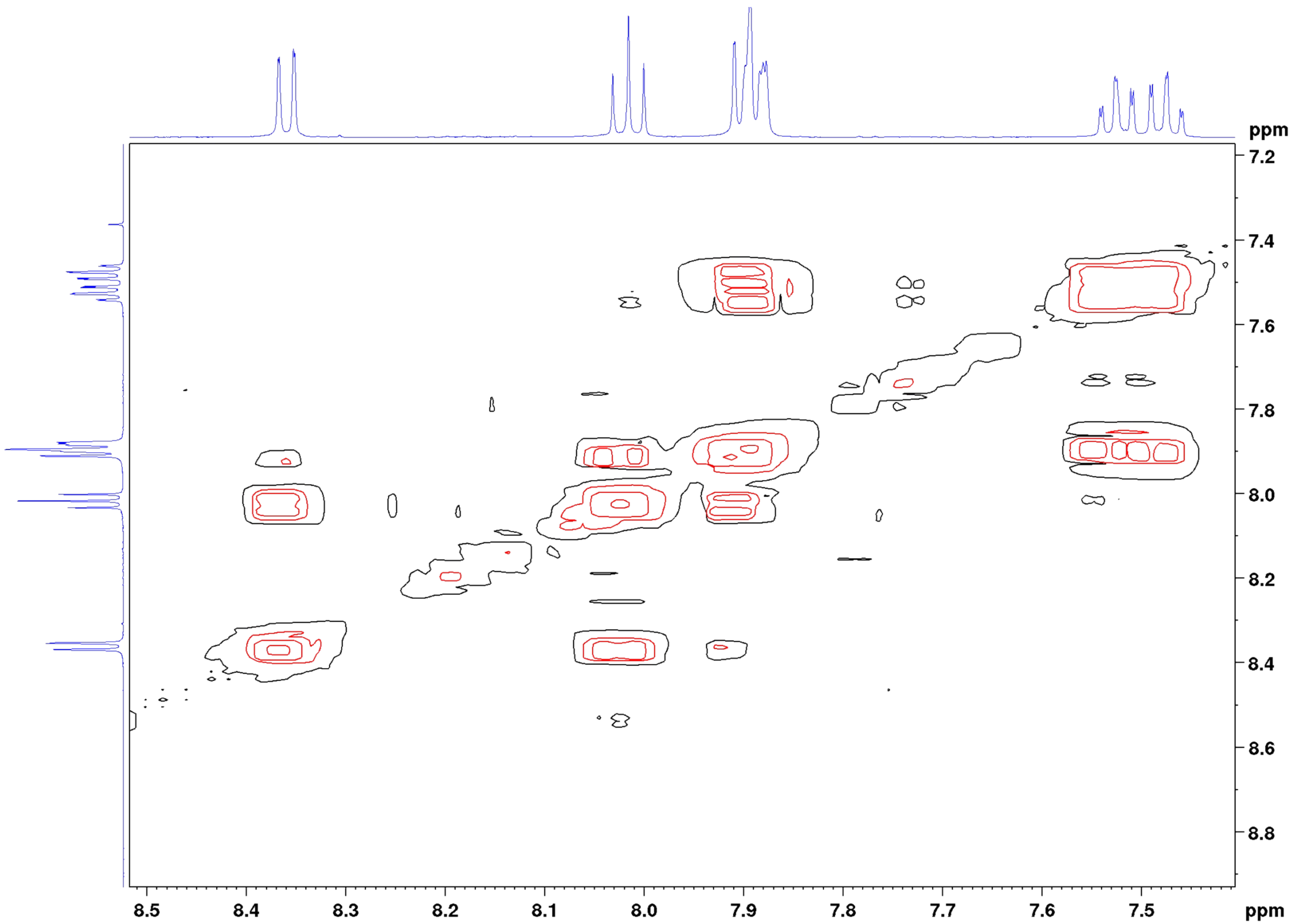

Fig. S13. ^{1}H COSY NMR spectrum of **2** in DMSO-d_6 at RT

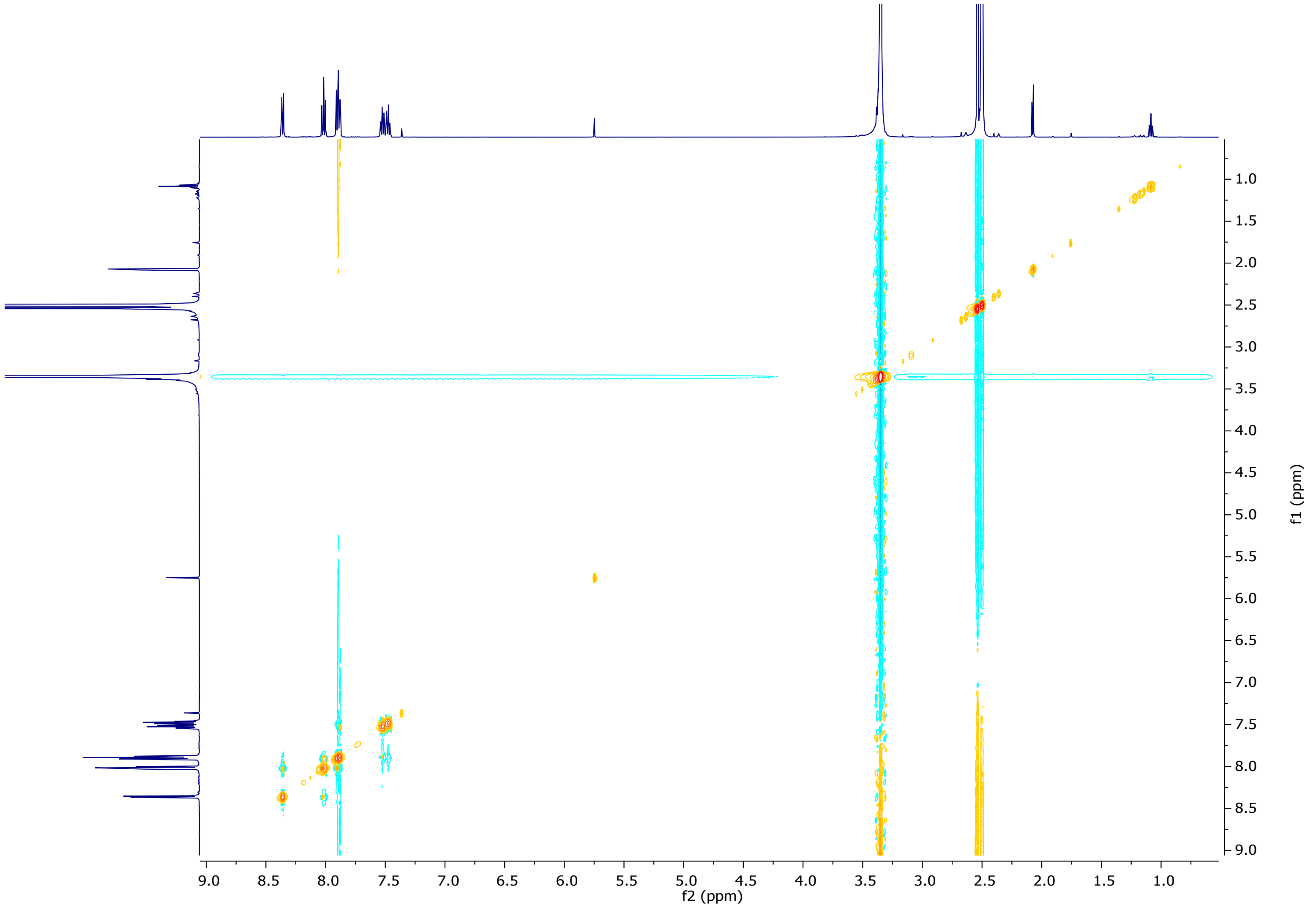

Fig. S14. ^{1}H-^{1}H NOESY NMR spectrum of **2** in DMSO-d_6 at RT

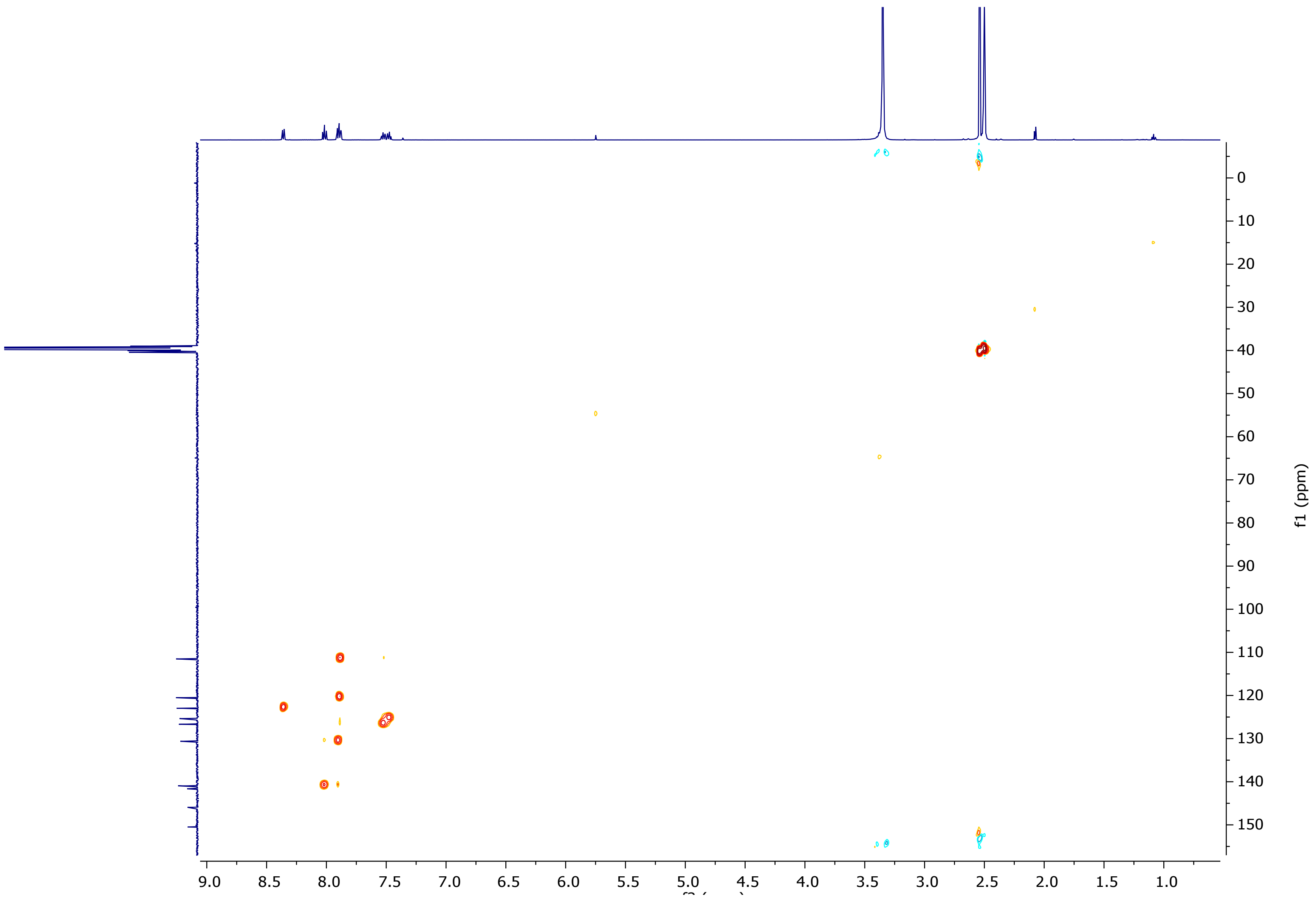

Fig. S15. ^{1}H-^{13}C HSQC NMR spectrum of **2** in DMSO-d_6 at RT

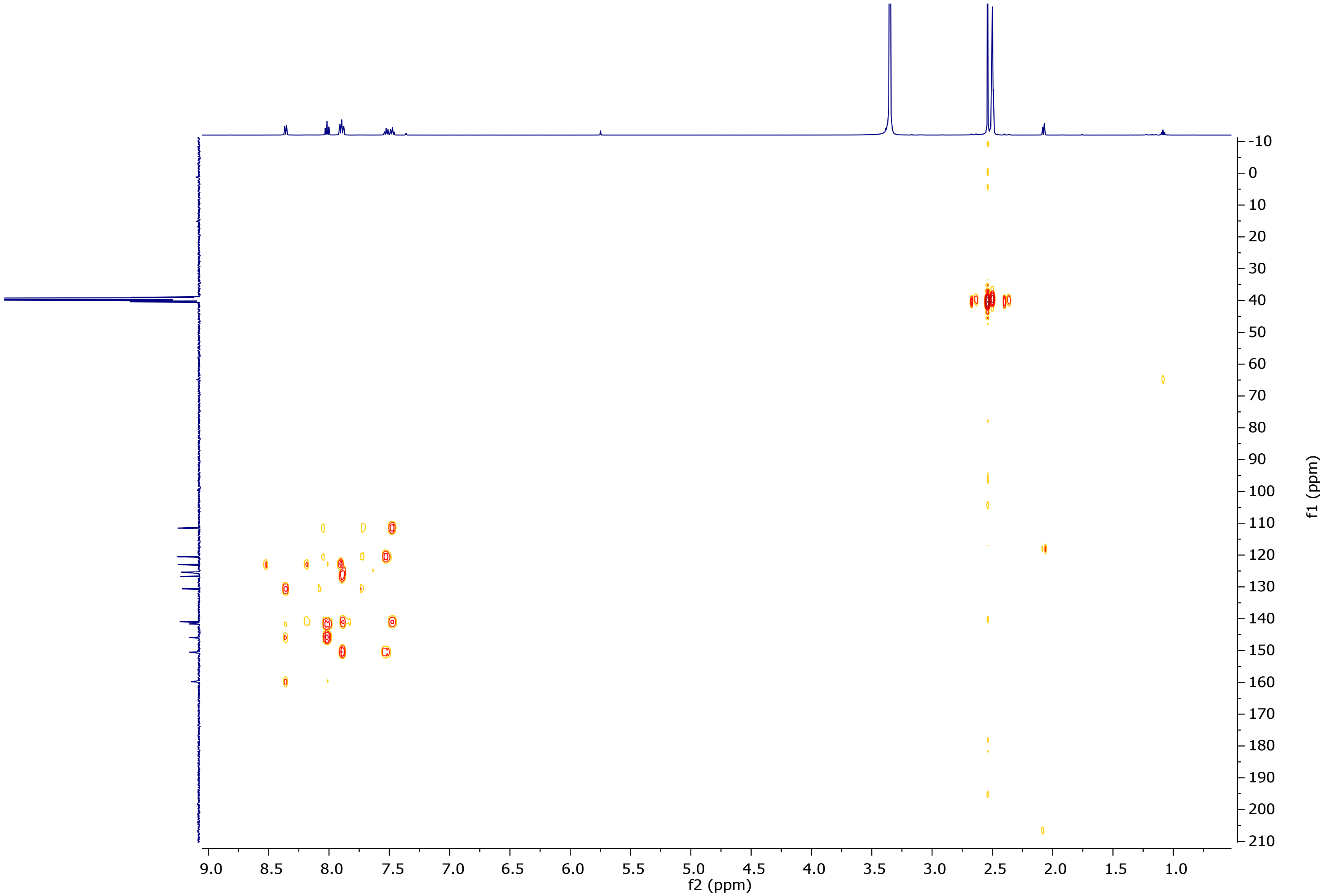

Fig. S16. ^{1}H-^{13}C HMBC NMR spectrum of **2** in DMSO-d_6 at RT

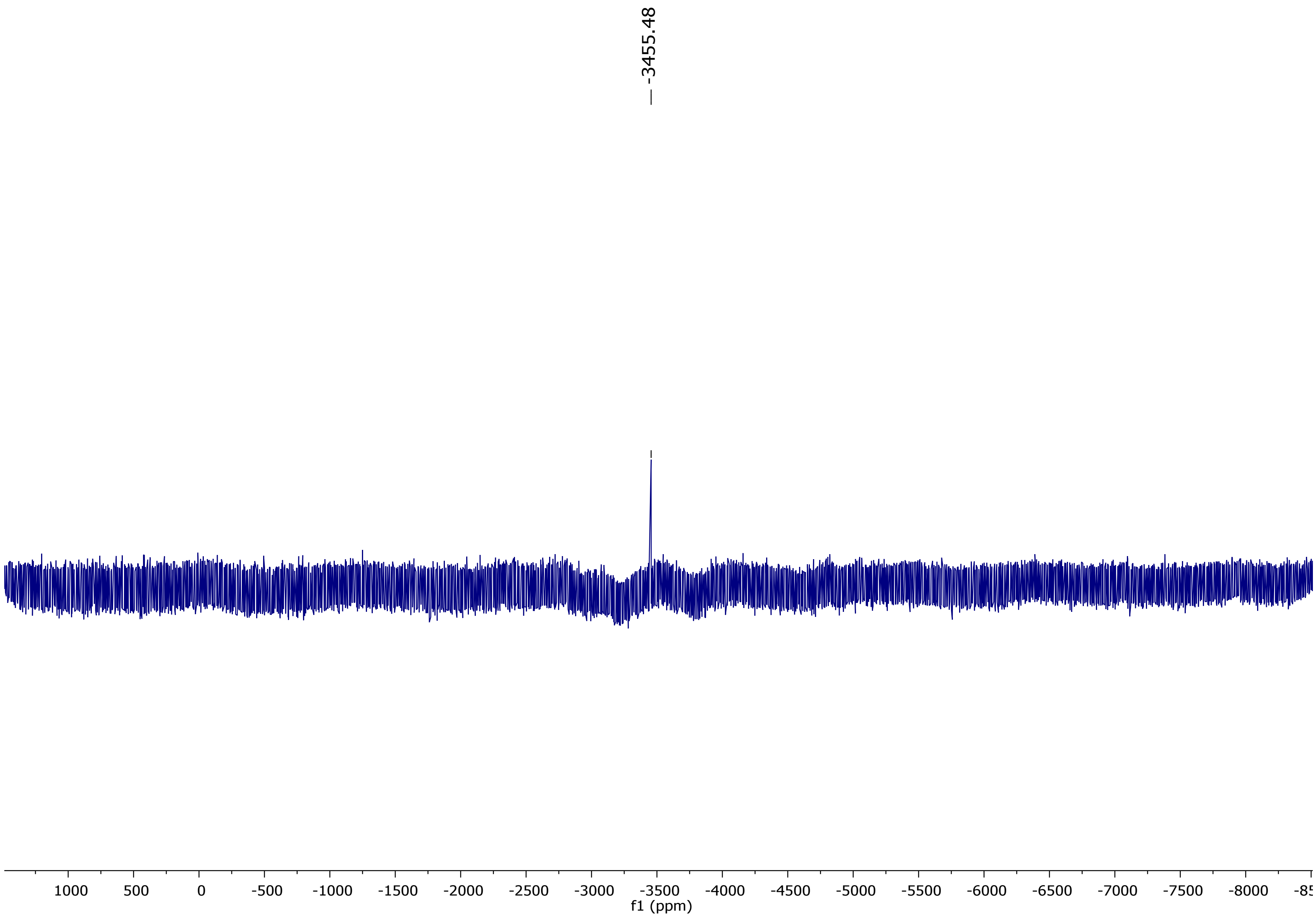

Fig. S17. ^{195}Pt NMR spectrum of **2** in DMSO-d_6 at RT

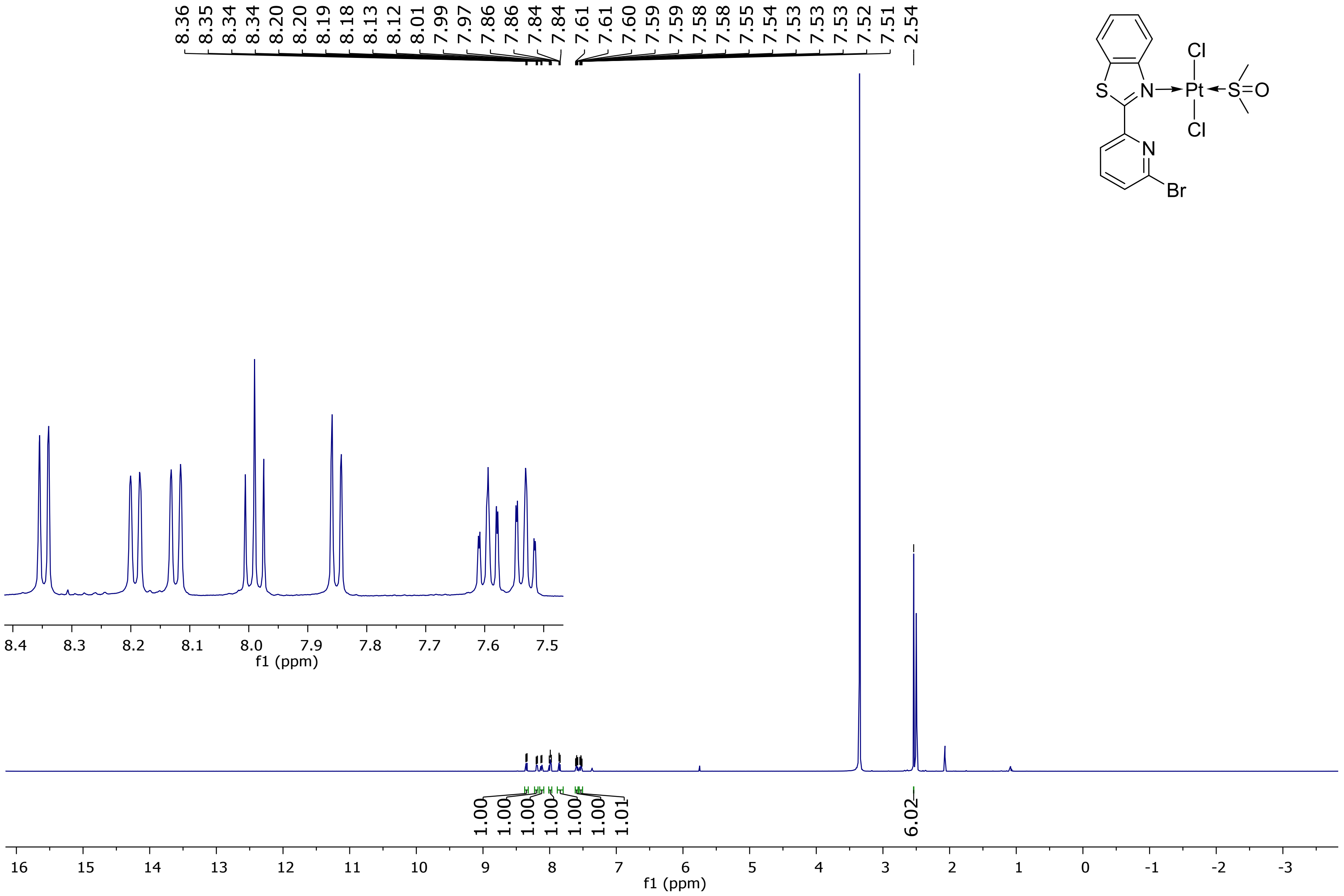

Fig. S18. ^{1}H NMR spectrum of **3** in DMSO-d_6 at RT

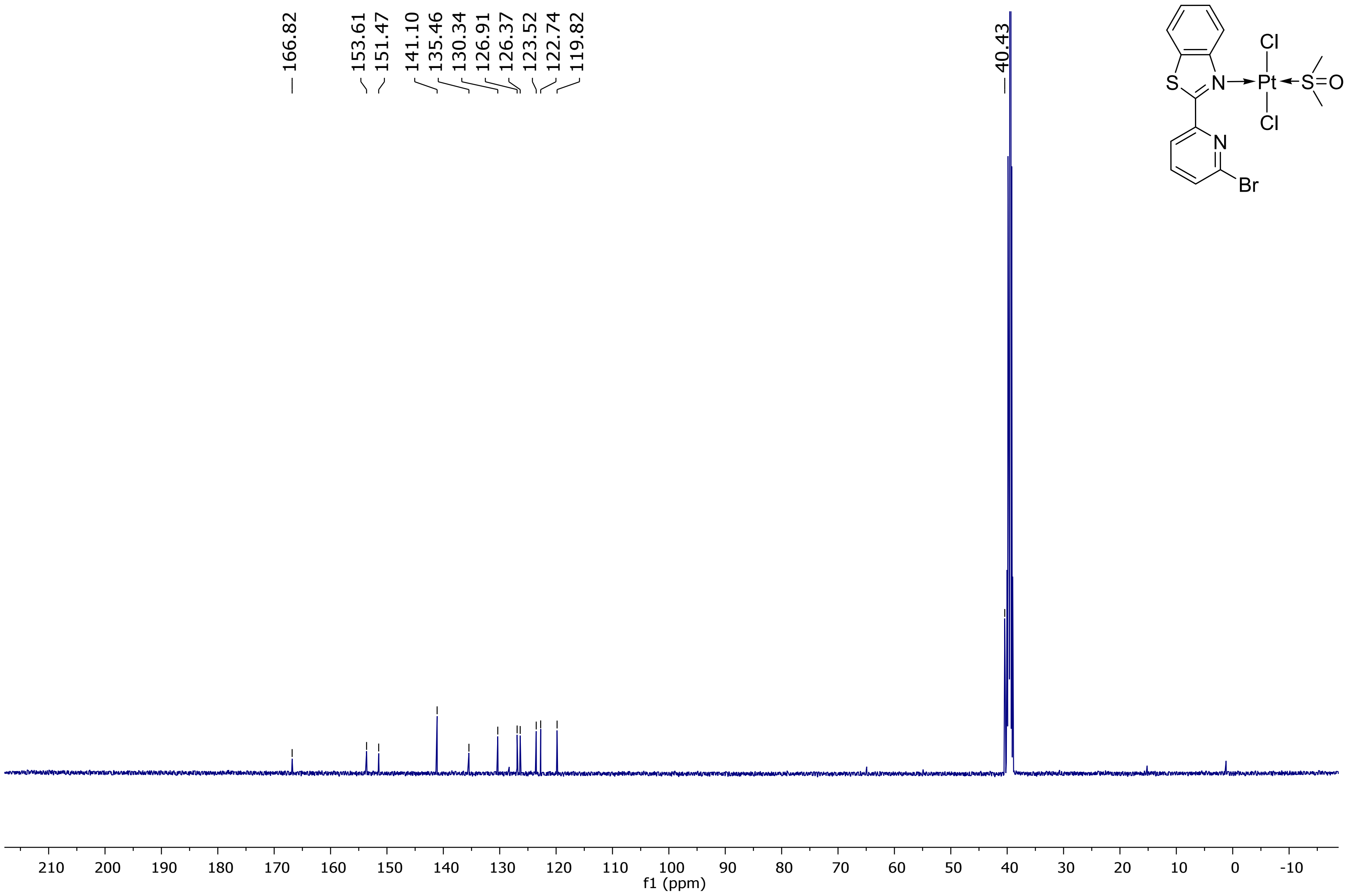

Fig. S19. ^{13}C HMBC NMR spectrum of **3** in DMSO-d_6 at RT

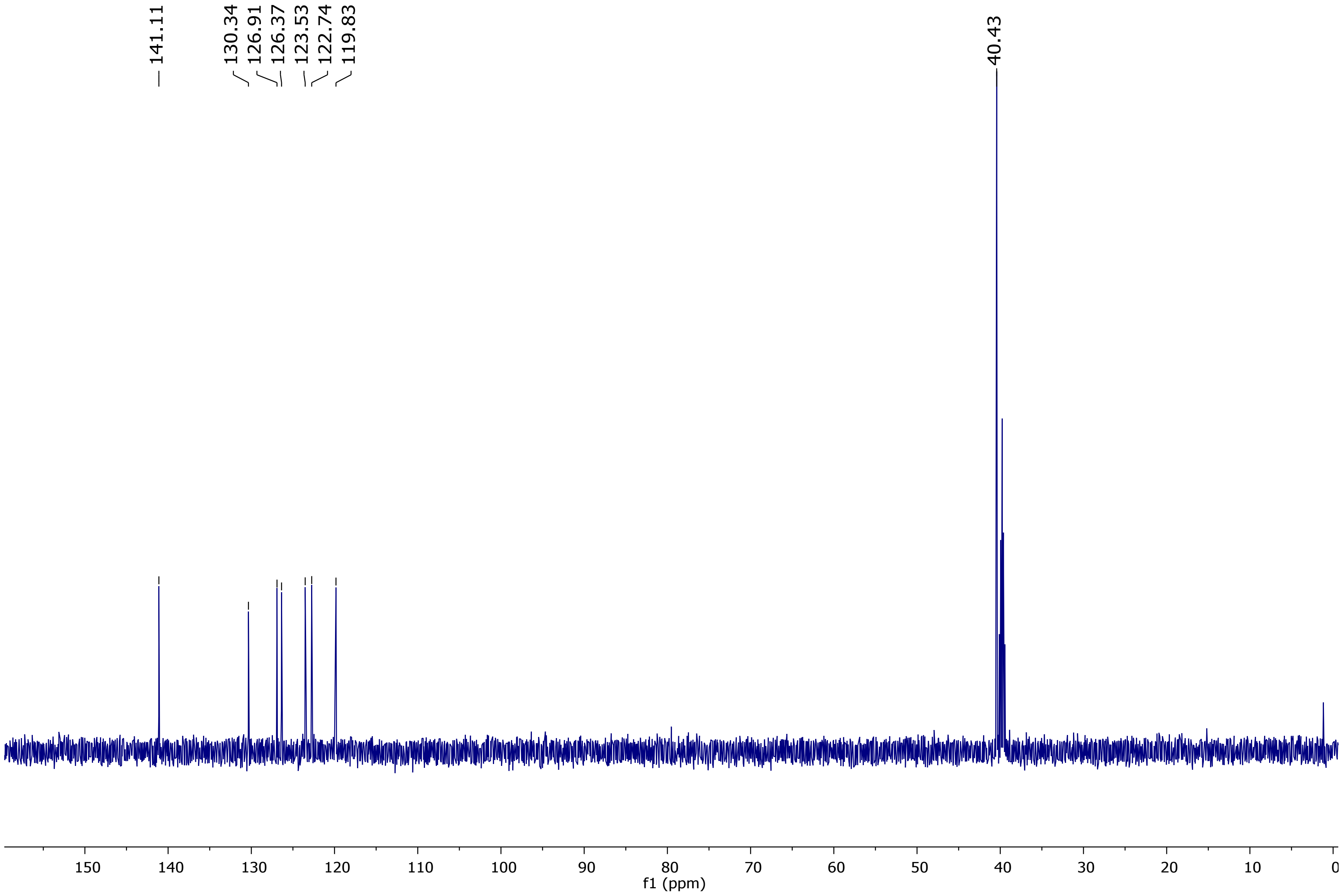

Fig. S20. ^{13}C DEPT NMR spectrum of **3** in DMSO-d_6 at RT

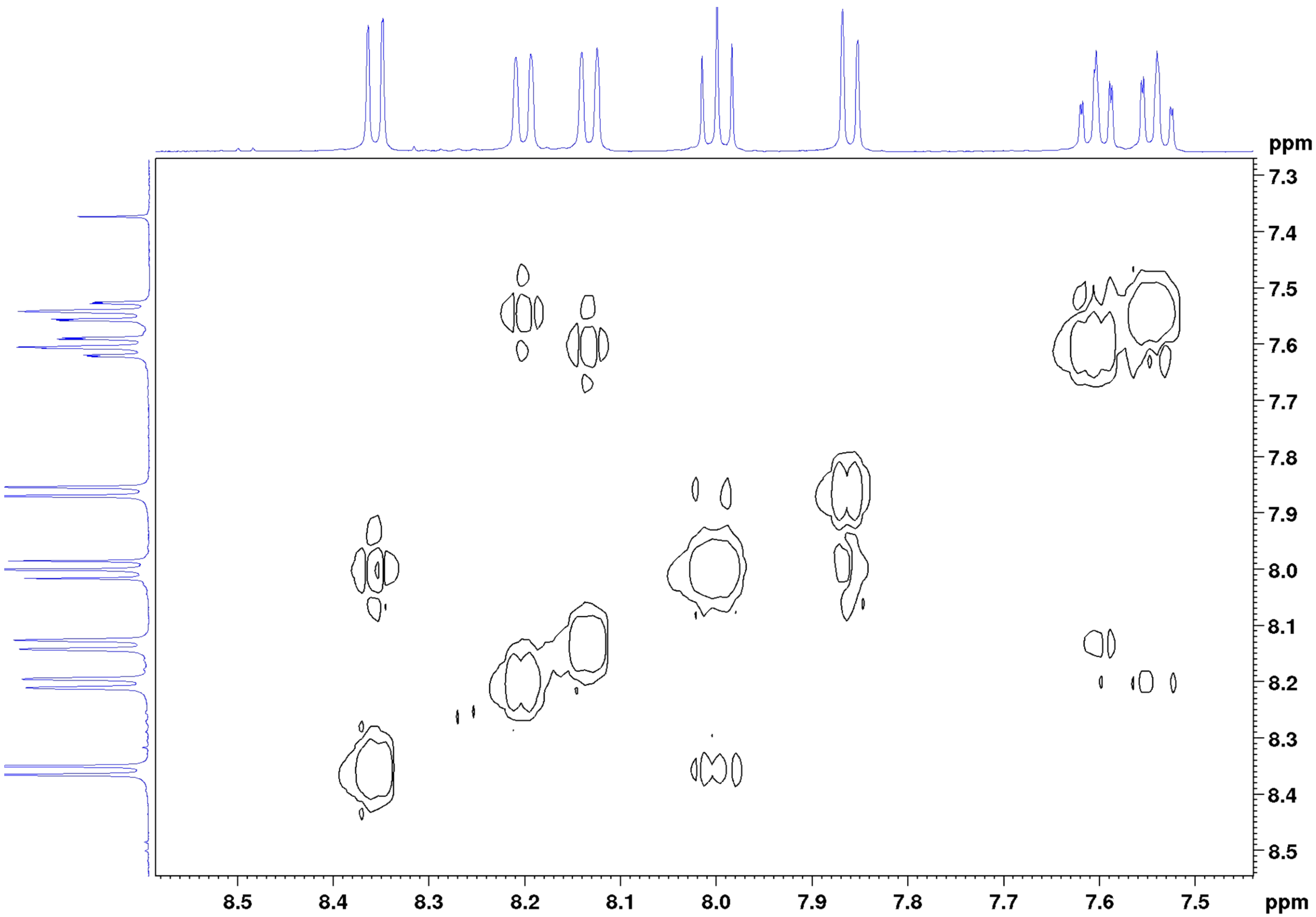

Fig. S21. ^{1}H-^{1}H COSY NMR spectrum of **3** in DMSO-d_6 at RT

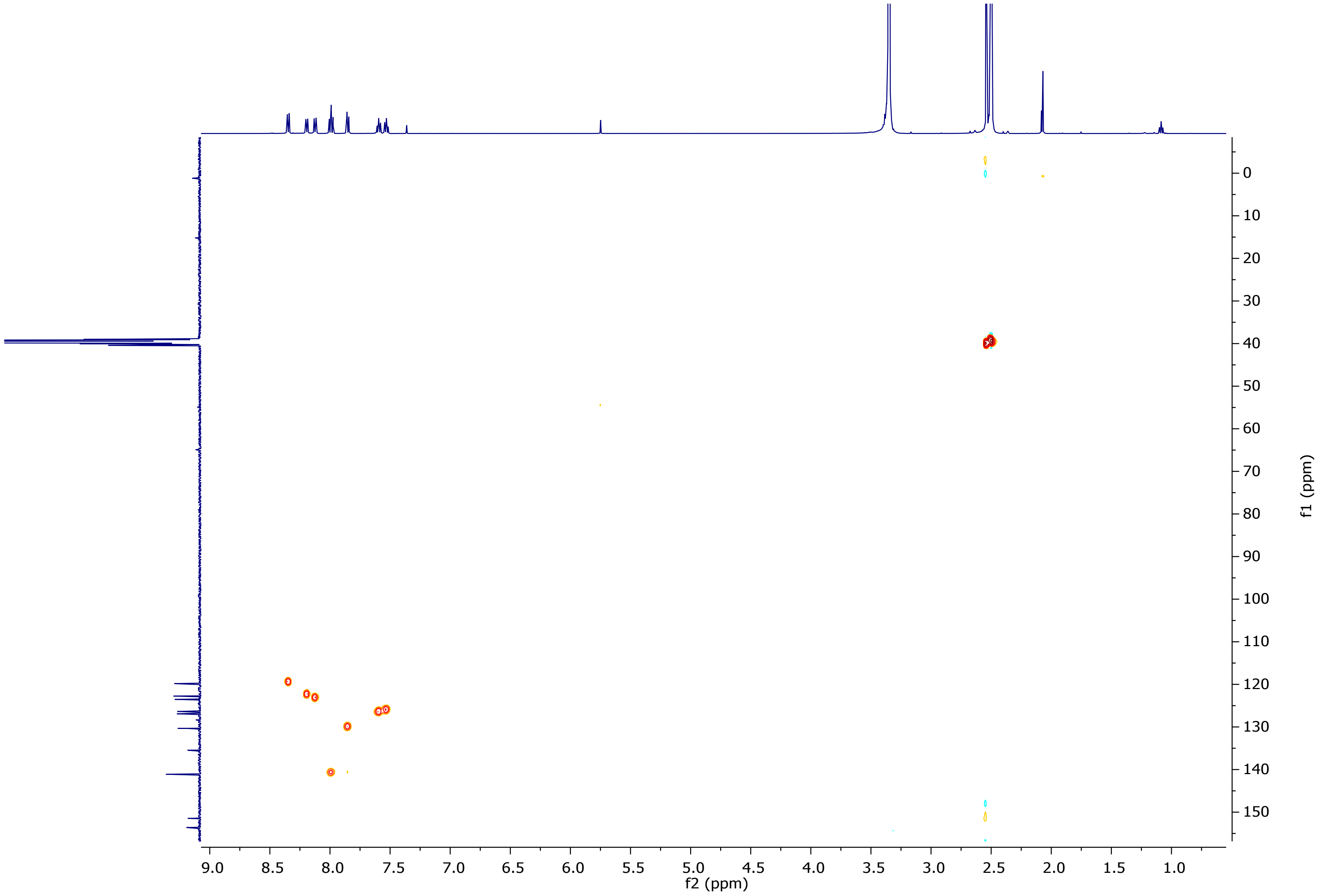

Fig. S22. ^{1}H-^{13}C HSQC NMR spectrum of **3** in DMSO-d_6 at RT

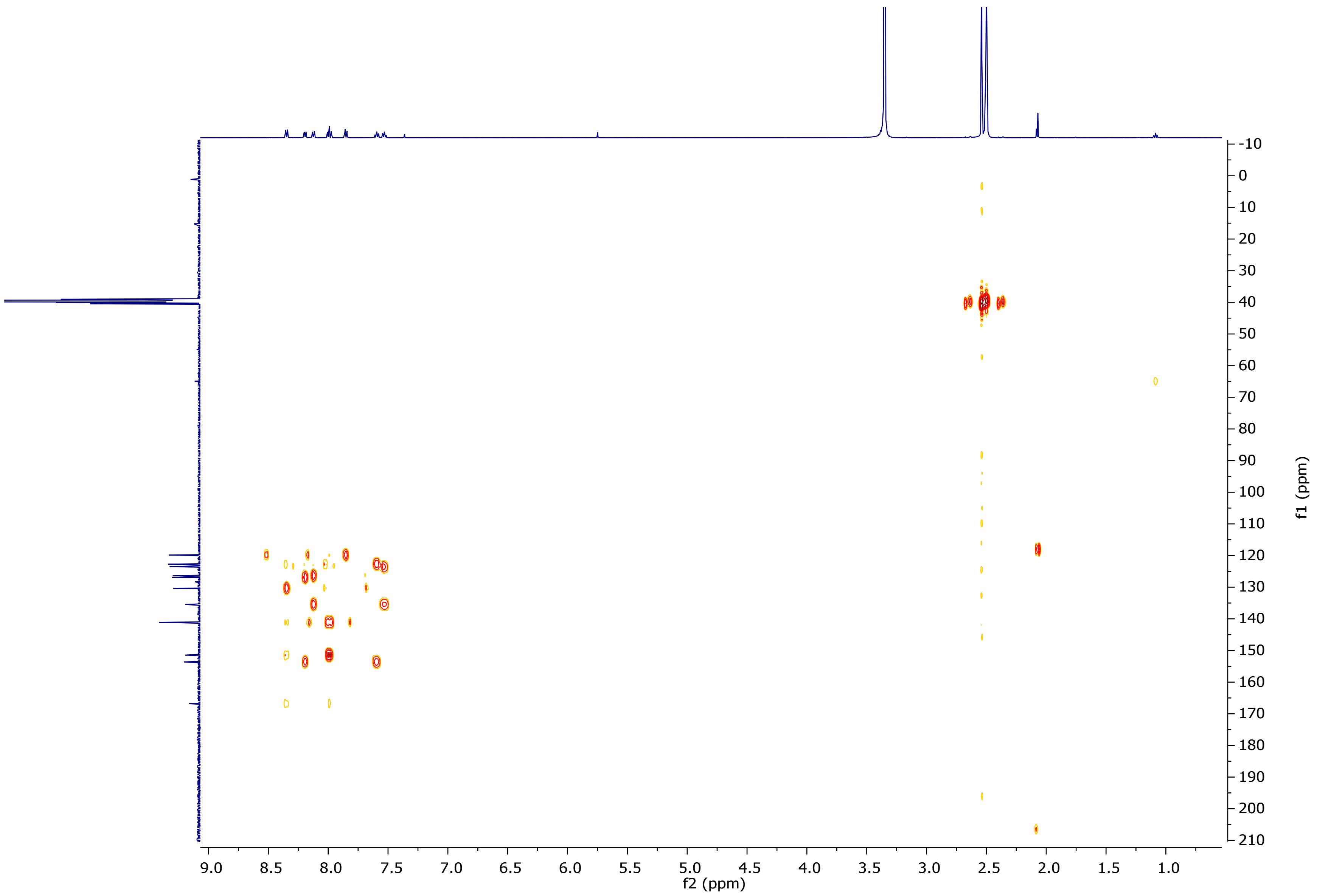

Fig. S23. ^1H-^{13}C HMBC NMR spectrum of **3** in DMSO-d_6 at RT

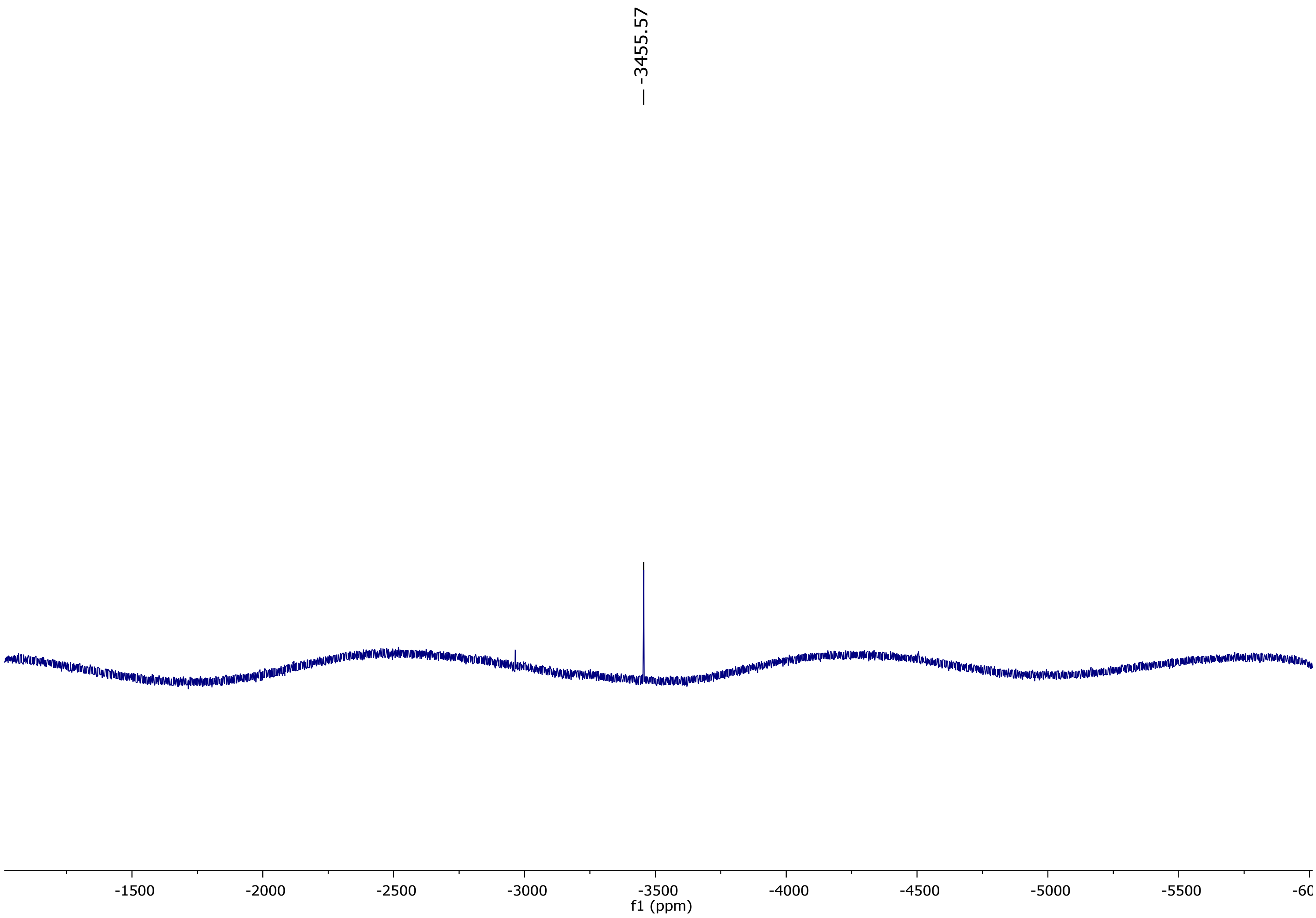

Fig. S24. ^{195}Pt NMR spectrum of **3** in DMSO-d_6 at RT

Raman and FTIR spectra

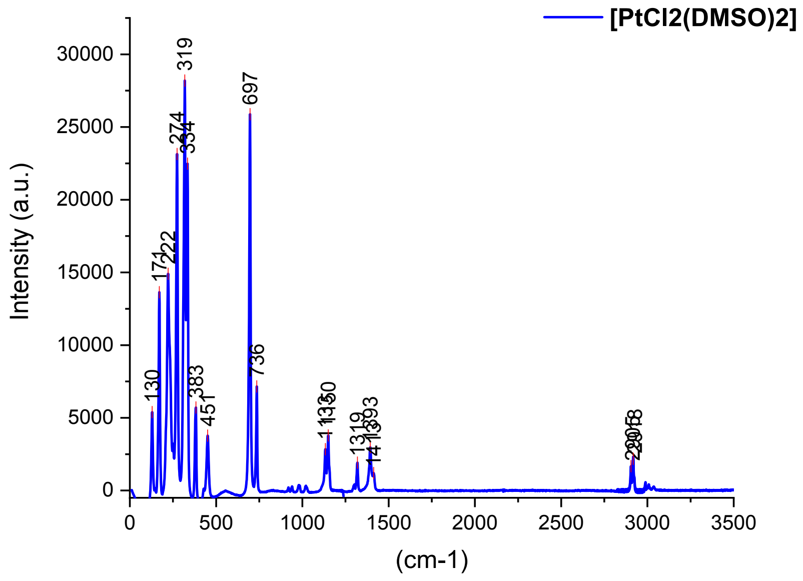

Fig. S25. Raman spectrum of $[Pt(Cl)_2(DMSO)_2]$.

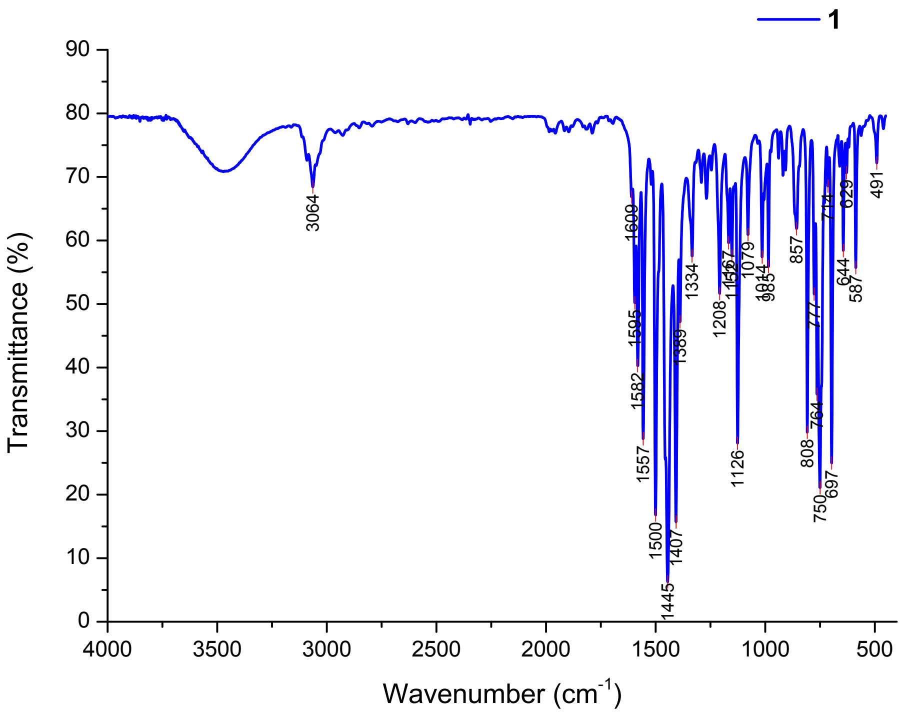

Fig. S26. IR spectrum (KBr) of complex **1**.

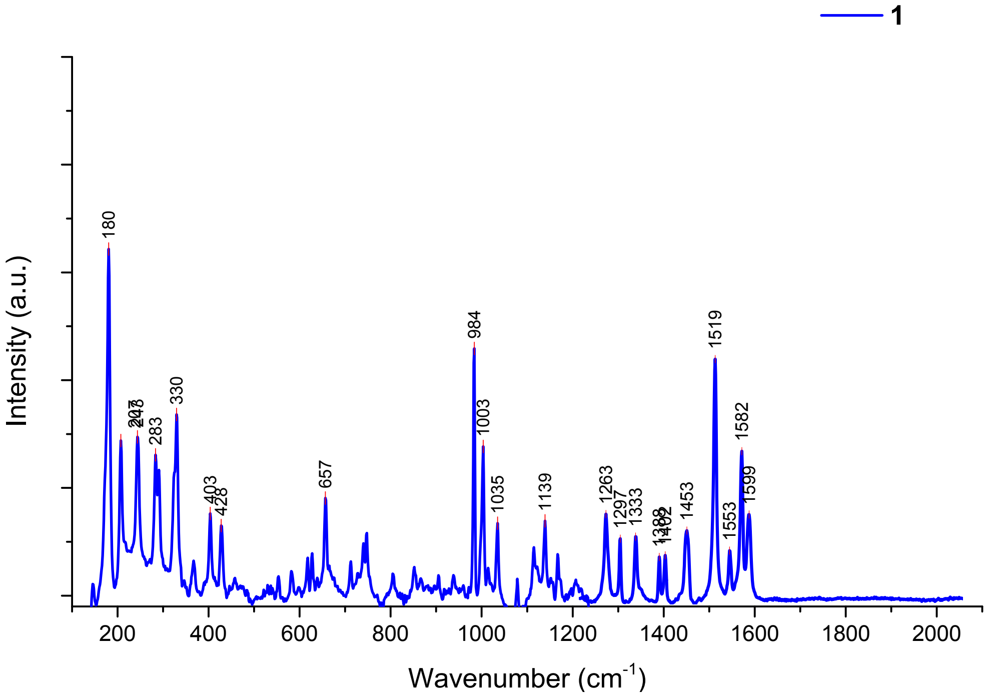

Fig. S27. Raman spectrum of complex **1.**

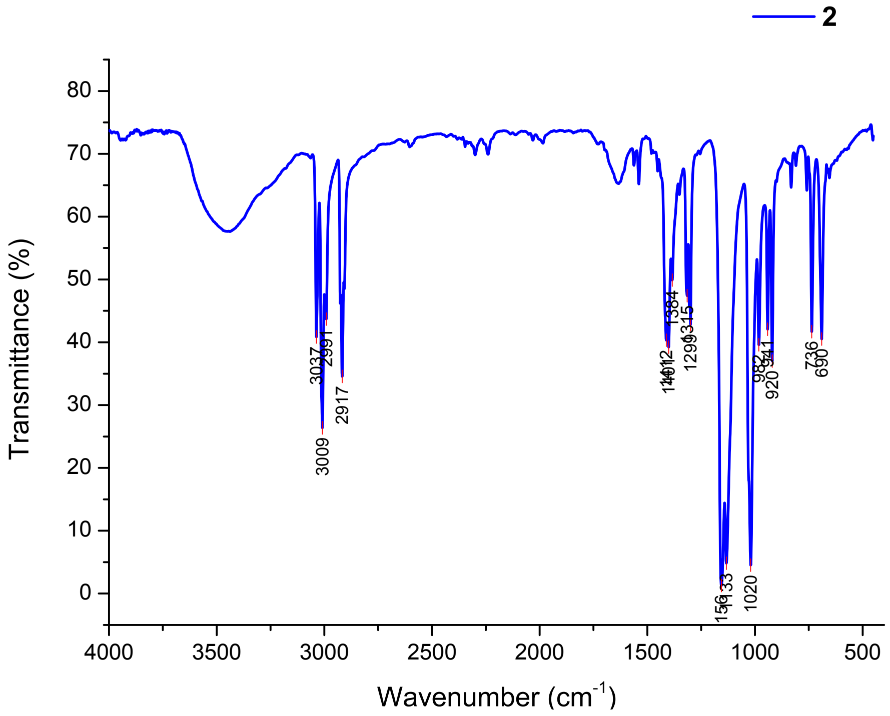

Fig. S28. IR spectrum (KBr) of complex **2**.

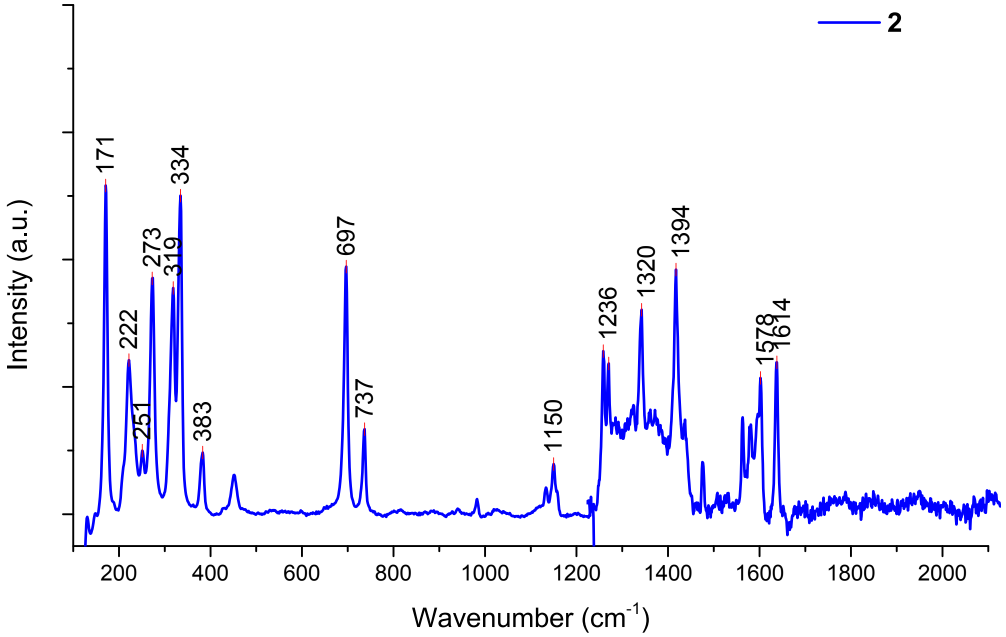

Fig. S29. Raman spectrum of complex **2**.

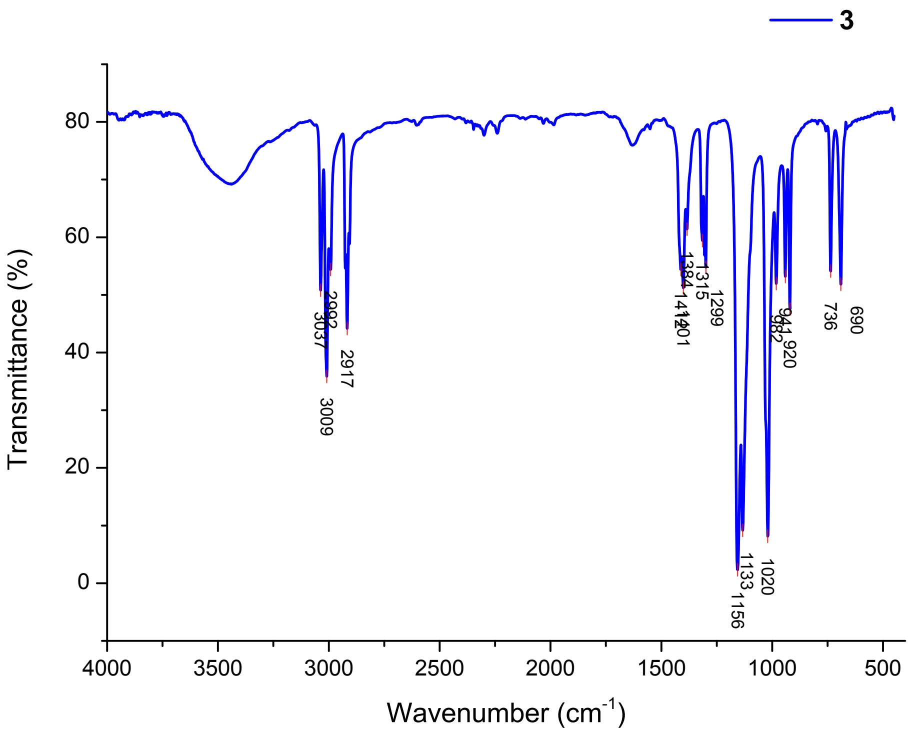

Fig. S30. IR spectrum (KBr) of complex **3**.

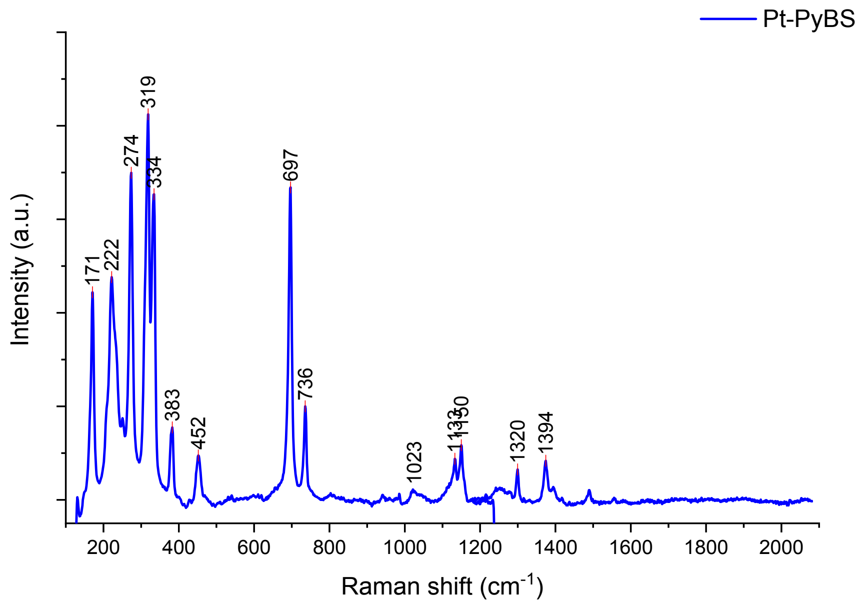

Fig. S31. Raman spectrum of complex **3**.

Crystal data and structure refinement for complex 2.

Identification code	shelx	
Empirical formula	$C_{14} H_{13}BrCl_2N_2O_2PtS$	
Formula weight	619.22	
Temperature	130(2) K	
Wavelength	0.71073 Å	
Crystal system	Triclinic	
Space group	P -1	
Unit cell dimensions	a = 8.4296(7) Å	α = 94.288(7)°.
	b = 8.4438(7) Å	β = 107.426(8)°.
	c = 13.7685(12) Å	γ = 107.058(7)°.
Volume	879.58(14) Å^3	
Z	2	
Density (calculated)	2.338 Mg/m^3	
Absorption coefficient	10.678 mm^{-1}	
F(000)	580	
Crystal size	0.380 x 0.170 x 0.080 mm^3	
Theta range for data collection	3.382 to 29.388°.	
Index ranges	-11<=h<=10, -9<=k<=11, -18<=l<=18	
Reflections collected	8147	
Independent reflections	4127 [R(int) = 0.0326]	
Completeness to theta = 25.242°	99.7 %	
Refinement method	Full-matrix least-squares on F^2	
Data / restraints / parameters	4127 / 0 / 210	
Goodness-of-fit on F^2	1.055	
Final R indices [I>2sigma(I)]	R1 = 0.0300, wR2 = 0.0531	
R indices (all data)	R1 = 0.0376, wR2 = 0.0571	
Extinction coefficient	n/a	
Largest diff. peak and hole	1.603 and -1.731 e.Å^{-3}	

Table S3. Atomic coordinates (x 10^4) and equivalent isotropic displacement parameters ($Å^2$x 10^3) for complex **2**. U(eq) is defined as one third of the trace of the orthogonalized U^{ij} tensor.

	x	y	z	U(eq)
C(1)	6400(6)	871(7)	3755(5)	35(2)
C(2)	5416(7)	2938(6)	4882(4)	32(1)
C(3)	4097(6)	5518(5)	1048(4)	17(1)
C(4)	1777(6)	4598(6)	-329(4)	19(1)
C(5)	1640(6)	3476(5)	351(4)	18(1)
C(6)	145(6)	2058(6)	95(4)	22(1)
C(7)	-1132(6)	1828(6)	-852(4)	27(1)
C(8)	-948(6)	2986(6)	-1532(4)	24(1)
C(9)	523(6)	4409(6)	-1275(4)	22(1)
C(10)	5787(6)	6721(5)	1703(4)	18(1)
C(11)	8049(6)	7392(6)	3209(4)	27(1)
C(12)	8970(6)	8891(6)	2965(5)	27(1)
C(13)	8174(6)	9259(6)	2026(5)	25(1)
C(14)	6569(6)	8163(6)	1364(4)	24(1)
Br(1)	9046(1)	6838(1)	4511(1)	72(1)
Cl(1)	5553(2)	2014(2)	1600(1)	29(1)
Cl(2)	1919(2)	3585(2)	3095(1)	28(1)
N(1)	3157(4)	4088(4)	1220(3)	16(1)
N(2)	6512(5)	6346(5)	2625(3)	22(1)
O(1)	3197(4)	21(4)	3788(3)	28(1)
O(2)	3346(4)	5894(4)	127(3)	19(1)
Pt(1)	3839(1)	2841(1)	2417(1)	17(1)
S(1)	4560(1)	1502(1)	3714(1)	19(1)

Table S4. Bond lengths [Å] and angles [°] for complex **2**.

C(1)-S(1)	1.769(5)
C(1)-H(1A)	0.9800
C(1)-H(1B)	0.9800
C(1)-H(1C)	0.9800
C(2)-S(1)	1.755(6)
C(2)-H(2A)	0.9800
C(2)-H(2B)	0.9800
C(2)-H(2C)	0.9800
C(3)-N(1)	1.317(5)
C(3)-O(2)	1.345(5)
C(3)-C(10)	1.456(7)
C(4)-C(9)	1.373(7)
C(4)-O(2)	1.375(5)
C(4)-C(5)	1.386(6)
C(5)-C(6)	1.390(6)
C(5)-N(1)	1.391(6)
C(6)-C(7)	1.377(7)
C(6)-H(6)	0.9500
C(7)-C(8)	1.410(6)
C(7)-H(7)	0.9500
C(8)-C(9)	1.381(7)
C(8)-H(8)	0.9500
C(9)-H(9)	0.9500
C(10)-N(2)	1.339(6)
C(10)-C(14)	1.391(5)
C(11)-N(2)	1.305(6)
C(11)-C(12)	1.401(6)
C(11)-Br(1)	1.895(5)
C(12)-C(13)	1.371(7)
C(12)-H(12)	0.9500
C(13)-C(14)	1.385(7)
C(13)-H(13)	0.9500
C(14)-H(14)	0.9500
Cl(1)-Pt(1)	2.2990(12)

Cl(2)-Pt(1)	2.3022(12)
N(1)-Pt(1)	2.051(3)
O(1)-S(1)	1.463(3)
Pt(1)-S(1)	2.2108(11)
S(1)-C(1)-H(1A)	109.5
S(1)-C(1)-H(1B)	109.5
H(1A)-C(1)-H(1B)	109.5
S(1)-C(1)-H(1C)	109.5
H(1A)-C(1)-H(1C)	109.5
H(1B)-C(1)-H(1C)	109.5
S(1)-C(2)-H(2A)	109.5
S(1)-C(2)-H(2B)	109.5
H(2A)-C(2)-H(2B)	109.5
S(1)-C(2)-H(2C)	109.5
H(2A)-C(2)-H(2C)	109.5
H(2B)-C(2)-H(2C)	109.5
N(1)-C(3)-O(2)	113.5(4)
N(1)-C(3)-C(10)	129.7(4)
O(2)-C(3)-C(10)	116.7(3)
C(9)-C(4)-O(2)	127.9(4)
C(9)-C(4)-C(5)	124.4(4)
O(2)-C(4)-C(5)	107.7(4)
C(4)-C(5)-C(6)	119.9(5)
C(4)-C(5)-N(1)	107.7(4)
C(6)-C(5)-N(1)	132.4(4)
C(7)-C(6)-C(5)	117.1(4)
C(7)-C(6)-H(6)	121.5
C(5)-C(6)-H(6)	121.5
C(6)-C(7)-C(8)	121.8(4)
C(6)-C(7)-H(7)	119.1
C(8)-C(7)-H(7)	119.1
C(9)-C(8)-C(7)	121.4(5)
C(9)-C(8)-H(8)	119.3
C(7)-C(8)-H(8)	119.3
C(4)-C(9)-C(8)	115.5(4)
C(4)-C(9)-H(9)	122.2

C(8)-C(9)-H(9)	122.2
N(2)-C(10)-C(14)	123.4(5)
N(2)-C(10)-C(3)	116.2(4)
C(14)-C(10)-C(3)	120.4(4)
N(2)-C(11)-C(12)	125.4(5)
N(2)-C(11)-Br(1)	116.2(3)
C(12)-C(11)-Br(1)	118.4(4)
C(13)-C(12)-C(11)	116.2(5)
C(13)-C(12)-H(12)	121.9
C(11)-C(12)-H(12)	121.9
C(12)-C(13)-C(14)	120.6(4)
C(12)-C(13)-H(13)	119.7
C(14)-C(13)-H(13)	119.7
C(13)-C(14)-C(10)	117.5(5)
C(13)-C(14)-H(14)	121.3
C(10)-C(14)-H(14)	121.3
C(3)-N(1)-C(5)	105.5(4)
C(3)-N(1)-Pt(1)	128.5(3)
C(5)-N(1)-Pt(1)	125.7(3)
C(11)-N(2)-C(10)	116.9(4)
C(3)-O(2)-C(4)	105.5(3)
N(1)-Pt(1)-S(1)	179.52(10)
N(1)-Pt(1)-Cl(1)	87.00(10)
S(1)-Pt(1)-Cl(1)	93.47(4)
N(1)-Pt(1)-Cl(2)	89.68(10)
S(1)-Pt(1)-Cl(2)	89.84(4)
Cl(1)-Pt(1)-Cl(2)	174.85(5)
O(1)-S(1)-C(2)	109.0(2)
O(1)-S(1)-C(1)	106.6(2)
C(2)-S(1)-C(1)	100.7(3)
O(1)-S(1)-Pt(1)	117.86(14)
C(2)-S(1)-Pt(1)	108.92(16)
C(1)-S(1)-Pt(1)	112.43(18)

Symmetry transformations used to generate equivalent atoms:

Table S5. Anisotropic displacement parameters ($Å^2 x 10^3$)for ncj-nd-029. The anisotropic displacement factor exponent takes the form: $-2p^2[h^2a^{*2}U^{11} + ... + 2 h k a^* b^* U^{12}]$

	U^{11}	U^{22}	U^{33}	U^{23}	U^{13}	U^{12}
C(1)	32(3)	52(4)	33(4)	23(3)	14(3)	24(3)
C(2)	50(3)	19(3)	15(3)	0(2)	-3(3)	7(2)
C(3)	25(2)	18(2)	13(3)	6(2)	9(2)	9(2)
C(4)	21(2)	19(2)	20(3)	2(2)	9(2)	10(2)
C(5)	26(2)	17(2)	15(3)	3(2)	7(2)	12(2)
C(6)	26(2)	18(2)	18(3)	2(2)	5(2)	6(2)
C(7)	23(2)	25(3)	25(3)	-6(2)	3(2)	5(2)
C(8)	29(3)	30(3)	13(3)	0(2)	0(2)	16(2)
C(9)	28(3)	26(3)	13(3)	4(2)	4(2)	15(2)
C(10)	18(2)	15(2)	25(3)	3(2)	8(2)	8(2)
C(11)	19(2)	31(3)	24(3)	5(3)	3(2)	2(2)
C(12)	20(2)	25(3)	34(4)	2(3)	12(2)	4(2)
C(13)	22(2)	23(3)	31(4)	6(2)	14(2)	4(2)
C(14)	26(2)	21(2)	27(3)	13(2)	11(2)	7(2)
Br(1)	41(1)	87(1)	41(1)	36(1)	-17(1)	-20(1)
Cl(1)	36(1)	39(1)	27(1)	17(1)	17(1)	23(1)
Cl(2)	34(1)	38(1)	22(1)	11(1)	13(1)	21(1)
N(1)	15(2)	21(2)	12(2)	4(2)	3(2)	7(2)
N(2)	16(2)	24(2)	20(3)	8(2)	3(2)	2(2)
O(1)	24(2)	25(2)	27(2)	12(2)	3(2)	2(1)
O(2)	21(2)	19(2)	17(2)	8(2)	3(2)	6(1)
Pt(1)	18(1)	16(1)	14(1)	5(1)	4(1)	5(1)
S(1)	19(1)	19(1)	17(1)	5(1)	4(1)	6(1)

Table S6. Torsion angles [°] for complex **2**.

C(9)-C(4)-C(5)-C(6)	-0.9(7)
O(2)-C(4)-C(5)-C(6)	178.3(4)
C(9)-C(4)-C(5)-N(1)	179.9(4)
O(2)-C(4)-C(5)-N(1)	-0.9(5)
C(4)-C(5)-C(6)-C(7)	1.3(7)
N(1)-C(5)-C(6)-C(7)	-179.8(5)
C(5)-C(6)-C(7)-C(8)	-0.8(7)
C(6)-C(7)-C(8)-C(9)	-0.2(7)
O(2)-C(4)-C(9)-C(8)	-179.0(4)
C(5)-C(4)-C(9)-C(8)	0.0(7)
C(7)-C(8)-C(9)-C(4)	0.5(7)
N(1)-C(3)-C(10)-N(2)	-0.4(7)
O(2)-C(3)-C(10)-N(2)	179.2(4)
N(1)-C(3)-C(10)-C(14)	179.3(4)
O(2)-C(3)-C(10)-C(14)	-1.1(6)
N(2)-C(11)-C(12)-C(13)	-0.6(8)
Br(1)-C(11)-C(12)-C(13)	-178.4(4)
C(11)-C(12)-C(13)-C(14)	-1.5(7)
C(12)-C(13)-C(14)-C(10)	2.0(7)
N(2)-C(10)-C(14)-C(13)	-0.5(7)
C(3)-C(10)-C(14)-C(13)	179.8(4)
O(2)-C(3)-N(1)-C(5)	-1.2(5)
C(10)-C(3)-N(1)-C(5)	178.4(4)
O(2)-C(3)-N(1)-Pt(1)	172.5(3)
C(10)-C(3)-N(1)-Pt(1)	-8.0(7)
C(4)-C(5)-N(1)-C(3)	1.2(5)
C(6)-C(5)-N(1)-C(3)	-177.8(5)
C(4)-C(5)-N(1)-Pt(1)	-172.6(3)
C(6)-C(5)-N(1)-Pt(1)	8.3(7)
C(12)-C(11)-N(2)-C(10)	2.0(8)
Br(1)-C(11)-N(2)-C(10)	179.8(3)
C(14)-C(10)-N(2)-C(11)	-1.4(7)
C(3)-C(10)-N(2)-C(11)	178.3(4)
N(1)-C(3)-O(2)-C(4)	0.6(5)

C(10)-C(3)-O(2)-C(4)	-179.0(4)
C(9)-C(4)-O(2)-C(3)	179.4(5)
C(5)-C(4)-O(2)-C(3)	0.2(4)

Symmetry transformations used to generate equivalent atoms:

Computational methodology

All the electronic-structure calculations were performed using density functional theory (DFT) as implemented in the Gaussian09 package.[1] Geometry optimizations without symmetry constraints were carried out with the hybrid local exchange-correlation M06-L functional[2] in conjunction with a mixed basis set: Pople's 6-311G(d) triple-ζ quality basis set with one polarization function for most of the light elements (C, H, O, Cl and those S and N that does not bind datively to the metal) and 6-311G(2d), the same basis set with two polarization functions for those S and N atoms which coordinate to the metal center, and for Br, I and Pt we have used the LANL08(d) and LANL2TZ(f) relativistic pseudopotentials,[3] respectively, for describing as accurate as possible the inner core of the heavy atoms. We called this level as M06-L/*mix-basis*. Subsequent harmonic frequency calculations were executed to corroborate the character of each optimized species, presumed to be critical points on the potential energy surface for two reasons: first, to get ensured we have analyzed true minima points on the potential energy surface; second, in order to obtain the thermal and entropic corrections for expressing the total energy, according to classical thermodynamics, as enthalpy and Gibbs free energy.

For improving the numerical precision in our reported self-consistent field (SCF) energies, we performed single-point calculations over each optimized geometry using the same M06-L functional but this time in conjunction with the Ahlrichs and coworkers' def2-tzvpp triple-ζ quality basis set.[4] Finally, we have also considered solvation effects added to the electronic Hamiltonian by performing single-point calculations over the optimized geometries at the same level of theory and using the SMD variation of IEFPCM of Truhlar and workers[5] with the dielectric standard values for dimethyl sulfoxide, in concordance with the experimental findings. Therefore, our final reported energy values are in solvent-phase. Thus, the final reported energies were calculated at the (SMD:dmso)M06-L/def2-tzvpp//M06-L/*mix-basis* level.

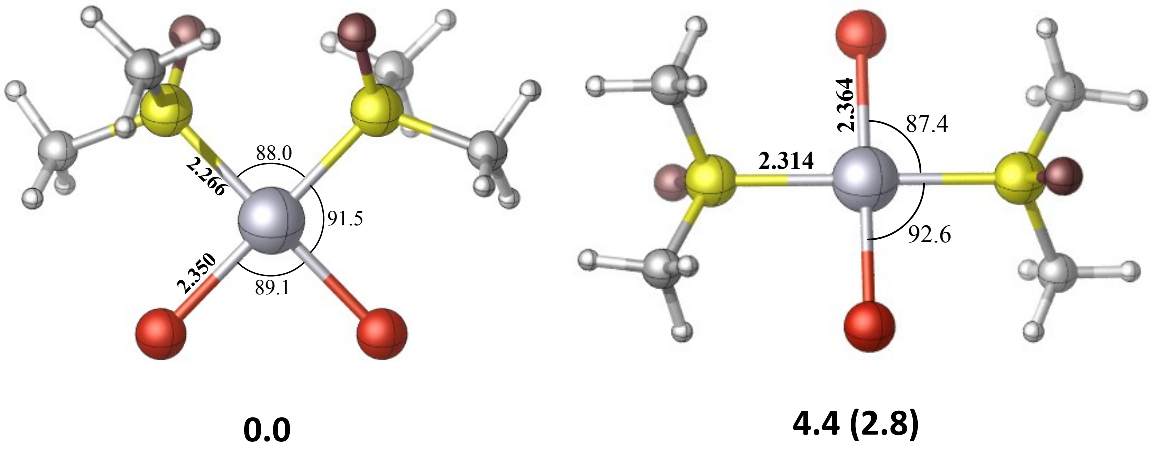

Fig. S32. Geometry comparison between both isomers of **Pt(Cl$_2$)(dmso)$_2$**. Bond distances (bold) are shown in angstroms, bond angles in degrees and relative energies (enthalpy; Gibbs free energy in parenthesis) in kcal·mol^{-1}.

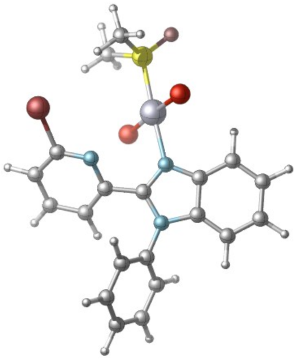

Pt(Cl$_2$)(dmso)(L1) isomer1
0.0

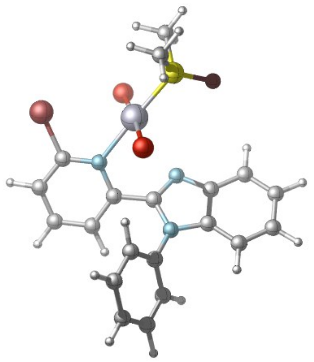

Pt(Cl$_2$)(dmso)(L1) isomer2
7.7 (7.7)

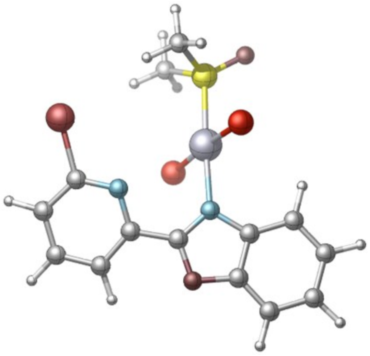

Pt(Cl$_2$)(dmso)(L2) isomer1
0.0

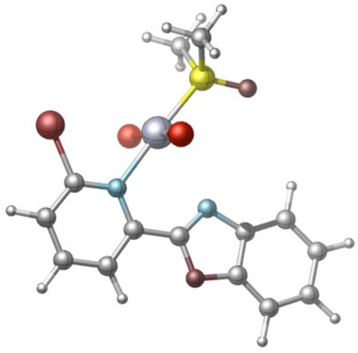

Pt(Cl$_2$)(dmso)(L2) isomer2
6.0 (3.4)

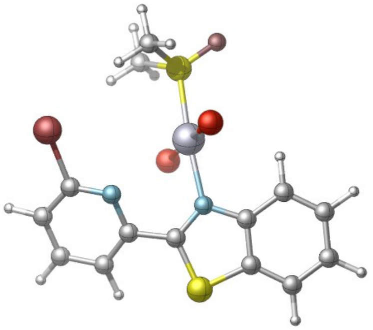

Pt(Cl$_2$)(dmso)(L3) isomer1
0.0

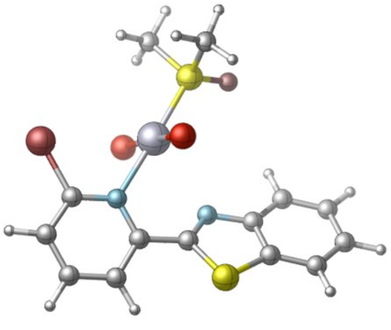

Pt(Cl$_2$)(dmso)(L3) isomer2
4.7 (4.0)

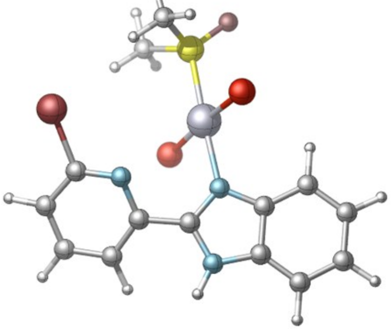

Pt(Cl$_2$)(dmso)(L4) isomer1
0.0

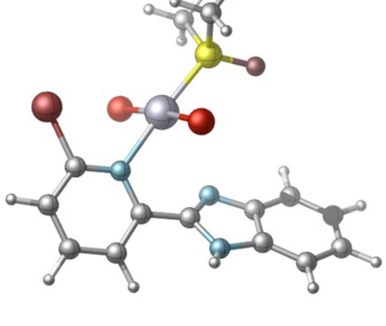

Pt(Cl$_2$)(dmso)(L4) isomer2
9.8 (9.4)

Fig. S33. Relative energies (enthalpy; Gibbs free energy in parenthesis) of 2-(6-bromo-2-pyridyl)-benz-(imida, oxa and othia)-zole coordination isomers given in kcal·mol^{-1}.

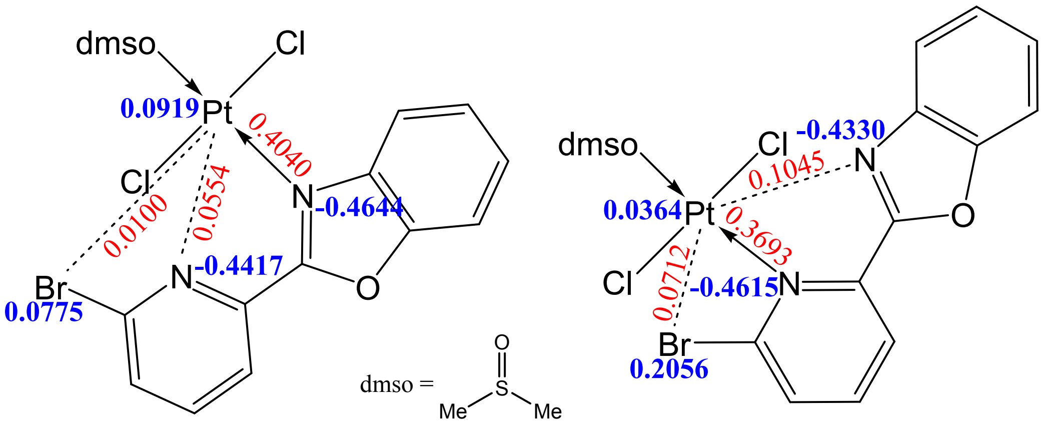

Fig. S34. NBO charges (bold blue) and Wiberg bond indices (red) for both **Pt(Cl$_2$)(dmso)(L2)** isomers calculated at the M06-L/*mix-basis* level.

Table S7. Donor-acceptor Natural Bond Orbitals showing the interaction energy calculated using the second-order perturbation theory from the NBO analysis. Values are in kcal·mol^{-1}.

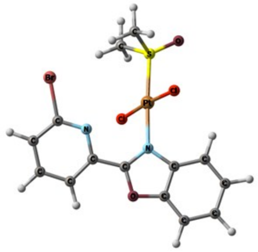	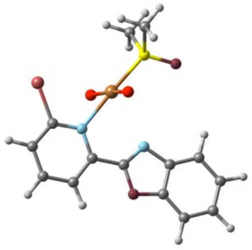
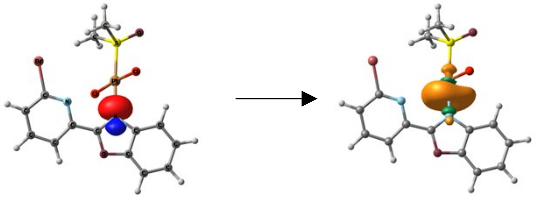 Donation LP(N1) to Pt **76.79**	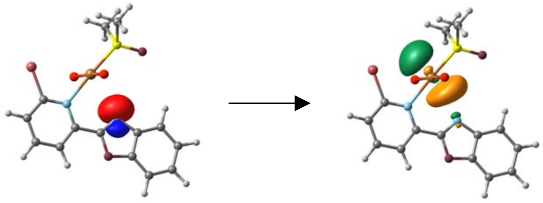 Donation LP(N1) to Pt **20.27**
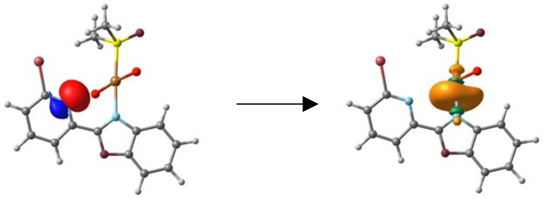 Donation LP(N2) to Pt **7.15**	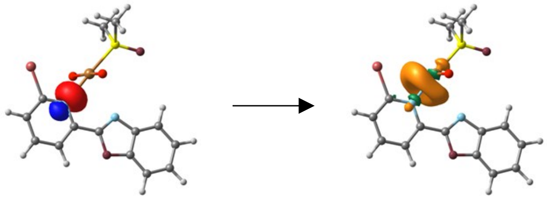 Donation LP(N2) to Pt **54.09**

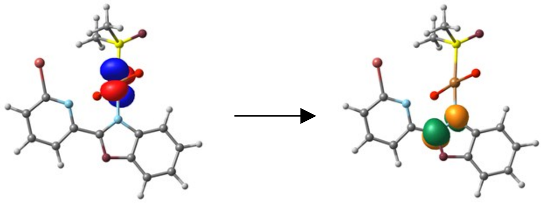<br>Back-donation Pt to N<br>**6.99**	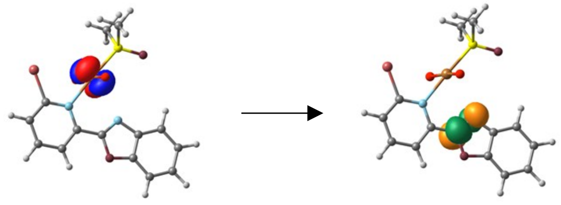<br>Back-donation Pt to N<br>**0.78**
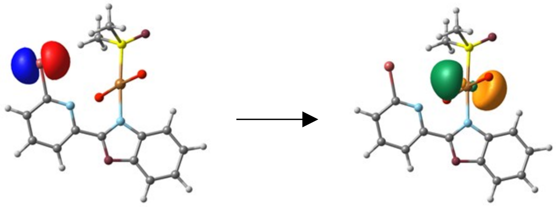<br>Donation LP(Br) to Pt<br>**0.61**	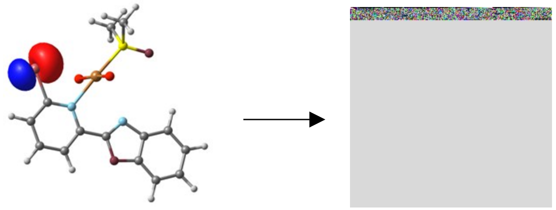<br>Donation LP(Br) to Pt<br>**11.15**

Table S8. Donor-acceptor Natural Bond Orbitals showing the Br $\rightarrow$ (C-H)* bond interactions energy calculated using the second-order perturbation theory from the NBO analysis found for complex **Pt(Cl$_2$)(dmso)(L$_2$)** isomer1.* Values are in kcal·mol^{-1}.

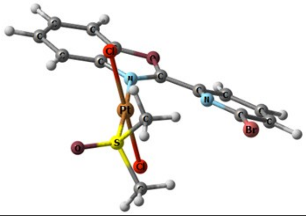
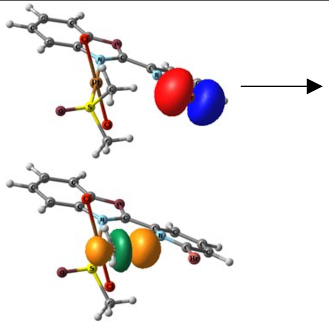<br>**0.51**
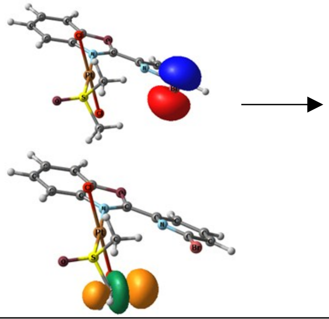

0.13

*For the rest of complexes, the trend is very similar.

Table S8. Cartesian coordinates (x-y-z format) of the optimized geometries at the M06-L/*mix-basis* level.

cis-**Pt(Cl$_2$)(dmso)$_2$**

E(scf) = –2146.16105580 a.u.

C	-3.200797	0.529605	-0.522057
H	-3.123504	0.022345	-1.482421
H	-3.836371	1.414280	-0.594727
H	-3.552963	-0.173074	0.230721
C	-1.853500	1.725153	1.565970
H	-2.132110	0.878550	2.195701
H	-2.637677	2.484445	1.538390
H	-0.906117	2.154760	1.892506
Cl	-1.647809	-2.176099	0.017402
Cl	1.648299	-2.175872	-0.017294
O	-1.259071	2.267560	-0.974800
Pt	0.000119	-0.500921	-0.000116
S	-1.571674	1.129148	-0.095991
O	1.258823	2.266874	0.976229
S	1.571403	1.129417	0.096177
C	3.201050	0.530055	0.520694
H	3.836322	1.414913	0.593737
H	3.124522	0.021939	1.480668
H	3.553011	-0.171894	-0.232852
C	1.852035	1.727164	-1.565358
H	0.904153	2.156343	-1.891014
H	2.131083	0.881372	-2.195997
H	2.635633	2.487028	-1.537397

trans-**Pt(Cl$_2$)(dmso)$_2$**

E(scf) = –2146.15673133 a.u.

C	-3.017160	-1.537150	0.266721
H	-2.719023	-2.260013	-0.490616
H	-4.101104	-1.409660	0.281909
H	-2.626416	-1.849650	1.235481
C	-2.911389	1.052480	1.159963
H	-2.517956	0.662955	2.100625
H	-4.002617	1.031443	1.139553
H	-2.531746	2.059132	0.988603
Cl	0.061371	-2.362296	0.052950
Cl	-0.061427	2.362305	-0.052689
O	-2.893323	0.487119	-1.450471
Pt	-0.000020	-0.000005	0.000054
S	-2.306676	0.039905	-0.186785
O	2.893609	-0.486561	1.450445
S	2.306756	-0.039850	0.186675
C	2.911392	-1.052922	-1.159742
H	4.002617	-1.031579	-1.139590
H	2.532064	-2.059591	-0.987809
H	2.517634	-0.663972	-2.100504
C	3.017056	1.537061	-0.267654
H	2.719518	2.260123	0.489722
H	2.625602	1.849324	-1.236212
H	4.100984	1.409535	-0.283609

L1 (X = NPh)

E(scf) = –870.637958254 a.u.

C	-0.447455	-0.699478	0.292793
C	-2.619341	-1.071167	0.024410
C	-1.889427	-2.269486	0.163561

L2 (X = O)

E(scf) = –659.443671854 a.u.

C	1.030633	0.286723	0.000261
C	3.176292	0.509346	-0.000163
C	2.817285	-0.842979	0.000208

C	-2.554720	-3.497247	0.110812
H	-1.998560	-4.424640	0.219794
C	-3.924061	-3.486343	-0.094349
H	-4.467296	-4.427186	-0.141662
C	-4.630707	-2.282427	-0.253033
H	-5.704405	-2.314964	-0.422450
C	-3.992371	-1.051459	-0.200846
H	-4.540884	-0.122245	-0.337335
C	0.805679	0.029202	0.522351
C	3.061555	0.053898	0.235256
C	3.230845	1.239310	0.949261
H	4.213778	1.678067	1.086726
C	2.084718	1.817621	1.473344
H	2.153400	2.735264	2.052997
C	0.852756	1.212001	1.263591
H	-0.053225	1.640687	1.682578
Br	4.627266	-0.795116	-0.501892
N	-0.548436	-2.005906	0.328990
N	1.914087	-0.540093	0.014342
N	-1.675025	-0.059826	0.116647
C	-1.922661	1.312650	-0.146149
C	-1.196162	1.978567	-1.132334
C	-2.897468	1.990107	0.584697
C	-1.434847	3.325323	-1.370884
H	-0.448302	1.434185	-1.704659
C	-3.140499	3.334190	0.327789
H	-3.447873	1.461910	1.359868
C	-2.407892	4.005957	-0.645174
H	-0.864322	3.842489	-2.138535
H	-3.901537	3.860428	0.899095
H	-2.597113	5.058532	-0.840620

C	3.807801	-1.824943	0.000250
H	3.544446	-2.878689	0.000488
C	5.127558	-1.397101	0.000016
H	5.928028	-2.132830	0.000099
C	5.459305	-0.033641	-0.000332
H	6.506587	0.257314	-0.000497
C	4.483213	0.958552	-0.000475
H	4.733302	2.015178	-0.000739
C	-0.337314	0.786606	0.000243
C	-2.547289	0.267758	0.000042
C	-2.946821	1.605584	0.000065
H	-3.997117	1.877593	0.000035
C	-1.940775	2.558809	0.000118
H	-2.194243	3.616167	0.000084
C	-0.611809	2.155403	0.000225
H	0.198327	2.877684	0.000256
Br	-3.912745	-1.088633	-0.000163
N	1.436931	-0.943876	0.000458
N	-1.305710	-0.147688	0.000107
O	2.024965	1.241248	-0.000089

L3 (X = S)

E(scf) = –982.409821300 a.u.

C	0.869895	0.472226	-0.000050
C	3.319047	0.417201	-0.000209
C	2.645703	-0.828695	-0.000054
C	3.394171	-2.012501	-0.000003
H	2.874121	-2.966713	0.000120
C	4.774436	-1.934760	-0.000113
H	5.364865	-2.847474	-0.000076
C	5.426526	-0.693596	-0.000272

L4 (X = NH)

E(scf) = –639.576212225 a.u.

C	-1.025498	0.400651	-0.001342
C	-3.239644	0.546467	0.007137
C	-2.795478	-0.795391	-0.006125
C	-3.733898	-1.833024	-0.012768
H	-3.403254	-2.868149	-0.023052
C	-5.076671	-1.495695	-0.005841
H	-5.827278	-2.282504	-0.010876
C	-5.496712	-0.154170	0.007434

H	6.513268	-0.658061	-0.000359
C	4.709927	0.492997	-0.000322
H	5.221684	1.452006	-0.000447
C	-0.539924	0.876940	0.000003
C	-2.712721	0.205360	0.000125
C	-3.211507	1.508811	0.000104
H	-4.278929	1.703386	0.000147
C	-2.275543	2.530820	0.000025
H	-2.600611	3.568374	0.000005
C	-0.922338	2.219920	-0.000025
H	-0.178850	3.013264	-0.000084
Br	-3.972745	-1.252631	0.000244
N	1.276330	-0.758309	0.000032
N	-1.444737	-0.119715	0.000081
S	2.152709	1.699697	-0.000242

$Pt(Cl_2)$(dmso)(L1) isomer1
E(scf) = –2463.59829279 a.u.

C	-4.045398	0.382818	1.583791
H	-3.699795	0.061502	2.564389
H	-5.135122	0.437301	1.549034
H	-3.581106	1.336875	1.329384
C	-4.235899	-0.146404	-1.087781
H	-3.752181	0.813869	-1.277651
H	-5.312135	-0.038106	-0.939290
H	-4.016278	-0.835647	-1.901748
C	1.614544	-0.016304	0.029711
C	2.836186	-1.863392	-0.165888
C	1.470666	-2.184853	-0.169856
C	1.048637	-3.508991	-0.280735
H	-0.010252	-3.750329	-0.287821
C	2.032648	-4.474696	-0.404936
H	1.742740	-5.517901	-0.498654
C	3.397762	-4.140847	-0.424951
H	4.135898	-4.930938	-0.536698
C	3.827721	-2.828551	-0.307352
H	4.881976	-2.565765	-0.329911
C	1.225205	1.380797	0.221809
C	-0.379994	2.931634	-0.192474
C	0.214498	3.889270	0.626533
H	-0.235850	4.865918	0.769761

H	-6.561088	0.068291	0.012428
C	-4.587587	0.892712	0.014257
H	-4.919454	1.928425	0.024397
C	0.361391	0.855111	-0.003254
C	2.559971	0.263822	-0.000798
C	3.013343	1.583490	-0.005676
H	4.072648	1.817451	-0.006395
C	2.040820	2.570545	-0.009972
H	2.328796	3.619070	-0.014448
C	0.699957	2.211984	-0.009023
H	-0.060283	2.989514	-0.014851
Br	3.871352	-1.147566	0.004930
N	-1.421316	-0.852930	-0.010919
N	1.304645	-0.105743	0.000711
N	-2.085113	1.289807	0.009142
H	-2.034872	2.295776	0.023521

$Pt(Cl_2)$(dmso)(L1) isomer2
E(scf) = –2463.58573193 a.u.

C	-4.041503	2.305782	0.010991
H	-3.865604	2.536320	-1.037791
H	-4.552110	3.128538	0.514747
H	-4.594324	1.368920	0.084843
C	-2.944660	1.858354	2.483388
H	-3.548614	0.951429	2.537805
H	-3.502677	2.737751	2.811092
H	-2.033721	1.728935	3.066428
C	1.571855	0.049906	-0.870371
C	3.035429	1.680977	-0.586226
C	1.821763	2.154967	-1.124563
C	1.649524	3.519879	-1.370324
H	0.707214	3.889932	-1.763852
C	2.701529	4.364881	-1.065632
H	2.597105	5.433719	-1.235351
C	3.902961	3.876006	-0.522166
H	4.701449	4.576025	-0.287531
C	4.093085	2.526562	-0.266017
H	5.014855	2.155255	0.175133
C	1.005421	-1.296107	-0.894976
C	-0.842128	-2.661370	-0.552072
C	-0.131716	-3.799612	-0.916133
H	-0.620829	-4.767071	-0.895711

C	1.397933	3.526558	1.251855
H	1.900511	4.228306	1.912894
C	1.921121	2.255003	1.053806
H	2.825007	1.938610	1.566455
Br	-2.038817	3.364340	-1.072493
Cl	-0.934582	0.100305	2.299367
Cl	-1.544704	-1.901389	-1.954197
N	0.746144	-1.014149	-0.050977
N	0.093424	1.731350	-0.410780
O	-4.250686	-2.108715	0.705497
Pt	-1.321221	-0.896066	0.175398
S	-3.542216	-0.863662	0.400346
N	2.902612	-0.481849	-0.032601
C	4.096304	0.292919	-0.086890
C	5.098124	0.062032	0.852238
C	4.247793	1.272389	-1.064925
C	6.256078	0.829666	0.816584
H	4.954323	-0.701706	1.612917
C	5.404343	2.041159	-1.084625
H	3.458854	1.427258	-1.797278
C	6.407838	1.823035	-0.145561
H	7.037987	0.655898	1.551497
H	5.524372	2.809637	-1.843883
H	7.311814	2.426302	-0.166171

C	1.194514	-3.656502	-1.287204
H	1.775655	-4.524761	-1.586104
C	1.769301	-2.397251	-1.270994
H	2.805404	-2.250107	-1.559008
Br	-2.633196	-2.876001	0.018716
Cl	-2.669791	0.156632	-1.912904
Cl	-0.020423	0.275043	2.027985
N	0.940329	1.115008	-1.302259
N	-0.305020	-1.434082	-0.547466
O	-1.726322	3.396540	0.690234
Pt	-1.401020	0.296349	0.079195
S	-2.438342	2.120221	0.785024
N	2.864581	0.313888	-0.438188
C	3.761762	-0.553932	0.241476
C	5.072332	-0.673396	-0.217662
C	3.328747	-1.273696	1.353853
C	5.953263	-1.523563	0.439443
H	5.387404	-0.106631	-1.091091
C	4.214764	-2.132230	1.992421
H	2.309465	-1.136368	1.712028
C	5.524885	-2.259063	1.540124
H	6.976677	-1.617011	0.083726
H	3.880761	-2.694410	2.861174
H	6.214972	-2.927246	2.049830

Pt(Cl_2)(dmso)(L2) isomer1
E(scf) = –2252.39586805 a.u.

C	-2.489134	-2.312153	1.575149
H	-1.988518	-2.307315	2.541497
H	-3.238536	-3.104381	1.525094
H	-2.917639	-1.327458	1.383751
C	-2.288681	-2.676603	-1.128341
H	-2.719226	-1.681743	-1.255764
H	-3.058587	-3.439800	-0.999300
H	-1.639701	-2.908707	-1.971519
C	1.407917	1.830196	-0.060134
C	3.559141	1.596220	-0.087161
C	2.964260	0.337797	-0.117007
C	3.735344	-0.819280	-0.155892
H	3.267617	-1.799230	-0.189693
C	5.110379	-0.640684	-0.165256
H	5.757328	-1.512825	-0.198683

Pt(Cl_2)(dmso)(L2) isomer2
E(scf) = –2252.38339701 a.u.

C	-1.732231	-3.443405	1.245856
H	-1.228805	-3.219254	2.184514
H	-1.821785	-4.520472	1.091740
H	-2.702510	-2.945596	1.230194
C	-1.671245	-3.321969	-1.490340
H	-2.650932	-2.843198	-1.456080
H	-1.748231	-4.410405	-1.457360
H	-1.134193	-2.987883	-2.376449
C	1.910120	1.379396	0.079025
C	4.045883	1.104124	-0.001243
C	3.399660	-0.120417	0.198855
C	4.138579	-1.298707	0.303862
H	3.639977	-2.253078	0.447554
C	5.516894	-1.183196	0.205197
H	6.133828	-2.075049	0.277931

C	5.693229	0.636232	-0.135298
H	6.776401	0.723813	-0.144018
C	4.925284	1.795367	-0.095841
H	5.366678	2.786661	-0.072040
C	0.147608	2.548494	-0.019390
C	-2.101944	2.400147	-0.252821
C	-2.307794	3.723557	0.141859
H	-3.307331	4.140601	0.207287
C	-1.180111	4.472214	0.442148
H	-1.283369	5.509564	0.750578
C	0.077295	3.885235	0.364868
H	0.978559	4.436341	0.614567
Br	-3.631787	1.313490	-0.683069
Cl	-0.309463	-0.072620	2.260550
Cl	0.818820	-1.773624	-2.014600
N	1.590489	0.535008	-0.091354
N	-0.933348	1.824701	-0.350162
O	-0.739826	-4.026588	0.562848
O	2.558407	2.537213	-0.050969
Pt	0.177553	-0.981554	0.119820
S	-1.253418	-2.675231	0.333927

$Pt(Cl_2)$(dmso)(L3) isomer1

E(scf) = –2575.36321278 a.u.

C	-2.709745	-2.042034	1.639578
H	-2.210994	-1.982182	2.605025
H	-3.493129	-2.802350	1.645495
H	-3.096360	-1.058447	1.367777
C	-2.525979	-2.623643	-1.024353
H	-2.923335	-1.628330	-1.231446
H	-3.319324	-3.348048	-0.830375
H	-1.890817	-2.943613	-1.848380
C	1.460901	1.765649	-0.057412
C	3.824264	1.122681	-0.178102
C	2.909307	0.054549	-0.153647
C	3.369029	-1.265617	-0.174904
H	2.655950	-2.084875	-0.156042
C	4.732676	-1.481059	-0.229036
H	5.108645	-2.500281	-0.248768
C	5.638545	-0.411436	-0.259667
H	6.706073	-0.612670	-0.300716
C	5.198459	0.901174	-0.233643

C	6.142629	0.059047	0.009611
H	7.226672	0.099252	-0.060820
C	5.417457	1.241008	-0.100413
H	5.896537	2.202686	-0.256384
C	0.699931	2.181430	0.036557
C	-1.611378	2.315116	-0.001596
C	-1.581087	3.706490	-0.028590
H	-2.511469	4.262322	-0.054987
C	-0.350162	4.337450	-0.027719
H	-0.290153	5.422198	-0.051475
C	0.801054	3.569746	0.004747
H	1.785637	4.022670	0.009216
Br	-3.303272	1.472646	-0.023629
Cl	-1.015837	-0.486174	2.336012
Cl	-0.339944	-0.427082	-2.355857
N	2.038972	0.104079	0.252292
N	-0.509759	1.555309	0.027842
O	0.567273	-3.507180	-0.073654
O	3.088251	2.073825	-0.077019
Pt	-0.656891	-0.582092	-0.006995
S	-0.712475	-2.792114	-0.073497

$Pt(Cl_2)$(dmso)(L3) isomer2

E(scf) = –2575.35364030 a.u.

C	1.954307	-3.523740	-0.405814
H	2.041112	-3.238452	-1.453832
H	1.898493	-4.607950	-0.289911
H	2.781559	-3.096390	0.163055
C	0.462138	-3.354046	1.894345
H	1.316336	-2.901012	2.398120
H	0.514076	-4.444483	1.891474
H	-0.456025	-3.005549	2.363883
C	-1.596447	1.706914	-0.206337
C	-3.945829	1.111927	0.066673
C	-3.118019	0.162189	-0.579720
C	-3.655066	-1.056461	-1.011448
H	-3.003026	-1.782224	-1.489973
C	-4.998163	-1.299028	-0.795936
H	-5.432430	-2.241647	-1.118940
C	-5.811907	-0.348220	-0.161214
H	-6.866154	-0.564637	-0.005240
C	-5.300243	0.862748	0.278286

H	5.900919	1.729635	-0.251862
C	0.180321	2.465091	0.039481
C	-2.063907	2.339448	-0.278186
C	-2.302462	3.582248	0.307315
H	-3.309853	3.972413	0.407423
C	-1.193792	4.283819	0.755645
H	-1.318726	5.256446	1.224494
C	0.071071	3.723198	0.628507
H	0.947672	4.235950	1.016080
Br	-3.568627	1.306562	-0.895890
Cl	-0.293291	-0.026282	2.315317
Cl	0.472628	-1.698125	-2.050929
N	1.587597	0.459591	-0.088640
N	-0.884454	1.797318	-0.432688
O	-1.025243	-3.895436	0.760323
S	2.964832	2.631733	-0.123430
Pt	0.028329	-0.937871	0.146019
S	-1.487664	-2.545091	0.432845

Pt(Cl$_2$)(dmso)(L4) isomer1

E(scf) = –2232.53533388 a.u.

C	-2.566725	-2.146147	1.561195
H	-2.119633	-2.077513	2.550939
H	-3.324865	-2.930917	1.525738
H	-2.970242	-1.173269	1.275929
C	-2.237199	-2.704305	-1.090265
H	-2.650791	-1.717673	-1.307520
H	-3.020567	-3.450364	-0.942808
H	-1.553935	-2.999561	-1.885178
C	1.405613	1.865441	-0.068772
C	3.614259	1.614576	-0.177318
C	2.980381	0.359873	-0.147773
C	3.724918	-0.818731	-0.174470
H	3.226616	-1.783983	-0.154128
C	5.100989	-0.691946	-0.246730
H	5.715849	-1.587247	-0.275126
C	5.726295	0.566424	-0.288846
H	6.810407	0.618206	-0.347575
C	4.996735	1.744170	-0.253713
H	5.483469	2.715751	-0.281761
C	0.105083	2.514949	0.026101
C	-2.129016	2.308517	-0.309468

H	-5.937792	1.589433	0.774545
C	-0.279682	2.333675	-0.162742
C	2.024307	2.087277	-0.080788
C	2.224126	3.463884	-0.099879
H	3.232807	3.859690	-0.069879
C	1.113553	4.288306	-0.144600
H	1.233539	5.368294	-0.161785
C	-0.147836	3.718365	-0.172887
H	-1.039752	4.335148	-0.227505
Br	3.553287	0.977203	0.036725
Cl	1.272694	-0.809299	-2.224795
Cl	-0.092338	-0.217435	2.277163
N	-1.809499	0.538991	-0.721504
N	0.812165	1.519474	-0.113605
O	-0.702084	-3.490411	-0.478187
S	-3.002850	2.497808	0.511248
Pt	0.585759	-0.619147	0.038986
S	0.418070	-2.818623	0.187069

Pt(Cl$_2$)(dmso)(L4) isomer2

E(scf) = –2232.51951469 a.u.

C	0.974319	-3.791898	-0.897002
H	0.650035	-3.562069	-1.909949
H	0.767028	-4.833647	-0.644993
H	2.031476	-3.546410	-0.792447
C	0.640983	-3.360104	1.788461
H	1.710099	-3.149344	1.842343
H	0.433699	-4.429680	1.861258
H	0.115220	-2.801780	2.561741
C	-1.540091	1.740064	-0.254372
C	-3.702650	1.537027	0.152004
C	-3.221517	0.498590	-0.676084
C	-4.086937	-0.507647	-1.113247
H	-3.714094	-1.317186	-1.734493
C	-5.405613	-0.444917	-0.700047
H	-6.102868	-1.218472	-1.012280
C	-5.867850	0.593349	0.128063
H	-6.911449	0.602765	0.433005
C	-5.027678	1.603887	0.570535
H	-5.392066	2.401070	1.214261
C	-0.203927	2.315344	-0.186015
C	2.089753	2.004158	-0.033288

C	-2.418543	3.533065	0.292205	C	2.326950	3.375511	-0.033734
H	-3.440847	3.881929	0.392957	H	3.344684	3.742577	0.033516
C	-1.341319	4.269310	0.760125	C	1.241892	4.231199	-0.107532
H	-1.507272	5.227000	1.247029	H	1.393295	5.307544	-0.115582
C	-0.055414	3.757699	0.635545	C	-0.033925	3.696596	-0.177531
H	0.793283	4.289363	1.059289	H	-0.906460	4.336791	-0.267973
Br	-3.592133	1.226700	-0.940927	Br	3.577465	0.852806	0.158800
Cl	-0.346116	0.059865	2.258733	Cl	1.418570	-0.875056	-2.155274
Cl	0.869615	-1.832340	-1.915225	Cl	-0.369380	-0.177640	2.184207
N	1.613376	0.557964	-0.082936	N	-1.875250	0.659198	-0.915831
N	-0.929093	1.813625	-0.466523	N	0.863461	1.471619	-0.116480
O	-0.791217	-3.947476	0.764419	O	-1.399336	-3.165126	0.099567
N	2.590993	2.538630	-0.108032	N	-2.609529	2.347760	0.371881
Pt	0.192315	-0.941615	0.168966	Pt	0.504831	-0.628863	0.012260
S	-1.270797	-2.608316	0.416356	S	0.008097	-2.777068	0.217073
H	2.682309	3.535802	-0.225809	H	-2.530829	3.023816	1.117493

Pt(Cl$_2$)(L1)

E(scf) = –1910.33717298 a.u.

C	1.227464	-0.123429	-0.036780
C	2.768534	1.431799	0.302242
C	1.487979	2.001901	0.425319
C	1.340741	3.350935	0.755511
H	0.352735	3.786492	0.851632
C	2.499445	4.084127	0.938988
H	2.423158	5.137633	1.194535
C	3.773971	3.504874	0.810296
H	4.655875	4.120525	0.968721
C	3.934367	2.165055	0.496272
H	4.915621	1.705681	0.409824
C	0.478355	-1.339049	-0.230849
C	-1.623366	-2.265458	0.136874
C	-1.145314	-3.538971	-0.168830
H	-1.812264	-4.388567	-0.073395
C	0.169707	-3.687633	-0.572475
H	0.552593	-4.665763	-0.850102
C	1.006818	-2.581675	-0.558244
H	2.057363	-2.678777	-0.804278
Br	-3.341753	-2.140833	0.897331
Cl	-3.570228	0.546364	-1.014125
Cl	-1.761933	3.106177	-0.491015
N	0.557248	1.006264	0.205396

Pt(Cl$_2$)(L2)

E(scf) = –1699.13567256 a.u.

C	1.227683	1.547074	-0.199727
C	3.364138	1.505945	0.014914
C	2.869623	0.219424	0.249100
C	3.734858	-0.821876	0.576765
H	3.354375	-1.821403	0.754175
C	5.083843	-0.505973	0.645166
H	5.795537	-1.288219	0.894764
C	5.559631	0.790866	0.400759
H	6.626770	0.986088	0.466249
C	4.702340	1.837139	0.079222
H	5.055382	2.846171	-0.107811
C	-0.115540	2.009217	-0.336666
C	-2.316855	1.418341	0.044671
C	-2.726033	2.726993	-0.223914
H	-3.777095	2.974695	-0.124017
C	-1.791177	3.683736	-0.580459
H	-2.101668	4.701469	-0.798799
C	-0.451402	3.328850	-0.594699
H	0.337049	4.048759	-0.789757
Br	-3.607333	0.247717	0.754132
Cl	-1.951398	-2.174217	-0.766534
Cl	1.093326	-2.841683	-0.154877
N	1.487444	0.292657	0.099963

N	-0.873711	-1.156665	-0.004486	N	-1.040682	1.013777	-0.099500
Pt	-1.422620	0.846690	-0.185129	O	2.308878	2.343787	-0.270380
N	2.582907	0.087736	0.011654	Pt	-0.139634	-0.899758	-0.119441
C	3.647259	-0.857420	-0.094270				
C	4.484663	-0.813246	-1.204716				
C	3.851947	-1.789814	0.919288				
C	5.528385	-1.725900	-1.307467				
H	4.307220	-0.071904	-1.980215				
C	4.890249	-2.705275	0.801955				
H	3.192310	-1.796573	1.783974				
C	5.726946	-2.675182	-0.310232				
H	6.182873	-1.698548	-2.174784				
H	5.050844	-3.439822	1.586870				
H	6.540125	-3.391434	-0.397141				

Pt(Cl_2)(L3)

E(scf) = –2022.10542201 a.u.

C	1.144662	1.536419	0.284526
C	3.531728	1.240573	-0.093882
C	2.750817	0.091480	-0.337110
C	3.354508	-1.070154	-0.829196
H	2.748011	-1.944083	-1.035373
C	4.722275	-1.062454	-1.028008
H	5.206409	-1.957866	-1.408324
C	5.494753	0.073714	-0.752743
H	6.569101	0.047311	-0.916259
C	4.909382	1.240895	-0.290159
H	5.503961	2.129214	-0.096196
C	-0.226209	1.954798	0.401925
C	-2.401054	1.348000	-0.137681
C	-2.856136	2.630328	0.167212
H	-3.902975	2.865945	0.010373
C	-1.962214	3.580022	0.633532
H	-2.305382	4.579076	0.886826
C	-0.618424	3.250614	0.707828
H	0.128150	3.987969	0.989689
Br	-3.599013	0.183707	-1.005252
Cl	-2.086192	-2.094011	0.887280
Cl	0.949717	-2.863808	0.483265
N	1.402971	0.289334	-0.085665
N	-1.126558	0.965674	0.073320
S	2.528048	2.568558	0.415553

Pt(Cl_2)(L4)

E(scf) = –1679.27493551 a.u.

C	-1.207562	1.600760	-0.235148
C	-3.405432	1.528295	0.023917
C	-2.849591	0.256101	0.278905
C	-3.664734	-0.811612	0.658618
H	-3.232210	-1.786553	0.852157
C	-5.022272	-0.564765	0.756909
H	-5.687131	-1.373777	1.047169
C	-5.567371	0.703415	0.492282
H	-6.640795	0.849477	0.581948
C	-4.770298	1.775280	0.125256
H	-5.191902	2.757609	-0.071113
C	0.156592	2.014692	-0.378951
C	2.338763	1.372966	0.064494
C	2.799547	2.653580	-0.243775
H	3.855751	2.870979	-0.129671
C	1.902506	3.622747	-0.658563
H	2.248673	4.620228	-0.913835
C	0.552457	3.309521	-0.680900
H	-0.190150	4.062811	-0.929787
Br	3.563136	0.190527	0.866750
Cl	1.944138	-2.148100	-0.821434
Cl	-1.111527	-2.811167	-0.260337
N	-1.479714	0.341997	0.103696
N	1.053029	1.003696	-0.099814
N	-2.344370	2.348472	-0.302865

Pt	-0.232179	-0.905408	0.186332

Pt	0.130676	-0.875926	-0.141166
H	-2.397365	3.335515	-0.497424

$Pt(Cl_2)(coe)(L2)$ isomer1

E(scf) = –2012.45613661 a.u.

C	2.729061	0.291236	-0.104325
C	3.916675	-1.513129	-0.151271
C	2.566476	-1.853539	-0.122686
C	2.165626	-3.185420	-0.131964
H	1.111092	-3.447375	-0.129972
C	3.176679	-4.134248	-0.166660
H	2.914343	-5.188471	-0.179451
C	4.532489	-3.771978	-0.190030
H	5.288827	-4.552095	-0.215982
C	4.938389	-2.441404	-0.184386
H	5.983868	-2.151467	-0.204255
C	2.460197	1.714454	-0.077782
C	0.836523	3.299671	-0.153195
C	1.744946	4.336607	0.078208
H	1.408506	5.366221	0.144075
C	3.080907	3.993517	0.215917
H	3.824626	4.766241	0.392713
C	3.459092	2.657931	0.144370
H	4.492164	2.349010	0.271789
Br	-1.023152	3.713568	-0.331769
Cl	0.057274	0.356852	2.319772
Cl	-0.381348	-1.469426	-2.023132
N	1.845128	-0.669946	-0.081699
N	1.168366	2.036563	-0.250353
O	4.008489	-0.142153	-0.142114
Pt	-0.268710	-0.537084	0.151889
C	-2.122222	-1.335158	0.821337
H	-1.969576	-1.412053	1.900805
C	-2.661690	-2.568282	0.168009
H	-1.980915	-3.415397	0.315643
H	-2.734114	-2.431077	-0.914204
C	-4.037639	-2.900844	0.757237
H	-4.461423	-3.736354	0.184025
H	-3.917228	-3.275501	1.782933
C	-5.028279	-1.737778	0.774380
H	-4.703055	-0.988260	1.509573
H	-5.977064	-2.120423	1.169000

***cis*-cyclooctene (coe)**

E(scf) = –313.270248284 a.u.

C	0.563765	-1.639860	-0.310514
H	1.118306	-2.286266	-0.993250
C	1.361557	-0.831100	0.659209
H	2.221133	-1.403872	1.028103
H	0.753673	-0.604646	1.545037
C	1.860731	0.486474	0.053292
H	2.343410	1.071746	0.848335
H	2.651423	0.277544	-0.680286
C	0.781194	1.329990	-0.619180
H	0.463846	0.842155	-1.550008
H	1.248066	2.270868	-0.935377
C	-0.458986	1.637946	0.234911
H	-0.205894	1.569570	1.304097
H	-0.739164	2.687680	0.083786
C	-1.706817	0.798268	-0.044676
H	-1.943773	0.856604	-1.116896
H	-2.556707	1.268925	0.467650
C	-1.655349	-0.673503	0.382680
H	-1.357022	-0.718873	1.439697
H	-2.674326	-1.077607	0.352788
C	-0.759642	-1.545880	-0.448699
H	-1.241688	-2.127843	-1.235810

DMSO

E(scf) = –553.210174289 a.u.

O	0.000506	1.478427	0.394134
S	0.000041	0.249345	-0.441992
C	-1.340777	-0.813789	0.177923
H	-1.252037	-0.904560	1.262797
H	-2.282266	-0.322592	-0.070053
H	-1.301647	-1.794923	-0.300764
C	1.340305	-0.814555	0.177878
H	2.282123	-0.324273	-0.070630
H	1.300201	-1.795945	-0.300185
H	1.251746	-0.904581	1.262831

C	-5.276589	-1.047569	-0.576864
H	-4.999616	-1.723278	-1.399805
H	-6.354521	-0.885907	-0.698090
C	-4.588265	0.302603	-0.782706
H	-4.843404	0.968818	0.055151
H	-5.018361	0.778035	-1.673514
C	-3.068296	0.258609	-0.970543
H	-2.816354	-0.469863	-1.746406
H	-2.720084	1.224363	-1.354382
C	-2.336205	-0.036683	0.308523
H	-2.338381	0.775553	1.039846

$Pt(Cl_4)^{2-}$

E(scf) = –1960.19517986 a.u.

Pt	0.000000	0.000000	0.000000
Cl	0.000000	2.401011	0.000000
Cl	2.401011	0.000000	0.000000
Cl	0.000000	-2.401011	0.000000
Cl	-2.401011	0.000000	0.000000

References

1. M. J. Frisch, G. W. Trucks, H. B. Schlegel, G. E. Scuseria, M. A. Robb, J. R. Cheeseman, G. Scalmani, V. Barone, B. Mennucci, G. A. Petersson, H. Nakatsuji, M. Caricato, X. Li, H. P. Hratchian, A. F. Izmaylov, J. Bloino, G. Zheng, J. L. Sonnenberg, M. Hada, M. Ehara, K. Toyota, R. Fukuda, J. Hasegawa, M. Ishida, T. Nakajima, Y. Honda, O. Kitao, H. Nakai, T. Vreven, J. A. Montgomery, Jr., J. E. Peralta, F. Ogliaro, M. Bearpark, J. J. Heyd, E. Brothers, K. N. Kudin, V. N. Staroverov, T. Keith, R. Kobayashi, J. Normand, K. Raghavachari, A. Rendell, J. C. Burant, S. S. Iyengar, J. Tomasi, M. Cossi, N. Rega, J. M. Millam, M. Klene, J. E. Knox, J. B. Cross, V. Bakken, C. Adamo, J. Jaramillo, R. Gomperts, R. E. Stratmann, O. Yazyev, A. J. Austin, R. Cammi, C. Pomelli, J. W. Ochterski, R. L. Martin, K. Morokuma, V. G. Zakrzewski, G. A. Voth, P. Salvador, J. J. Dannenberg, S. Dapprich, A. D. Daniels, O. Farkas, J. B. Foresman, J. V. Ortiz, J. Cioslowski, and D. J. Fox, Gaussian 09, Rev.C.01 Gaussian, Inc., Wallingford CT, 2010.
2. Y. Zhao and D. G. Truhlar, *J. Chem. Phys.* **2006**, *125*, 194101: 1-18.
3. a) P. J. Hay and W. R. Wadt, *J. Chem. Phys.* **1985**, *82*, 284-287; b) L. E. Roy, P. J. Hay, and R. L. Martin, *J. Chem. Theory Comput.* **2008**, *4*, 1029-1031; (c) C. E. Check, T. O. Faust, J. M. Bailey, B. J. Wright, T. M. Gilbert and L. S. Sunderlin, *J. Phys. Chem. A* **2001**, *105*, 8111-8115.

4. a) F. Weigend and R. Ahlrichs, *Phys. Chem. Chem. Phys.* **2005**, *7*, 3297-3305; b) F. Weigend, *Phys. Chem. Chem. Phys.* **2006**, *8*, 1057-1065.

5. A. V. Marenich, C. J. Cramer, and D. G. Truhlar, *J. Phys. Chem. B* **2009**, *113*, 6378-6396.
